# A Convenient One-Pot Preparation of 2-Methyl-3-(phenylthio- methyl)quinolines from Morita-Baylis-Hillman Adducts and Their Oxidation to the Corresponding Sulfones

**DOI:** 10.3390/molecules17055081

**Published:** 2012-05-03

**Authors:** Chintakunta Ramesh, Po-Min Lei, Veerababurao Kavala, Chun-Wei Kuo, Ching-Fa Yao

**Affiliations:** Department of Chemistry, National Taiwan Normal University, 88, Sec. 4, Tingchow Road, Taipei 116, Taiwan

**Keywords:** one-pot procedure, Morita-Baylis-Hillman adducts, quinolines, reductive cyclization, Fe/AcOH

## Abstract

A convenient one-pot preparation of 2-methyl-3-(phenylthiomethyl)quinolines from Morita-Baylis-Hillman adducts via conjugate addition of thiols followed by reductive cyclization with Fe/AcOH was developed. The 2-methyl-3-(phenylthiomethyl)quinolines were transformed into 2-methyl-3-(phenylsulfonylmethyl)quinolines via *m*-CPBA-mediated oxidation.

## 1. Introduction

**Figure 1:**
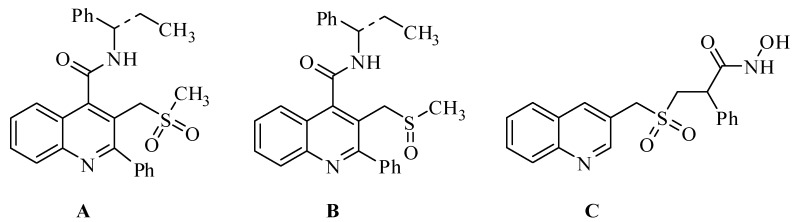
Biologically important C3-alkylsulfonyl or C3-alkylsulfoxide quinoline derivatives.

The quinoline nucleus is a ubiquitous heterocyclic structural motif that is found in many naturally occurring quinoline alkaloids, therapeutic and synthetic compounds with a wide spectrum of biological activities such as antimalarial, antidiabetic, anti-inflammatory, antiasthmatic, antihypertensive, antibacterial, tyrosine kinase inhibiting agents [1,2,3,4]. Quinoline and their derivatives have been utilized for the construction of nano- and meso structures having enhanced electronic and photonic properties [5]. In particular, the C3-alkylsulfone-containing quinolines posess important biological activities. Some of the important C3-alkylsulfonyl and C3-alkylsulfoxide quinoline derivatives are depicted in Figure 1 [6,7]. Compound **A** displayed excellent functional activity, compound **B** exhibits excellent *in vivo*/*in vitro* DMPK profile and acts as potent NK3 receptor antagonists [6]. Similarly, compound **C** acts as inhibitor of soluble human CD23 [7]. In this regard, C3-alkylthioquinoline derivatives are key precursors for the preparation of C3-alkylsulfonylquinoline derivatives. The C3-alkylthioquinolines are easily transformed into the corresponding C3-alkylsulfonylquinolines via oxidation reactions. Many protocols have been developed for the synthesis of quinoline derivatives [8,9,10,11,12,13,14,15,16,17,18]. However, the synthesis of C3-alkylthioquinoline and their derivatives are very rare [6]. Therefore, an easy and simple synthesis of C3-alkylthioquinolines and their derivatives is still needed.

The Morita-Baylis-Hillman (MBH) reaction is a useful carbon-carbon bond forming reaction, which has the propensity to afford densely functionalized adducts [19,20,21]. Thiol addition to Morita-Baylis-Hillman adducts is well explored in the literature [22,23,24,25]. However, to our knowledge, the synthesis of C3-alkylthioquinolines and their derivatives from the MBH adducts is not known. Recently, Kim *et al.* attempted the synthesis of C3-arylthiomethylquinoline derivatives from (*Z*)-methyl 3-(2-azidophenyl)-2-(phenylthiomethyl)acrylate (MBH adduct) using the aza-Wittig reaction, but they failed to obtain the desired product, obtaining instead methylquinoline-3-carboxylate. However, they obtained phenylsulfinylmethyl-containing quinolines from MBH adducts by the aza-Wittig reaction in a multi-step synthetic route [22]. As part of our ongoing project on the synthesis of nitrogen-containing heterocyclic molecules via reductive cyclization [26,27,28,29,30], herein, we wish to report a convenient one-pot procedure for the preparation of C3-arylthiomethylquinolines from the MBH adducts and the transformation of the reaction products into the corresponding C3-arylsulfonylmethyl-containing quinoline derivatives via an oxidation reaction.

## 2. Results and Discussion

**Scheme 1:**
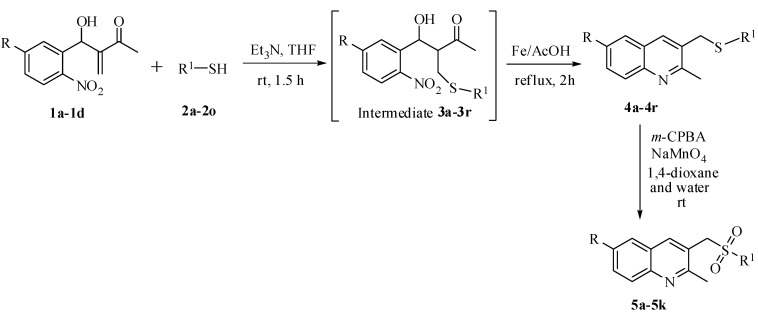
Outline of our synthetic route for the synthesis of 2-methyl-3-(phenylthiomethyl) quinoline derivatives and 2-methyl-3-(phenylsulfonylmethyl)quinoline derivatives.

Recently, we developed an easily accessible method for the synthesis of indolylquinoline derivatives from MBH adducts [26]. Taking cues from this reaction, we envisioned synthesizing C3-arylthiomethylquinoline derivatives from MBH adducts derived from the reaction of 2-nitrobenzaldehydes and methyl vinyl ketone. Our synthetic strategy for accessing 2-methyl-3-(phenylsulfonylmethyl)quinoline derivatives is outlined in Scheme 1. The starting MBH adducts were prepared according to a previously reported procedure [31]. Initially, the MBH adduct **1a** was treated with benzenethiol **2a** in the presence of triethylamine in THF at room temperature to yield intermediate compound **3a**, which was treated with Fe/AcOH heated under reflux conditions to give the corresponding 2-methyl-3-(phenylthiomethyl)quinoline compound **4a**. Subsequently, sulfide **4a** was subjected to oxidation with a stoichiometric amount of 3-chloroperoxybenzoic acid (*m-*CPBA) and sodium permanganate in 1,4-dioxane/water (1:1) at room temperature for 20 min, to provide the expected the 2-methyl-3-(phenylsulfonylmethyl)quinoline compound **5a**. The first two steps of Scheme 1 were conducted in a one-pot operation.

**Figure 2:**
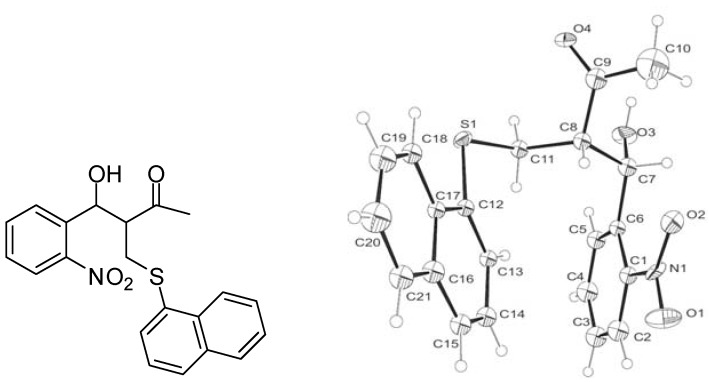
Crystal structure of the 4-hydroxy-3-((naphthalen-1-ylthio)methyl)-4-(2-nitrophenyl)butan-2-one intermediate **3h** (relative configuration is 7S* and 8S*) [32].

A literature survey revealed that the addition of thiols to MBH acetates takes place either at the γ-position via a S_N_2' reaction [22,23,24,25] or mixture of the products [major product at γ-position (E/Z = 10:1) and trace amount of product at α-position] [25]. In order to determine the structure of the intermediates **3a**–**3r** in our process, we conducted the reaction of MBH adduct **1a** with naphthalene thiol **2h** in the presence of triethylamine. The intermediate **3h** obtained from this reaction was isolated and analysed by the ^1^H- and ^13^C-NMR, which revealed that the intermediate **3h** formed is 4-hydroxy-3-((naphthalen-1-ylthio)methyl)-4-(2-nitrophenyl)butan-2-one and it is obtained as mixture of diastereomers. When it was subjected to crystallization, the major diastereomer (with the relative configuration 7S* and 8S*) was crystallized out. The structure of the major diastereomer was confirmed by single crystal X-ray diffraction analysis (Figure 2). 

Hence it is quite clear that the intermediate **3h** obtained from the reaction of MBH alcohol and thiol is a conjugate adduct, which is different from the product from the reaction of MBH acetate and thiol [22,23,24,25]. However, our main aim was the synthesis of 2-methyl-3-(phenylthiomethyl)quinoline derivatives **4a**–**r** in a one-pot operation, therefore without further isolation of other intermediates **3a**–**r** (tetrahydrofuran solvent was simply removed under reduced pressure) we carried out the next step to obtain the 2-methyl-3-(phenylthiomethyl)quinoline derivatives **4a**–**r**.

The results presented in Table 1 reveal that thiophenol, thionaphthol and thiophenols containing alkyl, methoxy and halo substituents in the phenyl ring smoothly furnished the corresponding alkyl and arylthiomethylquinolines **4a**–**r** in 64–75% yields. There was no significant effect of the nature of the substituents on the yields of the products. All the products were fully characterized by ^1^H- and ^13^C-NMR as well as LR and HRMS. 

**Table 1:** One-pot synthesis of 2-methyl-3-(phenylthiomethyl)quinolines and their derivatives from Morita-Baylis-Hillman adducts.

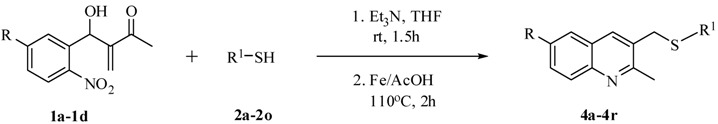						
Entry	MBH adduct 1	R	R ^1^	Thiol 2	Product 4	Yield(%) ^a,b^
1	**1a**	H	C_6_H_5_	**2a**	**4a**	66
2	**1a**	H	4-OMe-C_6_H_4_	**2b**	**4b**	69
3	**1a**	H	4-Br-C_6_H_4_	**2c**	**4c**	70
4	**1a**	H	4-Cl-C_6_H_4_	**2d**	**4d**	68
5	**1a**	H	4-F-C_6_H_4_	**2e**	**4e**	70
6	**1a**	H	4-Me-C_6_H_4_	**2f**	**4f**	67
7	**1a**	H	4-Et-C_6_H_4_	**2g**	**4g**	65
8	**1a**	H	1-Naphthyl	**2h**	**4h**	72
9	**1a**	H	Propyl	**2i**	**4i**	69
10	**1a**	H	Hexyl	**2j**	**4j**	65
11	**1a**	H	Isopropyl	**2k**	**4k**	65
12	**1a**	H	2-Naphthyl	**2l**	**4l**	75
13	**1a**	H	3,5-Me_2_-C_6_H_3_	**2m**	**4m**	64
14	**1a**	H	2-OMe-C_6_H_5_	**2n**	**4n**	65
15	**1a**	H	4-Isopropyl-C_6_H_4_	**2o**	**4o**	65
16	**1b**	Cl	C_6_H_5_	**2a**	**4p**	72
17	**1c**	F	C_6_H_5_	**2a**	**4q**	68
18	**1d**	Br	C_6_H_5_	**2a**	**4r**	66

^a^ All the reactions were carried out on 3 mmol scale; ^b^ Isolated yields were based on the MBH adducts.

**Scheme 2:**
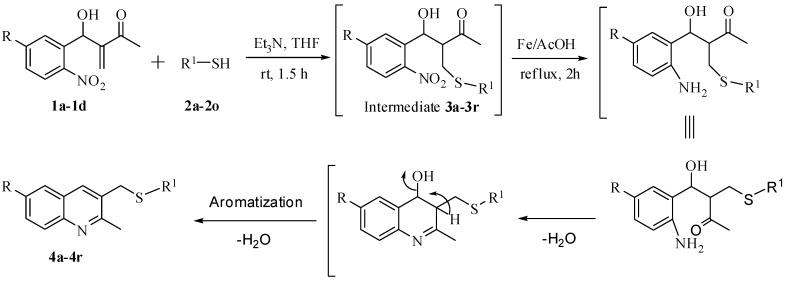
A Plausible mechanism for the formation of 2-methyl-3-(phenylthiomethyl) quinoline derivatives.

A plausible mechanism for the formation of the 2-methyl-3-(phenylthiomethyl)quinoline derivatives from the corresponding MBH adducts via reductive cyclization in the presence of Fe/AcOH is presented in Scheme 2.

**Table 2:** Synthesis of 2-methyl-3-(phenylsulfonylmethyl)quinoline derivatives.

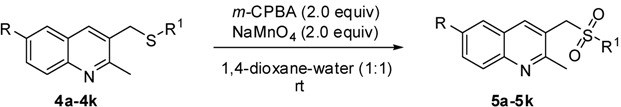						
Entry	Substrate 4	R	R ^1^	Time (min)	Product 5	Yield(%) ^a,b^
1	**4a**	H	C_6_H_5_	20	**5a**	85
2	**4b**	H	4-OMe-C_6_H_4_	20	**5b**	89
3	**4c**	H	4-Br-C_6_H_4_	40	**5c**	81
4	**4d**	H	4-Cl-C_6_H_4_	40	**5d**	80
5	**4e**	H	4-F-C_6_H_4_	35	**5e**	82
6	**4f**	H	4-Me-C_6_H_4_	20	**5f**	87
7	**4g**	H	4-Et-C_6_H_4_	20	**5g**	86
8	**4h**	H	1-Naphthyl	20	**5h**	87
9	**4i**	H	Propyl	20	**5i**	77
10	**4j**	H	Hexyl	20	**5j**	75
11	**4k**	H	Isopropyl	30	**5k**	78

^a^ All the reactions were carried out on 2 mmol scale; ^b^ Isolated yields.

Owing to the well known bioactivity of sulfones [33], the derived alkyl and arylthiomethylquinolines **4a**–**k** were oxidized to the corresponding sulfones **5a**–**k** in 75–89% yields (Table 2), following a reported procedure [34]. Here too, the substituents didn’t have any significant effect on the yields of the products. As before, all these products were also fully characterized by ^1^H- and ^13^C-NMR and by LR and HRMS data.

## 3. Experimental

### 3.1. General

All the reactions were performed in oven (130 °C) dried glassware under an inert atmosphere of nitrogen unless otherwise specified. Solvents for extraction and chromatography were distilled before use. All the chemicals used in this study were of commercial grade and used after distillation. Analytical thin layer chromatography was performed with E. Merck silica gel 60 F254 aluminum plates. All purifications were carried out by flash chromatography using 230–400 mesh silica gel. ^1^H and ^13^C-NMR were recorded on a Bruker Avance EX 400 FT NMR (Taipei, Taiwan). Chemical shifts were reported in parts per million (δ) using TMS as internal standard and coupling constants were expressed in Hertz. Mass spectra were obtained on a JOEL SX-102A spectrometer (Taipei, Taiwan) at an ionization potential of 70 eV and data are reported as mass/charge (*m/z*) with the percent relative abundance. High-resolution mass spectra (HRMS) were acquired with a Finnigan MAT-95XL spectrometer (Taipei, Taiwan).

### 3.2. General Procedure for the Synthesis of Compounds ***4a–r***

To a stirred solution of MBH adduct **1a** (3.0 mmol, 1 equiv) and benzenethiol **2a** (1.2 equiv) in THF (15 mL), triethylamine (1.5 equiv) was added dropwise, and the reaction mixture was allowed to continue at room temperature for 1.5 h. The progress of the reaction was monitored by TLC. After completion of the reaction, the solvent was removed under reduced pressure. The residue was diluted with AcOH (15 mL) and Fe powder (6.0 equiv) at room temperature was added. Then the reaction mixture was heated at 110 °C for 2 h and was cooled to room temperature. AcOH was removed under reduced pressure and the residue was diluted with EtOAc (30 mL). The resulting mixture was filtered to remove any iron impurities. Iron residue was washed twice with EtOAc (30 mL). Filtrate and washings were combined and dried over anhydrous Na_2_SO_4_. Solvent was evaporated and the residue, thus obtained was purified by column chromatography to provide the desired product **4a** as yellow solid in 66% isolated yield.

*2-Methyl-3-(phenylthiomethyl)quinoline* (**4a**). Yellow solid, m.p. 83–85 °C. ^1^H-NMR (DMSO-*d_6_*) *δ* 8.03 (s, 1H), 7.89 (d, *J* = 8.3 Hz, 1H), 7.78 (d, *J* = 7.8 Hz, 1H), 7.68–7.64 (m, 1H), 7.50–7.47 (m, 1H), 7.38–7.37 (m, 2H), 7.31–7.28 (m, 2H), 7.22–7.19 (m, 1H), 4.42 (s, 2H), 2.75 (s, 3H). ^13^C-NMR (CDCl_3_) *δ* 158.3, 147.1, 136.1, 135.4, 131.4, 129.4, 129.3, 129.2, 128.4, 127.4, 127.3, 127.0, 126.1, 37.6, 23.3. MS (*m/z*) (relative intensity): 265 (M^+^, 100), 156 (91). HRMS calcd. for C_17_H_15_NS (M^+^) 265.0920, found 265.0931. 

*3-((4-Methoxyphenylthio)methyl)-2-methyl* *quinoline* (**4b**). Yellow solid, m.p. 65–67 °C. ^1^H-NMR (CDCl_3_) *δ* 7.98 (d, *J* = 8.4 Hz, 1H), 7.61–7.57 (m, 1H), 7.55 (d, *J* = 8.1 Hz, 1H), 7.47 (s, 1H), 7.40–7.36 (m, 1H), 7.18 (d, *J* = 8.5 Hz, 2H), 6.72 (d, *J* = 8.5 Hz, 2H), 4.02 (s, 2H), 3.69 (s, 3H), 2.76 (s, 3H). ^13^C-NMR (CDCl_3_) *δ* 159.7, 158.2, 146.8, 135.9, 135.2, 129.6, 129.1, 128.3, 127.1, 126.8, 125.8, 125.0, 114.5, 55.3, 39.3, 23.1. MS (*m/z*) (relative intensity): 295 (M^+^, 8), 157 (6), 156 (100), 149 (16). HRMS calcd. for C_18_H_17_NOS (M^+^) 295.1025, found 295.1032. 

*3-((4-Bromophenylthio)methyl)-2-methylquinoline* (**4c**). Yellow solid, m.p. 118–120 °C. ^1^H-NMR (DMSO-*d_6_*) *δ* 8.05 (s, 1H), 7.89 (d, *J* = 8.3 Hz, 1H), 7.80 (d, *J* = 8.0 Hz, 1H), 7.69–7.65 (m, 1H), 7.51–7.46 (m, 3H), 7.32 (d, *J* = 8.4 Hz, 2H), 4.43 (s, 2H), 2.75 (s, 3H). ^13^C-NMR (100 MHz, CDCl_3_) *δ* 158.2, 147.2, 136.1, 134.6, 132.9, 132.3, 129.6, 128.8, 128.6, 127.4, 127.0, 126.2, 121.5, 37.7, 23.3. MS (*m/z*) (relative intensity): 345 (M^+^+2, 6), 343 (M^+^, 6), 157 (20), 156 (100), 129 (8), 115 (11), 69 (11). HRMS calcd for C_17_H_14_^79^BrNS (M^+^) 343.0025 found 343.0031; HRMS calcd. for C_17_H_14_^81^BrNS (M^+^) 345.0004, found 345.0016. 

*3-((4-Chlorophenylthio)methyl)-2-methylquinoline* (**4d**). Red solid, m.p. 98–100 °C. ^1^H-NMR (DMSO-*d_6_*) *δ* 8.04 (s, 1H), 7.89 (d, *J* = 8.4 Hz, 1H), 7.80 (d, *J* = 8.0 Hz, 1H), 7.69–7.65 (m, 1H), 7.51–7.47 (m, 1H) 7.39 (d, *J* = 8.6 Hz, 2H), 7.35 (d, *J* = 8.6 Hz, 2H), 4.43 (s, 2H), 2.75 (s, 3H). ^13^C-NMR (CDCl_3_) *δ* 158.3, 147.2, 136.1, 133.8, 133.6, 132.9, 129.6, 129.3, 128.9, 128.5, 127.4, 127.0, 126.2, 38.0, 23.3. MS (*m/z*) (relative intensity): 299 (M^+^, 9), 157 (16), 156 (100), 129 (10), 115 (11). HRMS calcd for C_17_H_14_^35^ClNS (M^+^) 299.0530 found 299.0540; HRMS calcd. for C_17_H_14_^37^ClNS (M^+^) 301.0500, found 301.0515. 

*3-((4-Fluorophenylthio)methyl)-2-methylquinoline* (**4e**). Yellow solid, m.p. 106–108 °C. ^1^H-NMR (DMSO-*d_6_*) *δ* 7.92 (s, 1H), 7.88 (d, *J* = 8.4 Hz, 1H), 7.77 (d, *J* = 8.0 Hz, 1H), 7.68–7.64 (m, 1H), 7.50–7.46 (m, 1H), 7.42–7.39 (m, 2H), 7.15–7.11 (m, 2H), 4.37 (s, 2H), 2.74 (s, 3H). ^13^C-NMR (CDCl_3_) *δ* 162.6 (d, *J* = 247.0 Hz), 158.2, 147.1, 136.0, 134.8 (d, *J* = 8.0 Hz), 129.9 (d, *J* = 3.0 Hz), 129.4, 129.1, 128.5, 127.2, 126.9, 126.1, 116.2 (d, *J* = 22.0 Hz), 38.9, 23.2. MS (*m/z*) (relative intensity): 283 (M^+^, 20), 157 (22), 156 (100), 129 (15), 115(16). HRMS calcd. for C_17_H_14_FNS (M^+^) 283.0826, found 283.0832. 

*2-Methyl-3-(p-tolylthiomethyl)quinoline* (**4f**). Yellow solid, m.p. 90–92 °C. ^1^H-NMR (CDCl_3_) *δ* 7.99 (d, *J* = 8.4 Hz, 1H), 7.70 (s, 1H), 7.66–7.62 (m, 2H), 7.45–7.42 (m, 1H), 7.20 (d, *J* = 7.8 Hz, 2H), 7.05 (d, *J* = 7.8 Hz, 2H), 4.17 (s, 2H), 2.81 (s, 3H), 2.30 (s, 3H). ^13^C-NMR (CDCl_3_) *δ* 158.4, 147.1, 137.6, 136.0, 132.1, 131.5, 129.9, 129.5, 129.3, 128.5, 127.3, 127.0, 126.0, 38.3, 23.3, 21.2. MS (*m/z*) (relative intensity): 279 (M^+^, 13), 157 (10), 156 (100), 129 (8), 115 (7). HRMS calcd. for C_18_H_17_NS (M^+^) 279.1076, found 279.1083. 

*3-((4-Ethylphenylthio)methyl)-2-methylquinoline* (**4g**). Brown liquid. ^1^H-NMR (CDCl_3_) *δ* 8.00 (d, *J* = 8.4 Hz, 1H), 7.63 (s, 1H), 7.61–7.57 (m, 2H), 7.42–7.38 (m, 1H), 7.20 (d, *J* = 8.1 Hz, 2H), 7.05 (d, *J* = 8.1 Hz, 2H), 4.12 (s, 2H), 2.79 (s, 3H), 2.57 (q, *J* = 7.6 Hz, 2H), 1.18 (t, *J* = 7.6 Hz, 3H). ^13^C-NMR (CDCl_3_) *δ* 158.3, 147.0, 143.8, 135.9, 132.1, 131.7, 129.4, 129.2, 128.6, 128.3, 127.2, 126.9, 125.9, 38.1, 28.5, 23.2, 15.6. MS (*m/z*) (relative intensity): 293 (M^+^, 17), 157 (12), 156 (100), 149 (24), 129 (8). HRMS calcd. for C_19_H_19_NS (M^+^) 293.1233, found 293.1242. 

*2-Methyl-3-((naphthalen-1-ylthio)methyl)quinoline* (**4h**). Yellow solid, m.p. 85–87 °C. ^1^H-NMR (CDCl_3_) *δ* 8.44 (d, *J* = 8.2 Hz, 1H), 7.98 (d, *J* = 8.4 Hz, 1H), 7.85 (d, *J* = 7.7 Hz, 1H), 7.77 (d, *J* = 8.2 Hz, 1H), 7.64–7.60 (m, 1H), 7.55–7.48 (m, 3H), 7.44–7.37 (m, 3H), 7.29 (d, *J* = 7.4 Hz, 1H), 4.23 (s, 2H), 2.85 (s, 3H). ^13^C-NMR (CDCl_3_) *δ* 158.3, 147.1, 136.1, 134.1, 133.7, 132.2, 131.5, 129.3, 129.2, 128.8, 128.7, 128.4, 127.2, 127.0, 126.9, 126.4, 126.0, 125.6, 125.2, 37.8, 23.3. MS (*m/z*) (relative intensity): 315 (M^+^, 100), 156 (70). HRMS calcd. for C_21_H_17_NS (M^+^) 315.1076, found 315.1087. 

*2-Methyl-3-(propylthiomethyl)quinoline* (**4i**). Yellow solid, m.p. 44–46 °C. ^1^H-NMR (CDCl_3_) *δ* 8.00 (d, *J* = 8.4 Hz, 1H), 7.90 (s, 1H), 7.74 (d, *J* = 8.0 Hz, 1H), 7.66–7.63 (m, 1H), 7.49–7.45 (m, 1H), 3.84 (s, 2H), 2.81 (s, 3H), 2.45 (t, *J* = 7.2 Hz, 2H), 1.66–1.57(m, 2H), 0.97 (t, *J* = 7.3 Hz, 3H). ^13^C-NMR (CDCl_3_) *δ* 158.6, 147.0, 135.6, 130.1, 129.2, 128.5, 127.2, 127.0, 126.1, 34.2, 34.0, 23.2, 22.7, 13.6. MS (*m/z*) (relative intensity): 231 (M^+^, 100), 156 (51). HRMS calcd. for C_14_H_17_NS (M^+^) 231.1076, found 231.1086. 

*3-(Hexylthiomethyl)-2-methylquinoline* (**4j**). Yellow liquid. ^1^H-NMR (CDCl_3_) *δ* 8.00 (d, *J* = 8.4 Hz, 1H), 7.89 (s, 1H), 7.74 (d, *J* = 8.0 Hz, 1H), 7.66–7.62 (m, 1H), 7.49–7.45 (m, 1H), 3.84 (s, 2H), 2.81 (s, 3H), 2.46 (t, *J* = 7.4 Hz, 2H), 1.60–1.54 (m, 2H), 1.37–1.22 (m, 6H), 0.86 (t, *J* = 6.7 Hz, 3H). ^13^C-NMR (CDCl_3_) *δ* 158.4, 146.8, 135.3, 129.9, 129.0, 128.3, 127.0, 126.8, 125.8, 34.1, 31.8, 31.3, 29.1, 28.5, 23.0, 22.4, 13.9. MS (*m/z*) (relative intensity): 273 (M^+^, 100), 189 (7), 156 (62). HRMS calcd. for C_17_H_23_NS (M^+^) 273.1546, found 273.1554. 

*3-(Isopropylthiomethyl)-2-methylquinoline* (**4k**). Yellow liquid. ^1^H-NMR (DMSO-*d_6_*) *δ* 8.13 (s, 1H), 7.91–7.88 (m, 2H), 7.69–7.65 (m, 1H), 7.53–7.50 (m, 1H), 3.95 (s, 2H), 2.88–2.81 (m, 1H), 2.73 (s, 3H), 1.24 (d, *J* = 6.8 Hz, 6H). ^13^C-NMR (CDCl_3_) *δ* 158.4, 146.7, 135.4, 130.1, 129.0, 128.2, 127.0, 126.9, 125.9, 34.9, 32.7, 23.1, 22.9. MS (*m/z*) (relative intensity): 231 (M^+^, 100), 156 (46). HRMS calcd. for C_14_H_17_NS (M^+^) 231.1076, found 231.1086. 

*2-Methyl-3-((naphthalen-2-ylthio)methylquinoline* (**4l**). Yellow solid, m.p. 115–117 °C. ^1^H-NMR (CDCl_3_) *δ* 8.00 (d, *J* = 8.4 Hz, 1H), 7.79–7.77 (m, 2H), 7.75–7.73 (m, 2H), 7.68–7.57 (m, 3H), 7.47–7.39 (m, 4H), 4.33 (s, 2H), 2.86 (s, 3H). ^13^C-NMR (CDCl_3_) *δ* 158.3, 147.1, 136.1, 133.8, 133.0, 132.3, 129.4, 129.3, 129.1, 128.7, 128.5, 128.4, 127.8, 127.4, 127.3, 127.0, 126.7, 126.2, 126.0, 37.4, 23.3. MS (*m/z*) (relative intensity): 315 (M^+^, 21), 157 (12), 156 (100), 129 (8), 115 (18). HRMS calcd. for C_21_H_17_NS (M^+^) 315.1076, found 315.1083. 

*3-((3*,*5-Dimethylphenylthio)methyl)-2-methylquinoline* (**4m**). Yellow solid, m.p. 108–110 °C. ^1^H-NMR (CDCl_3_) *δ* 8.00 (d, *J* = 8.7 Hz, 1H), 7.75 (s, 1H), 7.66–7.62 (m, 2H), 7.46–7.42 (m, 1H), 6.92 (s, 2H), 6.85 (s, 1H), 4.20 (s, 2H), 2.83 (s, 3H), 2.22 (s, 6H). ^13^C-NMR (DMSO-*d_6_*) *δ* 158.1, 146.3, 138.1, 135.6, 134.7, 129.2, 129.1, 128.0, 127.8, 127.2, 126.9, 126.4, 125.8, 35.0, 22.7, 20.6. MS (*m/z*) (relative intensity): 293 (M^+^, 32), 157 (11), 156 (100), 151 (15). HRMS calcd. for C_19_H_19_NS (M^+^) 293.1233, found 293.1241. 

*3-((2-Methoxyphenylthio)methyl)-2-methylquinoline* (**4n**). Yellow solid, m.p. 118–120 °C. ^1^H-NMR (CDCl_3_) *δ* 7.98 (d, *J* = 8.3 Hz, 1H), 7.71 (s, 1H), 7.63–7.59 (m, 2H), 7.43–7.39 (m, 1H), 7.23–7.19 (m, 2H), 6.84–6.80 (m, 2H), 4.18 (s, 2H), 3.81 (s, 3H), 2.84 (s, 3H). ^13^C-NMR (CDCl_3_) *δ* 158.7, 158.6, 147.1, 136.0, 132.9, 129.5, 129.2, 129.0, 128.4, 127.3, 127.1, 125.9, 123.0, 121.2, 110.8, 55.9, 35.8, 23.2. MS (*m/z*) (relative intensity): 295 (M^+^, 12), 157 (7), 156 (100). HRMS calcd. for C_18_H_17_NOS (M^+^) 295.1025, found 295.1034. 

*3-((4-Isopropylphenylthio)methyl)-2-methylquinoline* (**4o**). Red solid, m.p. 44–46°C. ^1^H-NMR (DMSO-*d_6_*) *δ* 7.96 (s, 1H), 7.89 (d, *J* = 8.4 Hz, 1H), 7.76 (d, *J* = 8.0 Hz, 1H), 7.68–7.64 (m, 1H), 7.50–7.46 (m, 1H), 7.28 (d, *J* = 8.0 Hz, 2H), 7.16 (d, *J* = 8.1 Hz, 2H), 4.36 (s, 2H), 2.86–2.79 (m, 1H), 2.74 (s, 3H), 1.14 (d, *J* = 6.8 Hz, 6H). ^13^C-NMR (DMSO-*d_6_*) *δ* 158.1, 147.1, 146.2, 135.4, 131.8, 130.2, 129.3, 129.1, 127.8, 127.2, 127.0, 126.4, 125.8, 35.8, 32.9, 23.6, 22.7. MS (*m/z*) (relative intensity): 307 (M^+^, 65), 156 (100). HRMS calcd. for C_20_H_21_NS (M^+^) 307.1389, found 307.1403. 

*6-Chloro-2-methyl-3-(phenylthiomethyl)quinoline* (**4p**). Green solid, m.p. 74–76 °C. ^1^H-NMR (DMSO-*d_6_*) *δ* 8.00 (s, 1H), 7.91–7.88 (m, 2H), 7.65 (d, *J* = 8.9 Hz, 1H), 7.37–7.35 (m, 2H), 7.31–7.27 (m, 2H), 7.22–7.19 (m, 1H), 4.40 (s, 2H), 2.74 (s, 3H). ^13^C-NMR (CDCl_3_) *δ* 158.7, 145.5, 135.1, 135.0, 131.8, 131.7, 130.4, 130.3, 130.2, 129.2, 127.7, 127.5, 125.9, 37.7, 23.3. MS (*m/z*) (relative intensity): 299 (M^+^, 10), 192 (23), 190 (100), 155 (10). HRMS calcd. for C_17_H_14_^35^ClNS (M^+^) 299.0530, found 299.0540; HRMS calcd. for C_17_H_14_^37^ClNS (M^+^) 301.0500, found 301.0515. 

*6-Fluoro-2-methyl-3-(phenylthiomethyl)quinoline* (**4q**). Yellow solid, m.p. 44–46 °C. ^1^H-NMR (DMSO-*d_6_*) *δ* 8.02 (s, 1H), 7.95 (dd, *J* = 9.0, 5.4 Hz, 1H), 7.62–7.53 (m, 2H), 7.37–7.35 (m, 2H), 7.31-7.27 (m, 2H), 7.23–7.19 (m, 1H), 4.40 (s, 2H), 2.73 (s, 3H). ^13^C-NMR (DMSO-*d_6_*) *δ* 159.3 (d, *J* = 242.0 Hz), 157.6, 143.4, 135.0, 134.9 (d, *J* = 5.0 Hz), 130.6 (d, *J* = 9.0 Hz), 130.2, 129.5, 129.0, 127.0 (d, *J* = 10.0 Hz), 126.5, 118.9 (d, *J* = 25.0 Hz), 110.3 (d, *J* = 21.0 Hz), 35.0, 22.6. MS (*m/z*) (relative intensity): 283 (M^+^, 15), 175 (7), 174 (100), 147 (6). HRMS calcd. for C_17_H_14_FNS (M^+^) 283.0826, found 283.0830. 

*6-Bromo-2-methyl-3-henylthiomethyl)quinoline* (**4r**). Yellow solid, m.p. 78–80 °C. ^1^H-NMR (DMSO-*d_6_*) *δ* 8.08 (d, *J* = 1.9 Hz, 1H), 8.01 (s, 1H), 7.83 (d, *J* = 8.9 Hz, 1H), 7.77 (dd, *J* = 8.9, 1.8 Hz, 1H), 7.37–7.35 (m, 2H), 7.31–7.28 (m, 2H), 7.23–7.19 (m, 1H), 4.41 (s, 2H), 2.74 (s, 3H). ^13^C-NMR (DMSO-*d_6_*) *δ* 158.9, 144.8, 134.8, 134.5, 132.1, 130.4, 130.0, 129.6, 129.1, 129.0, 127.7, 126.5, 118.6, 35.0, 22.7. MS (*m/z*) (relative intensity): 345 (M^+^+2, 9), 343 (M^+^, 8), 236 (97), 234 (100), 156 (24), 155 (36), 149 (18), 114 (19). HRMS calcd. for C_17_H_14_^79^BrNS (M^+^) 343.0025, found 343.0024. HRMS calcd. for C_17_H_14_^81^BrNS (M^+^) 345.004, found 345.0015. 

### 3.3. General Procedure for the Synthesis of Compounds ***5a–k***

To a stirred solution of sulfide **4a** (2.0 mmol, 1 equiv) in 1:1 1,4-dioxane/H_2_O (12 mL) was added *m-*CPBA (2.0 equiv.) followed by sodium permanganate (2.0 equiv.) at room temperature (25 °C), and the reaction mixture was stirred at same temperature for 20 min. After completion of the reaction as monitored by TLC, the reaction mixture was extracted with EtOAc (30 mL), then organic layer was washed with saturated NaHCO_3_, water, followed by brine solution and dried over Na_2_SO_4_. Solvent was evaporated and obtained crude product was purified by column chromatography to provide the desired sulfone **5a** as yellow solid in 85 % isolated yield. 

*2-Methyl-3-(phenylsulfonylmethyl)quinoline* (**5a**). Yellow solid, m.p. 148–150 °C. ^1^H-NMR (CDCl_3_) *δ* 7.99 (d, *J* = 8.4 Hz, 1H), 7.89 (s, 1H), 7.73–7.63 (m, 5H), 7.52–7.46 (m, 3H), 4.53 (s, 2H), 2.48 (s, 3H). ^13^C-NMR (CDCl_3_) *δ* 158.3, 147.3, 139.6, 138.2, 134.3, 130.4, 129.4, 128.7, 128.6, 127.6, 126.6, 126.5, 120.8, 59.7, 23.2. MS (*m/z*) (relative intensity): 297 (M^+^, 3), 157 (31), 156 (100), 129 (16), 115 (19). HRMS calcd. for C_17_H_15_NO_2_S (M^+^) 297.0818, found 297.0830.

*3-((4-Methoxyphenylsulfonyl)methyl)-2-methylquinoline* (**5b**). White solid, m.p. 178–180 °C. ^1^H-NMR (DMSO-*d_6_*) *δ* 7.99 (s, 1H), 7.91 (d, *J* = 8.3 Hz, 1H), 7.84 (d, *J* = 8.0 Hz, 1H), 7.74–7.71 (m, 1H), 7.65 (d, *J* = 8.7 Hz, 2H), 7.56–7.52 (m, 1H), 7.12 (d, *J* = 8.7 Hz, 2H), 4.85 (s, 2H), 3.84 (s, 3H), 2.54 (s, 3H). ^13^C-NMR (CDCl_3_) *δ* 164.2, 158.4, 147.6, 139.6, 130.9, 130.4, 129.6, 128.6, 127.7, 126.7, 126.4, 121.3, 114.6, 59.8, 55.8, 23.3. MS (*m/z*) (relative intensity): 327 (M^+^, 27), 157 (89), 156 (100), 129 (30), 128 (12), 116 (20), 115 (63), 89 (13). HRMS calcd. for C_18_H_17_NO_3_S (M^+^) 327.0924, found 327.0936. 

*3-((4-Bromophenylsulfonyl)methyl)-2-methylquinoline* (**5c**). White solid, m.p. 213–215 °C. ^1^H-NMR (CDCl_3_) *δ* 8.00 (d, *J* = 8.6 Hz, 1H), 7.91 (s, 1H), 7.74–7.71 (m, 2H), 7.62 (d, *J* = 8.4 Hz, 2H), 7.53–7.51 (m, 3H), 4.52 (s, 2H), 2.51 (s, 3H). ^13^C-NMR (CDCl_3_) *δ* 158.2, 147.8, 139.7, 137.2, 132.8, 130.6, 130.3, 129.8, 128.7, 127.6, 126.7, 126.6, 120.5, 59.7, 23.4. MS (*m/z*) (relative intensity): 376 (M^+^+2, 3), 374 (M^+^, 3), 157 (89), 156 (100), 154 (11), 129 (30), 115 (62), 89 (13). HRMS calcd. for C_17_H_14_^79^Br NO_2_S (M^+^) 374.9923, found 374.9937; HRMS calcd. for C_17_H_14_^81^BrNO_2_S (M^+^) 376.9903, found 376.9922. 

*3-((4-Chlorophenylsulfonyl)methyl)-2-methyl quinoline* (**5d**). White solid, m.p. 190–192 °C. ^1^H-NMR (CDCl_3_) *δ* 7.99 (d, *J* = 8.7 Hz, 1H), 7.91 (s, 1H), 7.74–7.71 (m, 2H), 7.59 (d, *J* = 8.5 Hz, 2H), 7.53–7.50 (m, 1H), 7.44 (d, *J* = 8.5 Hz, 2H), 4.53 (s, 2H), 2.50 (s, 3H). ^13^C-NMR (CDCl_3_) *δ* 158.2, 147.8, 141.2, 139.7, 136.6, 130.6, 130.2, 129.8, 128.7, 127.6, 126.7, 126.6, 120.5, 59.7, 23.4. HRMS calcd. for C_17_H_14_ClNO_2_S (M^+^) 331.0434, found 331.0437. 

*3-((4-Fluorophenylsulfonyl)methyl)-2-methyl quinoline* (**5e**). White solid, m.p. 183–185 °C. ^1^H-NMR (CDCl_3_) *δ* 7.99 (d, *J* = 8.5 Hz, 1H), 7.89 (s, 1H), 7.74–7.65 (m, 4H), 7.53–7.49 (m, 1H), 7.16–7.12 (m, 2H), 4.53 (s, 2H), 2.51 (s, 3H). ^13^C-NMR (DMSO-*d_6_*) *δ* 165.2 (d, *J* = 252.0 Hz), 158.7, 146.7, 139.4, 134.7 (d, *J* = 2.0 Hz), 131.4 (d, *J* = 10.0 Hz), 130.0, 127.9, 127.5, 126.1, 126.0, 121.2, 116.5 (d, *J* = 23.0 Hz), 58.0, 22.9. HRMS calcd. for C_17_H_14_FNO_2_S (M^+^) 315.0729, found 315.0732.

*2-Methyl-3-(tosylmethyl)quinoline* (**5f**). Yellow solid, m.p. 180–182 °C. ^1^H-NMR (DMSO-*d_6_*) *δ* 8.01 (s, 1H), 7.91 (d, *J* = 8.4 Hz, 1H), 7.84 (d, *J* = 8.0 Hz, 1H), 7.75–7.71 (m, 1H), 7.63 (d, *J* = 8.0 Hz, 2H), 7.56–7.52 (m, 1H), 7.42 (d, *J* = 8.0 Hz, 2H), 4.88 (s, 2H), 2.52 (s, 3H), 2.40 (s, 3H). ^13^C-NMR (CDCl_3_) *δ* 158.3, 147.7, 145.4, 139.6, 135.2, 130.4, 130.0, 128.7, 128.6, 127.6, 126.7, 126.4, 121.0, 59.7, 23.3, 21.7. HRMS calcd. for C_18_H_17_ NO_2_S (M^+^) 311.0980, found 311.0985.

*3-((4-Ethylphenylsulfonyl)methyl)-2-methyl quinoline* (**5g**). White solid, m.p. 160–162 °C. ^1^H-NMR (DMSO-*d_6_*) *δ* 7.97 (s, 1H), 7.91 (d, *J* = 8.3 Hz, 1H), 7.82 (d, *J* = 8.0 Hz, 1H), 7.75–7.71 (m, 1H), 7.66 (d, *J* = 7.8 Hz, 2H), 7.55–7.52 (m, 1H), 7.45 (d, *J* = 7.8 Hz, 2H), 4.88 (s, 2H), 2.70 (q, *J* = 7.5 Hz, 2H), 2.54 (s, 3H), 1.19 (t, *J* = 7.5 Hz, 3H). ^13^C-NMR (CDCl_3_) *δ* 158.4, 151.5, 147.6, 139.6, 135.3, 130.4, 128.9, 128.8, 128.5, 127.6, 126.6, 126.4, 121.0, 59.7, 29.0, 23.2, 15.2. HRMS calcd. for C_19_H_19_ NO_2_S (M^+^) 325.1136, found 325.1125.

*2-Methyl-3-((naphthalen-1-ylsulfonyl)methyl) quinoline* (**5h**). White solid, m.p. 158–160 °C. ^1^H-NMR (CDCl_3_) *δ* 8.80–8.77 (m, 1H), 8.12 (d, *J* = 8.2 Hz, 1H), 8.02–7.93 (m, 3H), 7.69–7.61 (m, 4H), 7.55–7.53 (m, 1H), 7.46–7.42 (m, 2H), 4.70 (s, 2H), 2.51 (s, 3H). ^13^C-NMR (CDCl_3_) *δ* 158.4, 147.6, 139.6, 135.8, 134.2, 133.2, 131.7, 130.4, 129.5, 129.3, 129.0, 128.5, 127.5, 127.3, 126.5, 126.4, 124.5, 124.0, 120.9, 59.3, 23.4. HRMS calcd. for C_21_H_17_NO_2_S (M^+^) 347.0980, found 347.0976.

*2-Methyl-3-(propylsulfonylmethyl)quinoline* (**5i**). White solid, m.p. 148–150 °C. ^1^H-NMR (CDCl_3_) *δ* 8.15 (s, 1H), 8.02 (d, *J* = 8.4 Hz, 1H), 7.79 (d, *J* = 8.1 Hz, 1H), 7.73–7.69 (m, 1H), 7.53–7.49 (m, 1H), 4.42 (s, 2H), 3.00–2.96 (m, 2H), 2.86 (s, 3H), 1.96–1.88 (m, 2H), 1.08 (t, *J* = 7.4 Hz, 3H). ^13^C-NMR (CDCl_3_) *δ* 158.4, 147.9, 139.4, 130.5, 128.7, 127.7, 126.8, 126.6, 120.3, 56.2, 54.3, 23.9, 15.8, 13.3. HRMS calcd. for C_14_H_17_NO_2_S (M^+^) 263.0980, found 263.0986. 

*3-(Hexylsulfonylmethyl)-2-methylquinoline* (**5j**). White solid, m.p. 127–129 °C. ^1^H-NMR (CDCl_3_) *δ* 8.15 (s, 1H), 8.02 (d, *J* = 8.4 Hz, 1H), 7.79 (d, *J* = 8.0 Hz, 1H), 7.73–7.69 (m, 1H), 7.53–7.49 (m, 1H), 4.42 (s, 2H), 3.01–2.97 (m, 2H), 2.86 (s, 3H), 1.91–1.84 (m, 2H), 1.46–1.39 (m, 2H), 1.33–1.25 (m, 4H), 0.86 (t, *J* = 6.8 Hz, 3H). ^13^C-NMR (CDCl_3_) *δ* 158.4, 147.9, 139.4, 130.5, 128.7, 127.7, 126.8, 126.6, 120.3, 56.1, 52.7, 31.3, 28.3, 24.0, 22.4, 22.0, 14.0. HRMS calcd. for C_17_H_23_NO_2_S (M^+^) 305.1449, found 305.1460. 

*3-(Isopropylsulfonylmethyl)-2-methylquinoline* (**5k**). White solid, m.p. 145–147 °C. ^1^H-NMR (CDCl_3_) *δ* 8.15 (s, 1H), 8.10 (d, *J* = 8.4 Hz, 1H), 7.78 (d, *J* = 8.1 Hz, 1H), 7.72–7.68 (m, 1H), 7.50 (s, 1H), 4.39 (s, 2H), 3.25–3.18 (m, 1H), 2.85 (s, 3H), 1.14 (d, *J* = 6.8 Hz, 6H). ^13^C-NMR (CDCl_3_) *δ* 158.7, 147.7, 139.6, 130.3, 128.6, 127.6, 126.7, 126.4, 119.8, 53.5, 52.1, 23.8, 15.7. HRMS calcd. for C_14_H_17_NO_2_S (M^+^) 263.0980, found 263.0988. 

*Crystallographic description of* **3h:** crystal size = 0.44 × 0.32 × 0.08 mm^3^; C_21_H_19_NO_4_S; *M_r_* = 381.43; Monoclinic; Space group P 2_1_/c; a = 32.290(4) Å, α = 90°, b = 7.4466(9) Å, β = 103.742(6)°, c = 15.7324(16) Å, γ = 90°, *V* = 3674.6(7) Å3; *Z* = 8; *ρ*_cal_ = 1.379 mg/m3; *μ* = 0.204 mm^−1^; F(000) = 1600; Theta range for data collection = 1.30 to 25.02°; Index ranges = −29 ≤ *h* ≤ 38, −7 ≤ *k* ≤ 8, −16 ≤ *l* ≤ 18; Reflections collected / Independent reflection = 13232/6139 [R(int) = 0.0734]; Completeness to theta = 25.02° 94.4%; Absorption correction = multi-scan; Max. and min. transmission = 0.9839 and 0.9158; Refinement method = Full-matrix least-squares on F2; Data / restraints / parameters = 6139/0/277; Goodness-of-fit on F2 = 1.183; Final R indices = [I>2sigma(I)] R1 = 0.2457, *w*R2 = 0.4913; R indices (all data) = R1 = 0.2811, *w*R2 = 0.5062; Largest diff. peak and hole = 1.222 and −0.713 e.Å−3.

## 4. Conclusions

In conclusion, we have successfully synthesized 2-methyl-3-(phenylthiomethyl)quinoline derivatives in good yields from MBH adducts via a convenient one-pot procedure. The 2-methyl-3-(phenylthiomethyl)quinoline reaction products of were transformed into the corresponding 2-methyl-3-(phenylsulfonylmethyl)quinoline derivatives in good yields. The C3-alkylthiomethylquinolines are very useful precursors for the synthesis of C3-alkylsulfonyl or C3-alkylsulfinyl containing quinoline derivatives.

## Supplementary Materials

Supplementary materials can be accessed at: http://www.mdpi.com/1420-3049/17/5/5081/s1.
