# Supplementary material for: A Convenient One-Pot Preparation of 2-Methyl-3-(phenylthio- methyl)quinolines from Morita-Baylis-Hillman Adducts and Their Oxidation to the Corresponding Sulfones
Source: Molecules. 2012 May 3;17(5):5081–94. doi: 10.3390/molecules17055081 (PMC6268867; doi:10.3390/molecules17055081)
Supplement: Supplementary file 1 [file molecules-17-05081-s001.pdf]

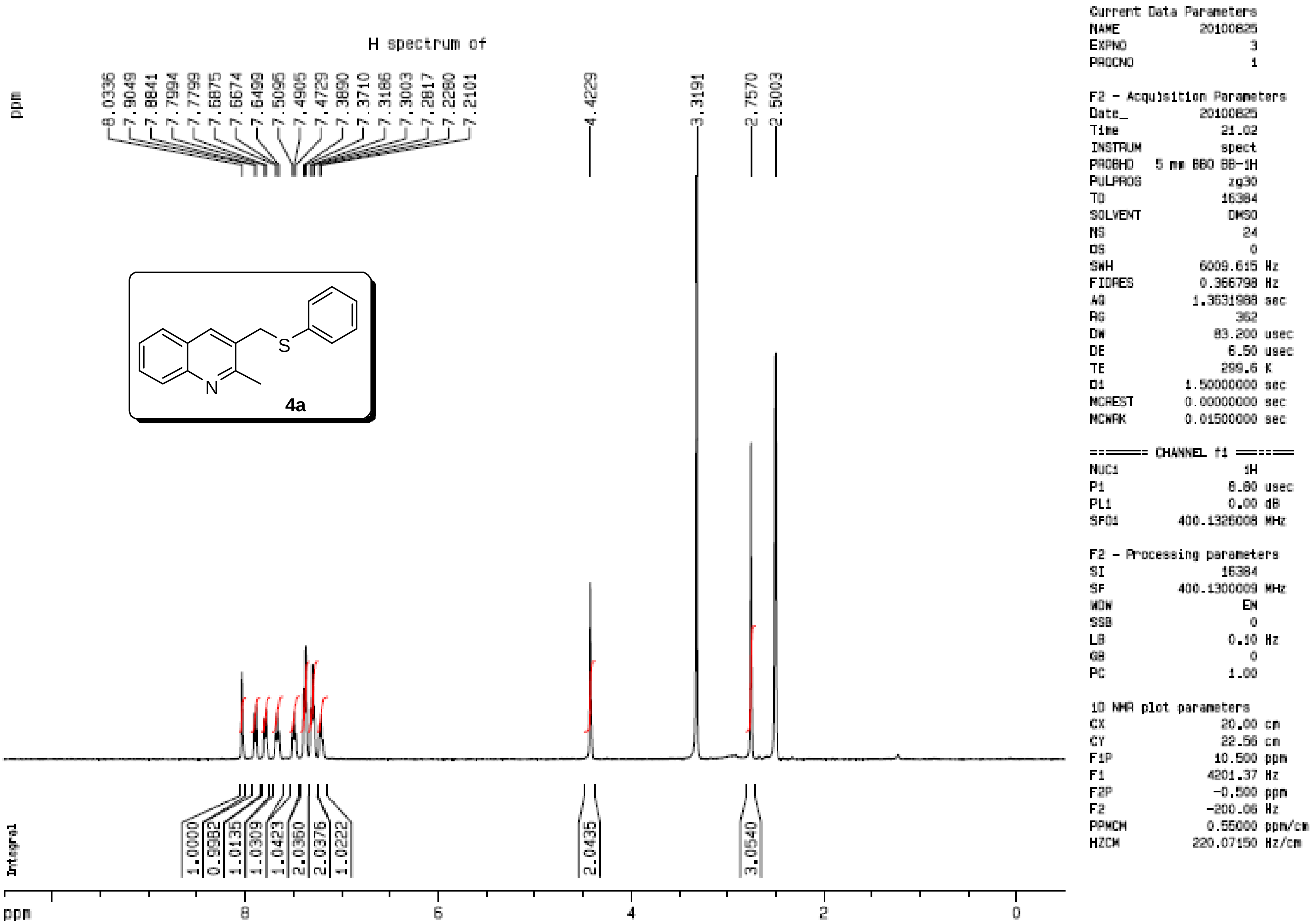
H spectrum of
8.0336
7.9049
7.8841
7.7994
7.7799
7.6875
7.6674
7.6499
7.5095
7.4905
7.4729
7.3890
7.3710
7.3186
7.3003
7.2817
7.2280
7.2101
4.4229
3.3191
2.7570
2.5003
S
N
4a
Integral
1.0000
0.9982
1.0135
1.0309
1.0423
2.0350
2.0376
1.0222
2.0435
3.0540
ppm
8
6
4
2
0
Current Data Parameters
NAME 20100825
EXPNO 3
PROCNO 1
F2 - Acquisition Parameters
Date_ 20100825
Time 21.02
INSTRUM spect
PROBHD 5 mm BBO BB-1H
PULPROG zg30
TD 16384
SOLVENT DMSO
NS 24
DS 0
SWH 6009.615 Hz
FIDRES 0.366798 Hz
AQ 1.3631988 sec
RG 362
DW 83.200 usec
DE 6.50 usec
TE 299.6 K
D1 1.50000000 sec
MCREST 0.00000000 sec
MCWRK 0.01500000 sec
======== CHANNEL f1 ========
NUC1 1H
P1 8.80 usec
PL1 0.00 dB
SFO1 400.1326008 MHz
F2 - Processing parameters
SI 16384
SF 400.1300009 MHz
WDW EM
SSB 0
LB 0.10 Hz
GB 0
PC 1.00
1D NMR plot parameters
CX 20.00 cm
CY 22.56 cm
F1P 10.500 ppm
F1 4201.37 Hz
F2P -0.500 ppm
F2 -200.06 Hz
PPMCM 0.55000 ppm/cm
HZCM 220.07150 Hz/cm

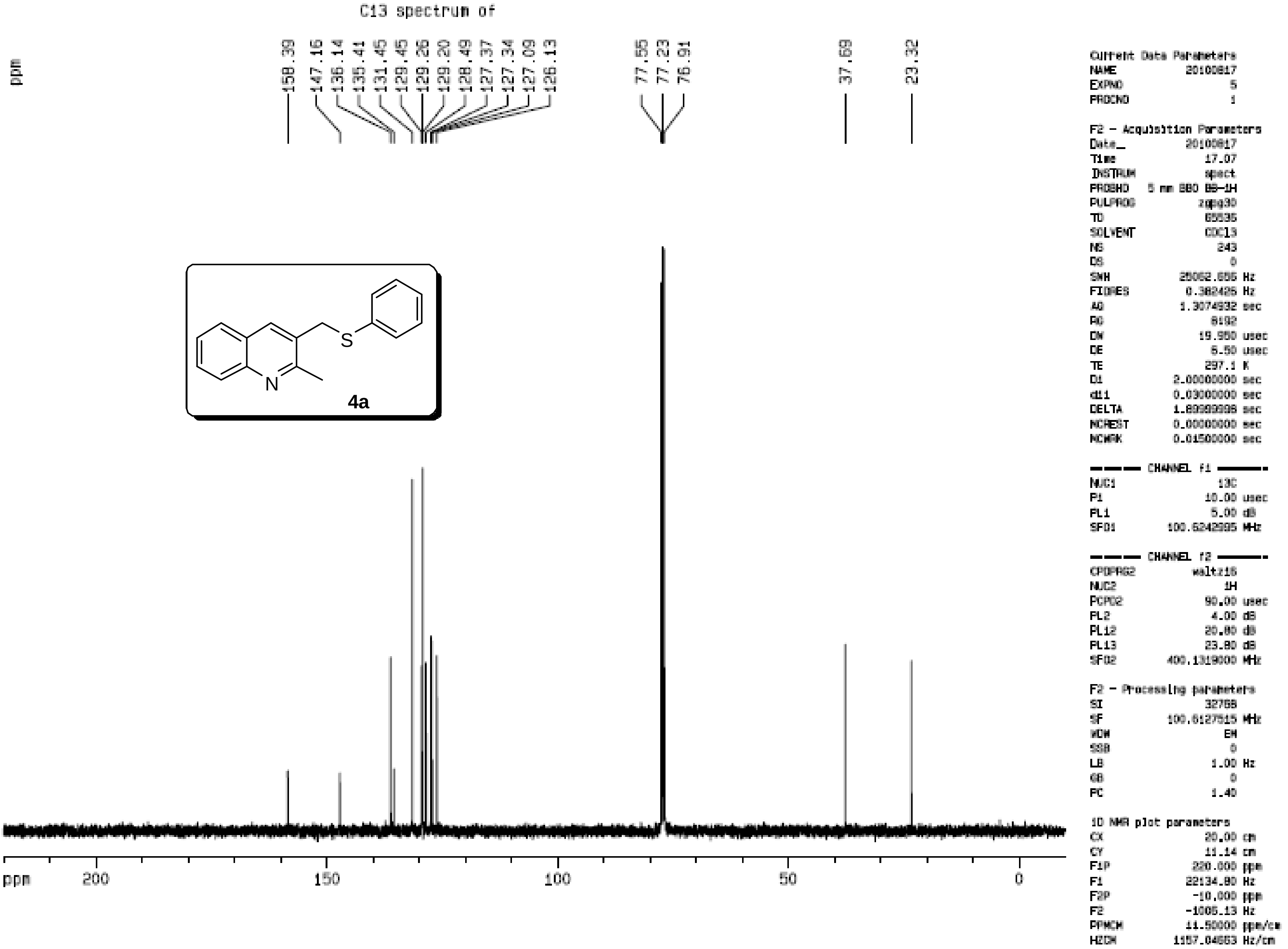
C13 spectrum of
158.39
147.16
136.14
135.41
131.45
129.45
129.26
129.20
128.49
127.37
127.34
127.09
126.13
77.55
77.23
76.91
37.69
23.32
S
N
4a
ppm
200
150
100
50
0
Current Data Parameters
NAME 20100817
EXPNO 5
PROCNO 1
F2 - Acquisition Parameters
Date_ 20100817
Time 17.07
INSTRUM spect
PROBHD 5 mm BBO BB-1H
PULPROG zgpg30
TD 65536
SOLVENT CDCl3
NS 243
DS 0
SWH 25062.656 Hz
FIDRES 0.382425 Hz
AQ 1.3074932 sec
RG 8192
DW 19.950 usec
DE 6.50 usec
TE 297.1 K
D1 2.00000000 sec
d11 0.03000000 sec
DELTA 1.89999998 sec
MCREST 0.00000000 sec
MCWRK 0.01500000 sec
CHANNEL f1
NUC1 13C
P1 10.00 usec
PL1 5.00 dB
SFO1 100.6242695 MHz
CHANNEL f2
CPDPRG2 waltz16
NUC2 1H
PCPD2 90.00 usec
PL2 4.00 dB
PL12 20.80 dB
PL13 23.80 dB
SFO2 400.1319000 MHz
F2 - Processing parameters
SI 32768
SF 100.6127515 MHz
WDW EM
SSB 0
LB 1.00 Hz
GB 0
PC 1.40
1D NMR plot parameters
CX 20.00 cm
CY 11.14 cm
F1P 220.000 ppm
F1 22134.80 Hz
F2P -10.000 ppm
F2 -1006.13 Hz
PPMCM 11.50000 ppm/cm
HZCM 1157.04663 Hz/cm

H spectrum of

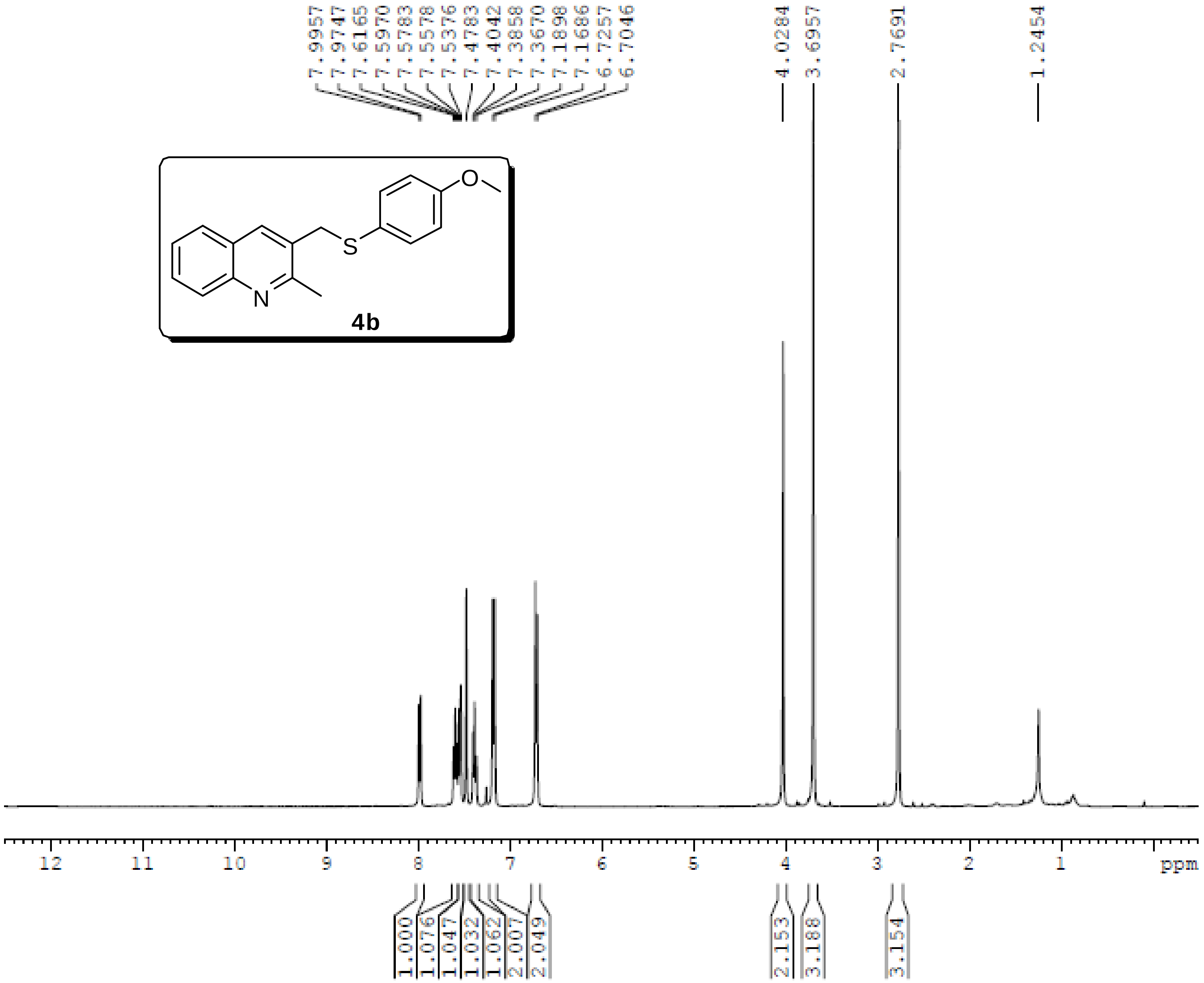

Current Data Parameters
NAME 20100725
EXPNO 2
PROCNO 1

F2 - Acquisition Parameters
Date_ 20100725
Time 23.17
INSTRUM spect
PROBHD 5 mm BBO BB-1H
PULPROG zg30
TD 16384
SOLVENT CDCl3
NS 16
DS 0
SWH 6009.615 Hz
FIDRES 0.366798 Hz
AQ 1.3632620 sec
RG 28.5
DW 83.200 usec
DE 6.50 usec
TE 298.4 K
D1 1.50000000 sec
MCREST 0 sec
MCWRK 0.01500000 sec

======== CHANNEL f1 ========
NUC1 1H
P1 14.50 usec
PL1 5.20 dB
SFO1 400.1326008 MHz

F2 - Processing parameters
SI 16384
SF 400.1300060 MHz
WDW EM
SSB 0
LB 0.10 Hz
GB 0
PC 1.00

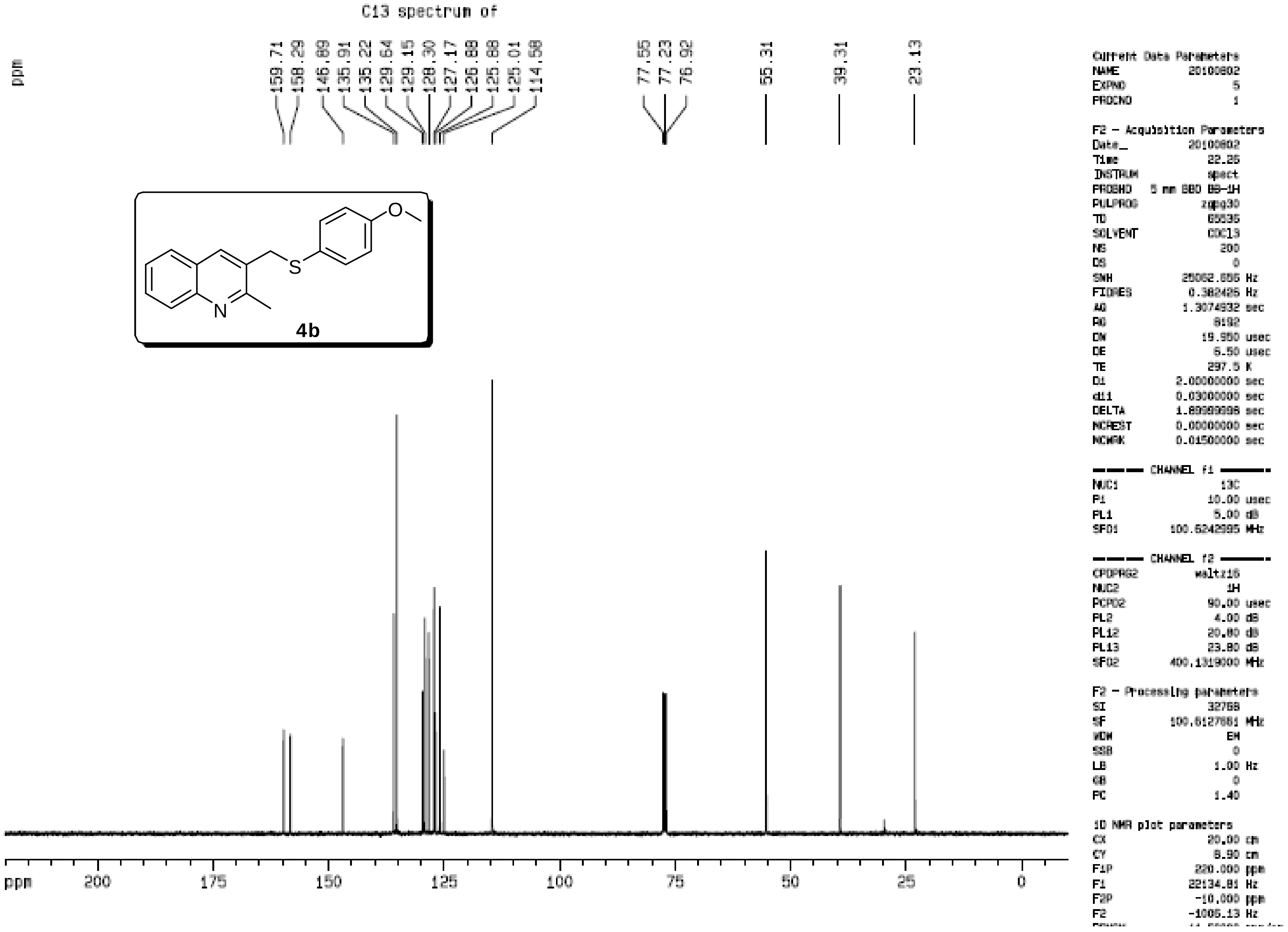
C13 spectrum of
ppm
159.71
158.29
146.89
135.91
135.22
129.64
129.15
128.30
127.17
126.88
125.88
125.01
114.58
77.55
77.23
76.92
55.31
39.31
23.13
S
N
O
4b
ppm 200 175 150 125 100 75 50 25 0
Current Data Parameters
NAME 20100802
EXPNO 5
PROCNO 1
F2 - Acquisition Parameters
Date_ 20100802
INSTRUM spect
PROBHD 5 mm BBO BB-1H
PULPROG zgpg30
TD 65536
SOLVENT CDCl3
NS 200
DS 0
SWH 25062.656 Hz
FIDRES 0.382426 Hz
AQ 1.3074932 sec
RG 8192
DW 19.950 usec
DE 6.50 usec
TE 297.5 K
D1 2.00000000 sec
d11 0.03000000 sec
DELTA 1.89999998 sec
MCREST 0.00000000 sec
MCWRK 0.01500000 sec
CHANNEL f1
NUC1 13C
P1 10.00 usec
PL1 5.00 dB
SFO1 100.6242595 MHz
CHANNEL f2
CPDPRG2 waltz16
NUC2 1H
PCPD2 90.00 usec
PL2 4.00 dB
PL12 20.80 dB
PL13 23.80 dB
SFO2 400.1319000 MHz
F2 - Processing parameters
SI 32768
SF 100.6127681 MHz
WDW EM
SSB 0
LB 1.00 Hz
GB 0
PC 1.40
1D NMR plot parameters
CX 20.00 cm
CY 8.90 cm
F1P 220.000 ppm
F1 22134.81 Hz
F2P -10.000 ppm
F2 -1006.13 Hz

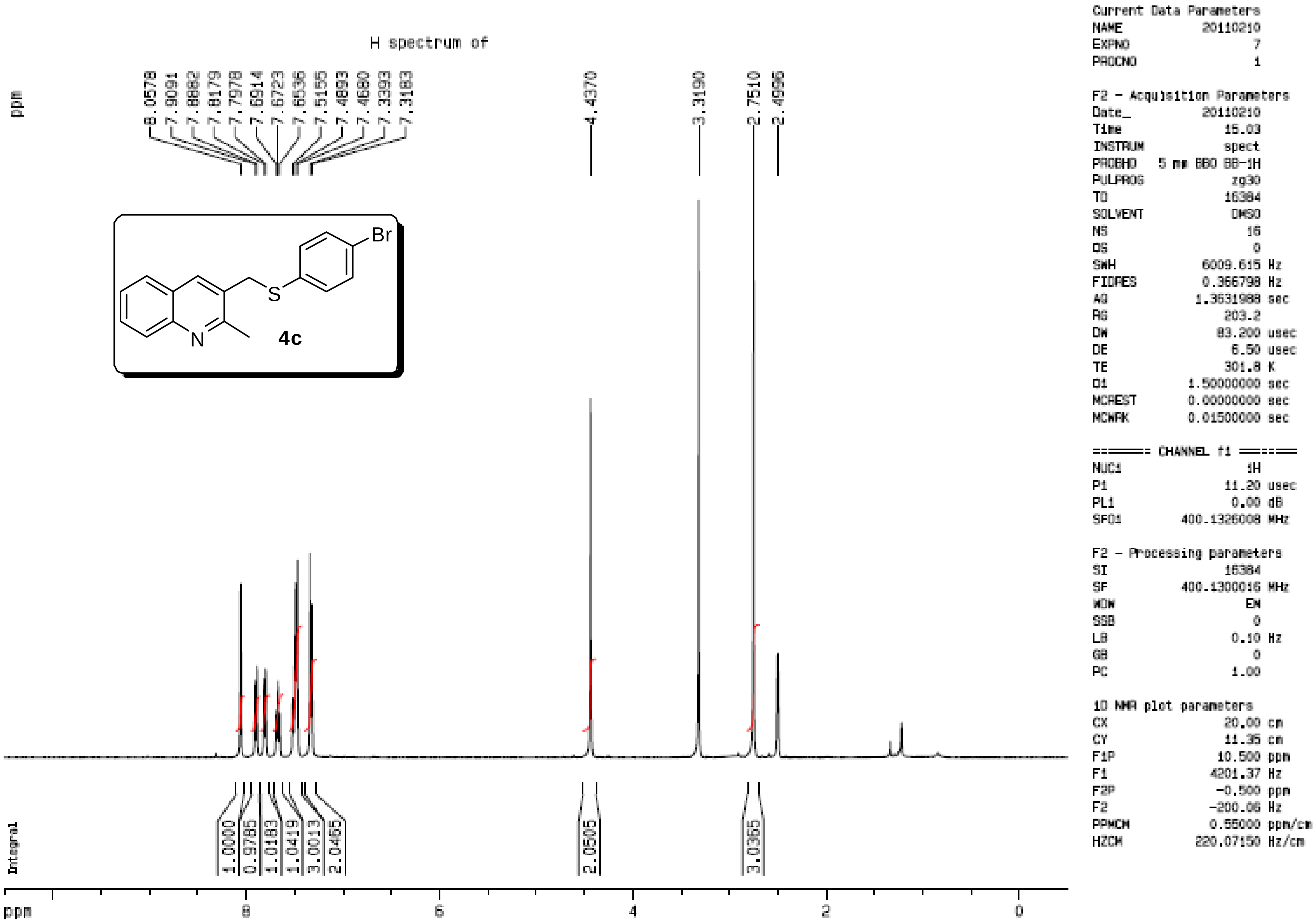
H spectrum of
ppm
8.0578
7.9091
7.8882
7.8179
7.7978
7.6914
7.6723
7.6536
7.5155
7.4893
7.4680
7.3393
7.3183
4.4370
3.3190
2.7510
2.4996
Br
S
N
4c
Integral
1.0000
0.9785
1.0183
1.0419
3.0013
2.0465
2.0505
3.0365
ppm
8
6
4
2
0
Current Data Parameters
NAME 20110210
EXPNO 7
PROCNO 1
F2 - Acquisition Parameters
Date_ 20110210
Time 15.03
INSTRUM spect
PROBHD 5 mm BBO BB-1H
PULPROG zg30
TD 16384
SOLVENT DMSO
NS 16
DS 0
SWH 6009.615 Hz
FIDRES 0.366798 Hz
AQ 1.3631988 sec
RG 203.2
DW 83.200 usec
DE 6.50 usec
TE 301.8 K
D1 1.50000000 sec
MCREST 0.00000000 sec
MCWRK 0.01500000 sec
======== CHANNEL f1 ========
NUC1 1H
P1 11.20 usec
PL1 0.00 dB
SFO1 400.1326008 MHz
F2 - Processing parameters
SI 16384
SF 400.1300016 MHz
WDW EM
SSB 0
LB 0.10 Hz
GB 0
PC 1.00
1D NMR plot parameters
CX 20.00 cm
CY 11.35 cm
F1P 10.500 ppm
F1 4201.37 Hz
F2P -0.500 ppm
F2 -200.06 Hz
PPMCM 0.55000 ppm/cm
HZCM 220.07150 Hz/cm

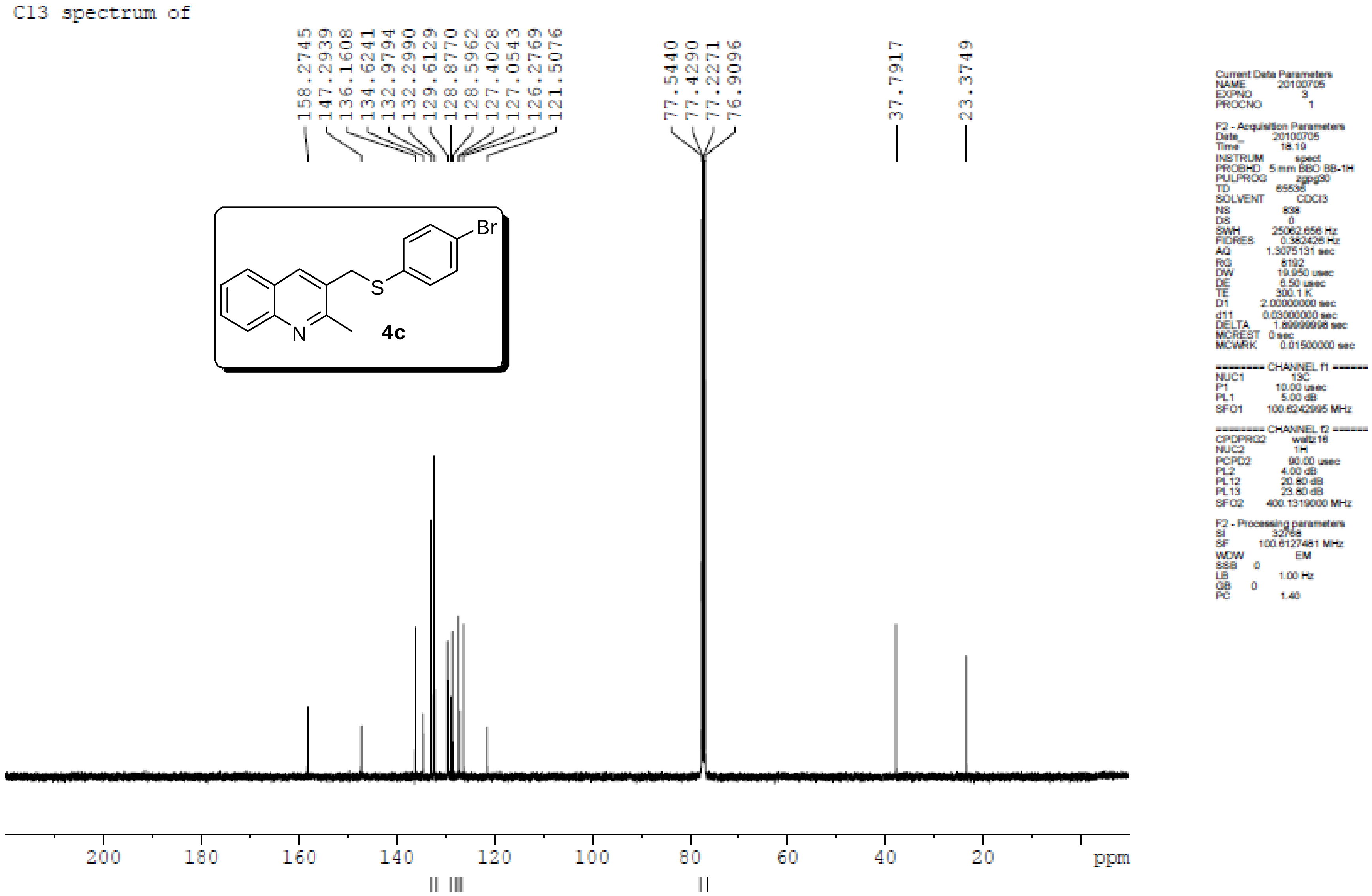
C13 spectrum of
158.2745
147.2939
136.1608
134.6241
132.9794
132.2990
129.6129
128.8770
128.5962
127.4028
127.0543
126.2769
121.5076
77.5440
77.4290
77.2271
76.9096
37.7917
23.3749
Br
S
N
4c
200
180
160
140
120
100
80
60
40
20
ppm
Current Data Parameters
NAME 20100705
EXPNO 3
PROCNO 1
F2 - Acquisition Parameters
Date_ 20100705
Time 18.19
INSTRUM spect
PROBHD 5 mm BBO BB-1H
PULPROG zgpg30
TD 65536
SOLVENT CDCl3
NS 838
DS 0
SWH 25062.656 Hz
FIDRES 0.382426 Hz
AQ 1.3075131 sec
RG 8192
DW 19.950 usec
DE 6.50 usec
TE 300.1 K
D1 2.00000000 sec
d11 0.03000000 sec
MCREST 0 sec
MCWRK 0.01500000 sec
======== CHANNEL f1 ======
NUC1 13C
P1 10.00 usec
PL1 5.00 dB
======== CHANNEL f2 ======
CPDPRG2 waltz16
NUC2 1H
PCPD2 90.00 usec
PL2 4.00 dB
PL12 20.80 dB
PL13 23.80 dB
SFO2 400.1319000 MHz
F2 - Processing parameters
SI 32768
SF 100.6127481 MHz
WDW EM
SSB 0
LB 1.00 Hz
GB 0
PC 1.40

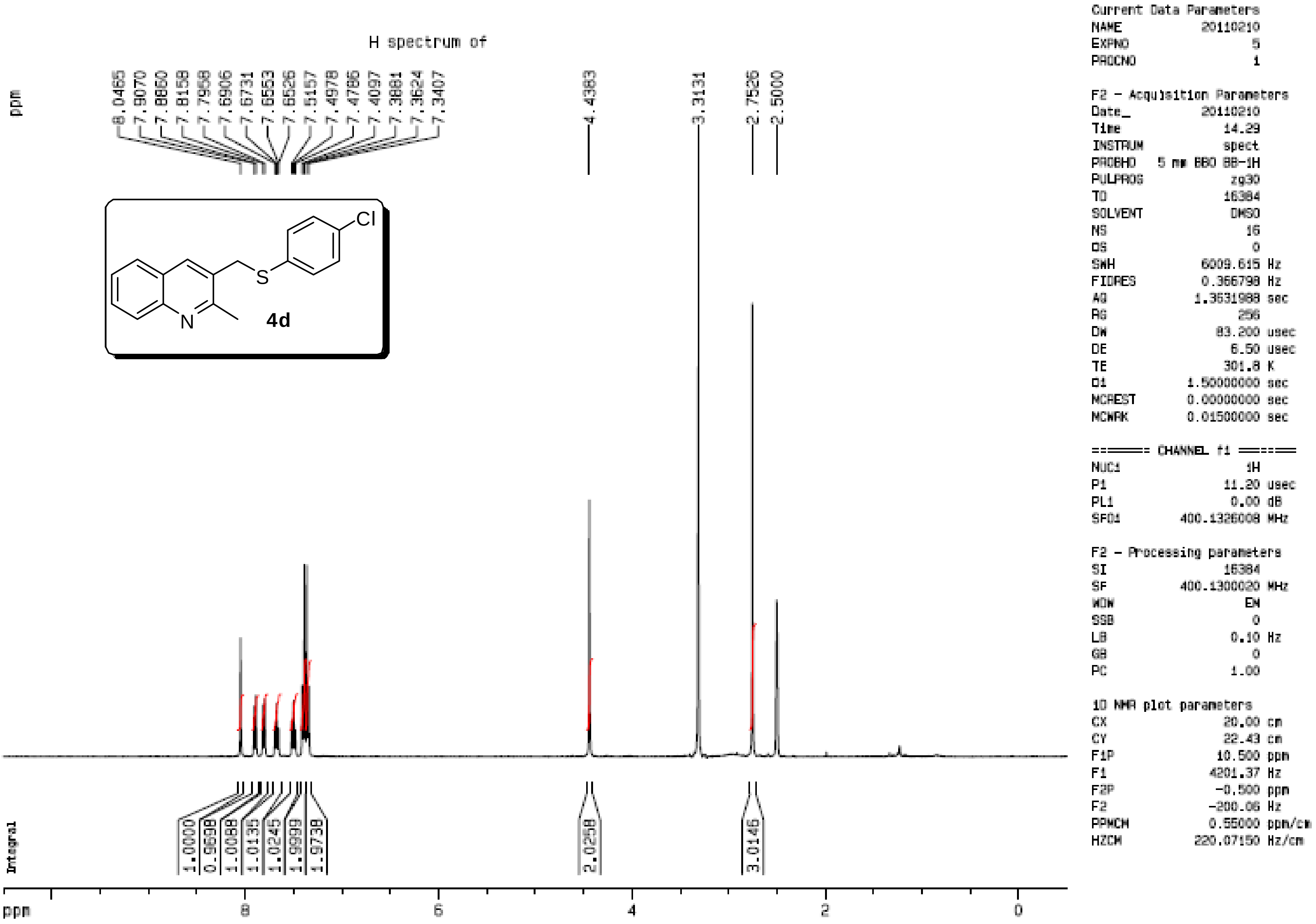
H spectrum of
ppm
8.0465
7.9070
7.8860
7.8158
7.7958
7.6906
7.6731
7.6553
7.6526
7.5157
7.4978
7.4786
7.4097
7.3881
7.3624
7.3407
4.4383
3.3131
2.7526
2.5000
Cl
S
N
4d
Integral
1.0000
0.9698
1.0088
1.0135
1.0245
1.9999
1.9738
2.0258
3.0146
ppm
8
6
4
2
0
Current Data Parameters
NAME 20110210
EXPNO 5
PROCNO 1
F2 - Acquisition Parameters
Date_ 20110210
Time 14.29
INSTRUM spect
PROBHD 5 mm BBO BB-1H
PULPROG zg30
TD 16384
SOLVENT DMSO
NS 16
DS 0
SWH 6009.615 Hz
FIDRES 0.366798 Hz
AQ 1.3631988 sec
RG 256
DW 83.200 usec
DE 6.50 usec
TE 301.8 K
D1 1.50000000 sec
MCREST 0.00000000 sec
MCWRK 0.01500000 sec
======= CHANNEL f1 =======
NUC1 1H
P1 11.20 usec
PL1 0.00 dB
SFO1 400.1326008 MHz
F2 - Processing parameters
SI 16384
SF 400.1300020 MHz
WDW EM
SSB 0
LB 0.10 Hz
GB 0
PC 1.00
1D NMR plot parameters
CX 20.00 cm
CY 22.43 cm
F1P 10.500 ppm
F1 4201.37 Hz
F2P -0.500 ppm
F2 -200.06 Hz
PPMCM 0.55000 ppm/cm
HZCM 220.07150 Hz/cm

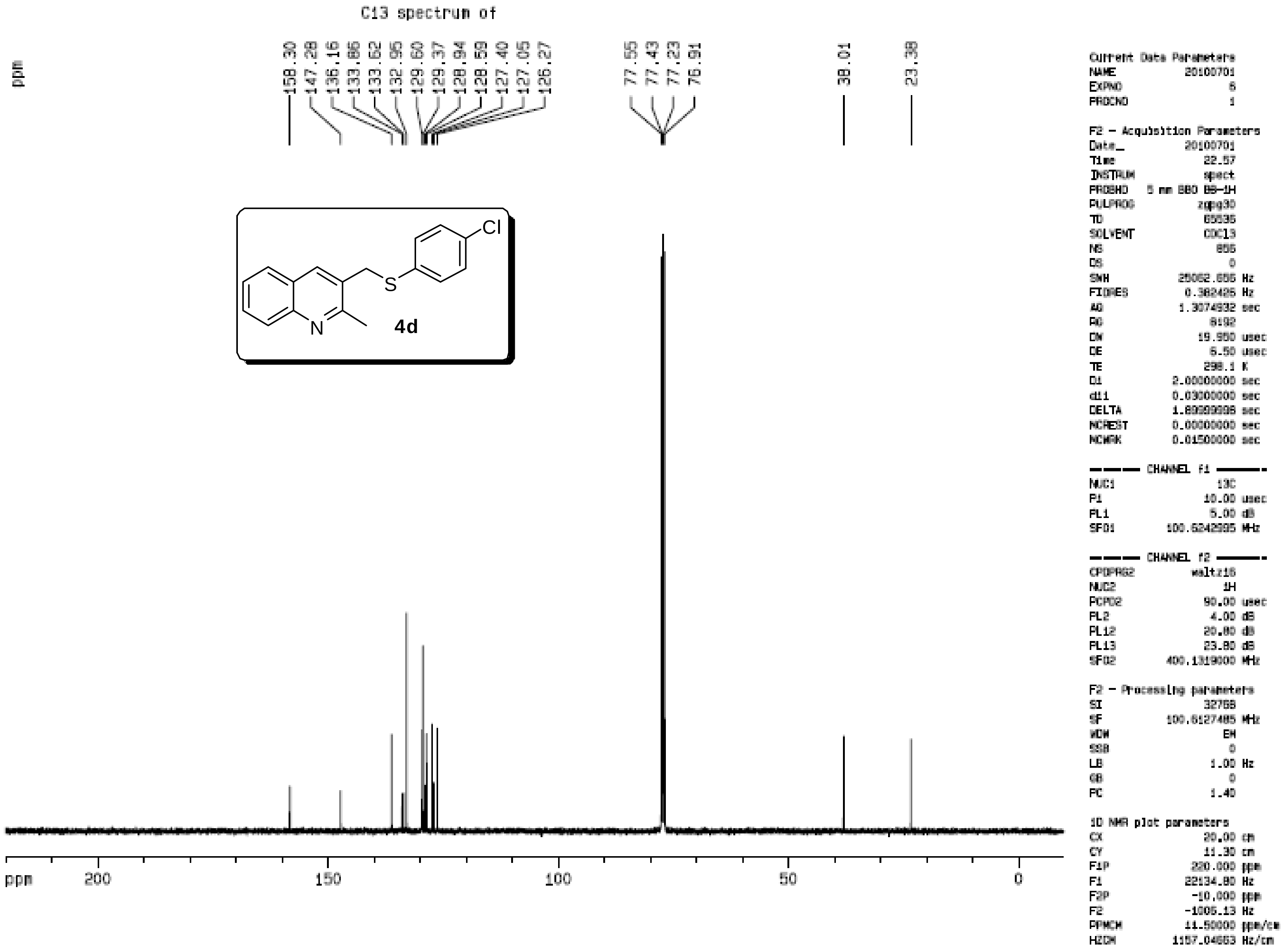
C13 spectrum of
ppm
158.30
147.28
136.16
133.86
133.62
132.95
129.60
129.37
128.94
128.59
127.40
127.05
126.27
77.55
77.43
77.23
76.91
38.01
23.38
Cl
S
N
4d
ppm 200 150 100 50 0
Current Data Parameters
NAME 20100701
EXPNO 6
PROCNO 1
F2 - Acquisition Parameters
Date_ 20100701
Time 22.57
INSTRUM spect
PROBHD 5 mm BBO BB-1H
PULPROG zgpg30
TD 65536
SOLVENT CDCl3
NS 856
DS 0
SWH 25062.656 Hz
FIDRES 0.382426 Hz
AQ 1.3074932 sec
RG 8192
DW 19.950 usec
DE 6.50 usec
TE 298.1 K
D1 2.00000000 sec
d11 0.03000000 sec
DELTA 1.89999998 sec
MCREST 0.00000000 sec
MCWRK 0.01500000 sec
CHANNEL f1
NUC1 13C
P1 10.00 usec
PL1 5.00 dB
SFO1 100.6242985 MHz
CHANNEL f2
CPDPRG2 waltz16
NUC2 1H
PCPD2 90.00 usec
PL2 4.00 dB
PL12 20.80 dB
PL13 23.80 dB
SFO2 400.1319000 MHz
F2 - Processing parameters
SI 32768
SF 100.6127485 MHz
WDW EM
SSB 0
LB 1.00 Hz
GB 0
PC 1.40
1D NMR plot parameters
CX 20.00 cm
CY 11.30 cm
F1P 220.000 ppm
F1 22134.80 Hz
F2P -10.000 ppm
F2 -1006.13 Hz
PPMCM 11.50000 ppm/cm
HZCM 1157.04663 Hz/cm

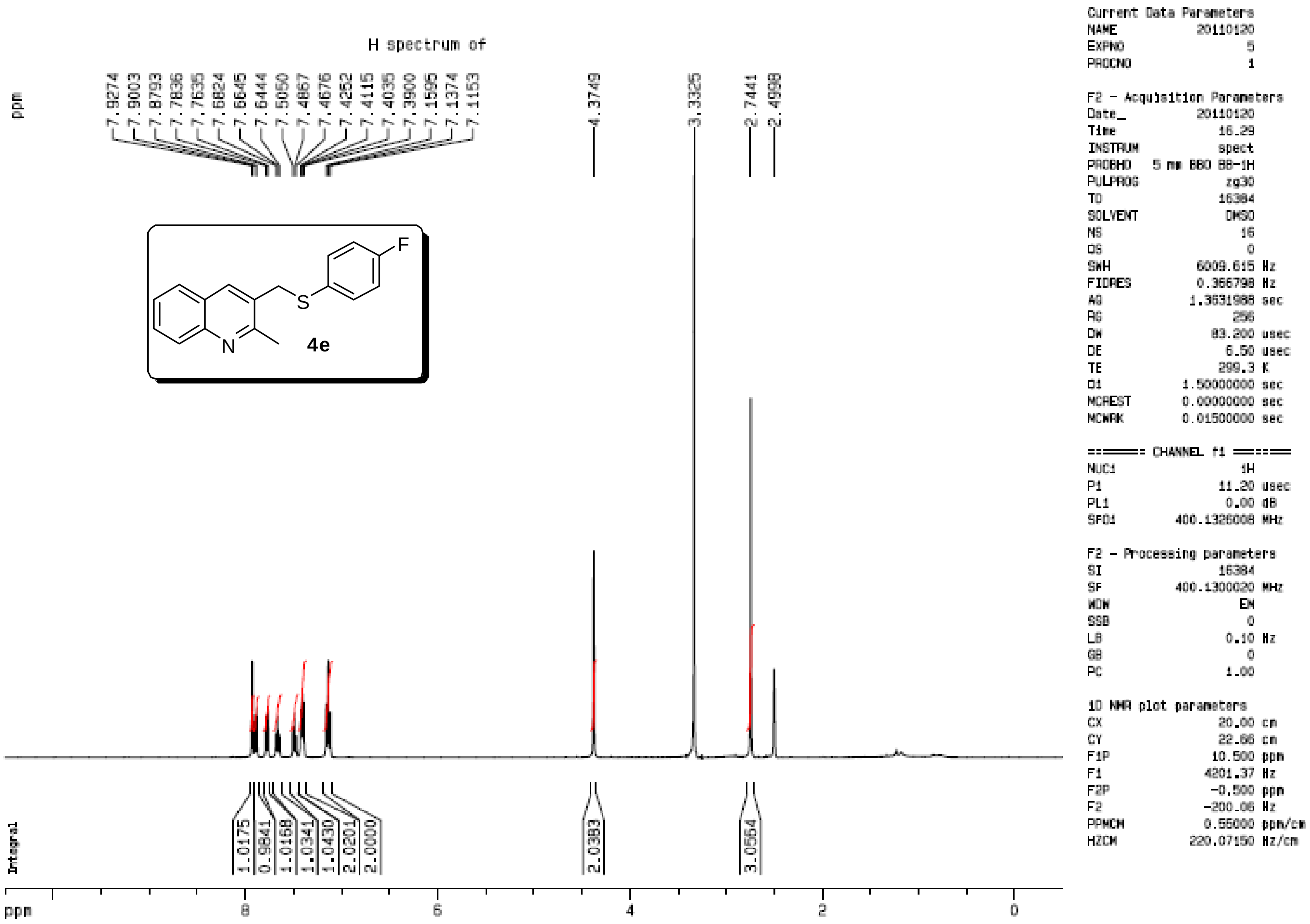
H spectrum of
ppm
7.9274
7.9003
7.8793
7.7836
7.7635
7.6824
7.6645
7.6444
7.5050
7.4867
7.4676
7.4252
7.4115
7.4035
7.3900
7.1595
7.1374
7.1153
4.3749
3.3325
2.7441
2.4998
F
S
N
4e
Integral
1.0175
0.9841
1.0168
1.0341
1.0430
2.0201
2.0000
2.0383
3.0564
ppm
8
6
4
2
0
Current Data Parameters
NAME 20110120
EXPNO 5
PROCNO 1
F2 - Acquisition Parameters
Date_ 20110120
Time 16.29
INSTRUM spect
PROBHD 5 mm BBO BB-1H
PULPROG zg30
TD 16384
SOLVENT DMSO
NS 16
DS 0
SWH 6009.615 Hz
FIDRES 0.366798 Hz
AQ 1.3631988 sec
RG 256
DW 83.200 usec
DE 6.50 usec
TE 299.3 K
D1 1.50000000 sec
MCREST 0.00000000 sec
MCWRK 0.01500000 sec
======== CHANNEL f1 ========
NUC1 1H
P1 11.20 usec
PL1 0.00 dB
SFO1 400.1326008 MHz
F2 - Processing parameters
SI 16384
SF 400.1300020 MHz
WDW EM
SSB 0
LB 0.10 Hz
GB 0
PC 1.00
1D NMR plot parameters
CX 20.00 cm
CY 22.66 cm
F1P 10.500 ppm
F1 4201.37 Hz
F2P -0.500 ppm
F2 -200.06 Hz
PPMCM 0.55000 ppm/cm
HZCM 220.07150 Hz/cm

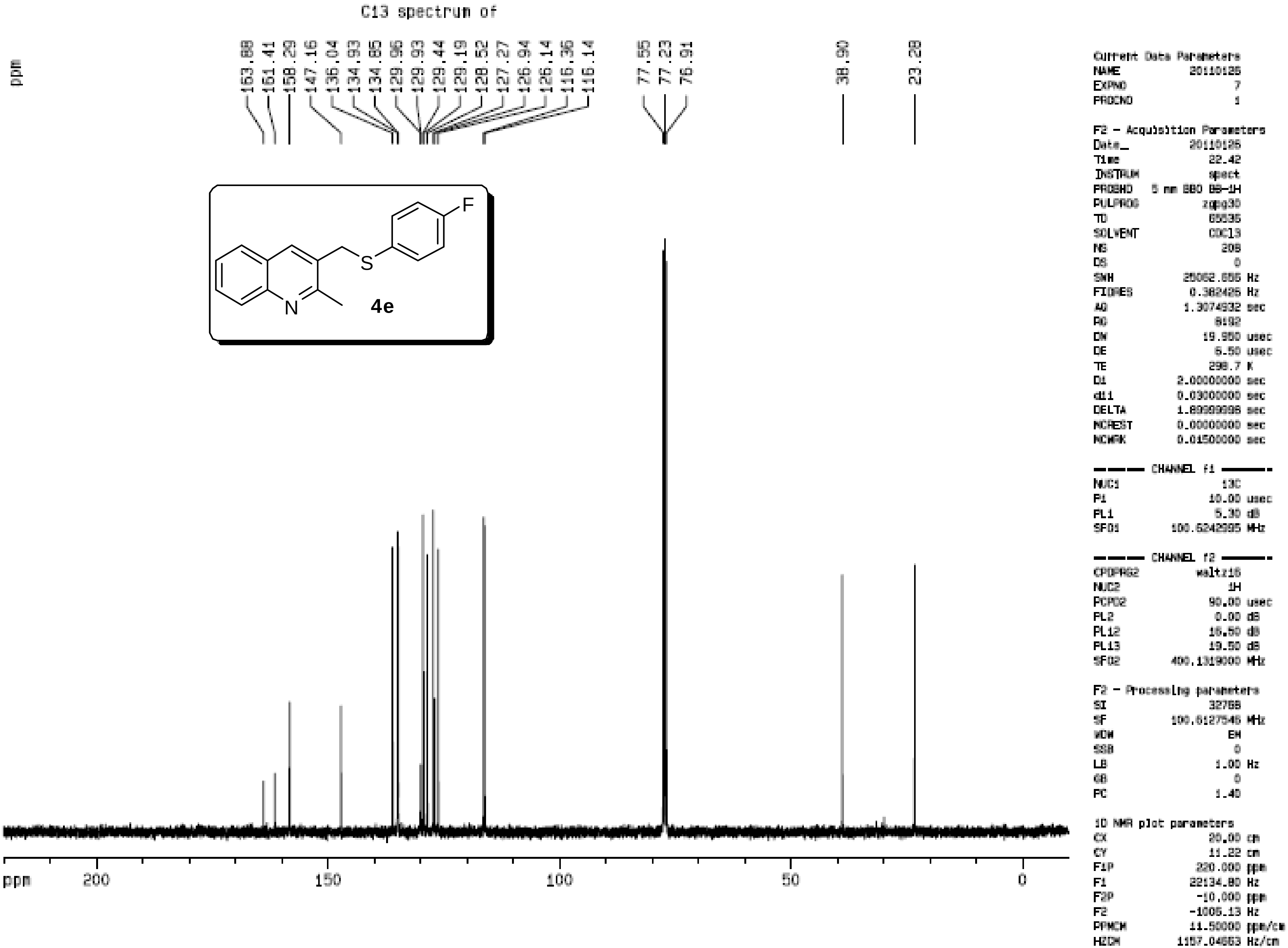
C13 spectrum of
153.88
151.41
158.29
147.16
136.04
134.93
134.85
129.96
129.93
129.44
129.19
128.52
127.27
126.94
126.14
116.36
116.14
77.55
77.23
76.91
38.90
23.28
F
S
N
4e
ppm
ppm 200 150 100 50 0
Current Data Parameters
NAME 20110126
EXPNO 7
PROCNO 1
F2 - Acquisition Parameters
Date_ 20110126
Time 22.42
INSTRUM spect
PROBHD 5 mm BBO BB-1H
PULPROG zgpg30
TD 65536
SOLVENT CDCl3
NS 208
DS 0
SWH 25062.656 Hz
FIDRES 0.382426 Hz
AQ 1.3074932 sec
RG 8192
DW 19.950 usec
DE 6.50 usec
TE 298.7 K
D1 2.00000000 sec
d11 0.03000000 sec
DELTA 1.89999998 sec
MCREST 0.00000000 sec
MCWRK 0.01500000 sec
CHANNEL f1
NUC1 13C
P1 10.00 usec
PL1 5.30 dB
SFO1 100.6242995 MHz
CHANNEL f2
CPDPRG2 waltz16
NUC2 1H
PCPD2 90.00 usec
PL2 0.00 dB
PL12 16.50 dB
PL13 19.50 dB
SFO2 400.1319000 MHz
F2 - Processing parameters
SI 32768
SF 100.6127546 MHz
WDW EM
SSB 0
LB 1.00 Hz
GB 0
PC 1.40
1D NMR plot parameters
CX 20.00 cm
CY 11.22 cm
F1P 220.000 ppm
F1 22134.80 Hz
F2P -10.000 ppm
F2 -1006.13 Hz
PPMCM 11.50000 ppm/cm
HZCM 1157.04663 Hz/cm

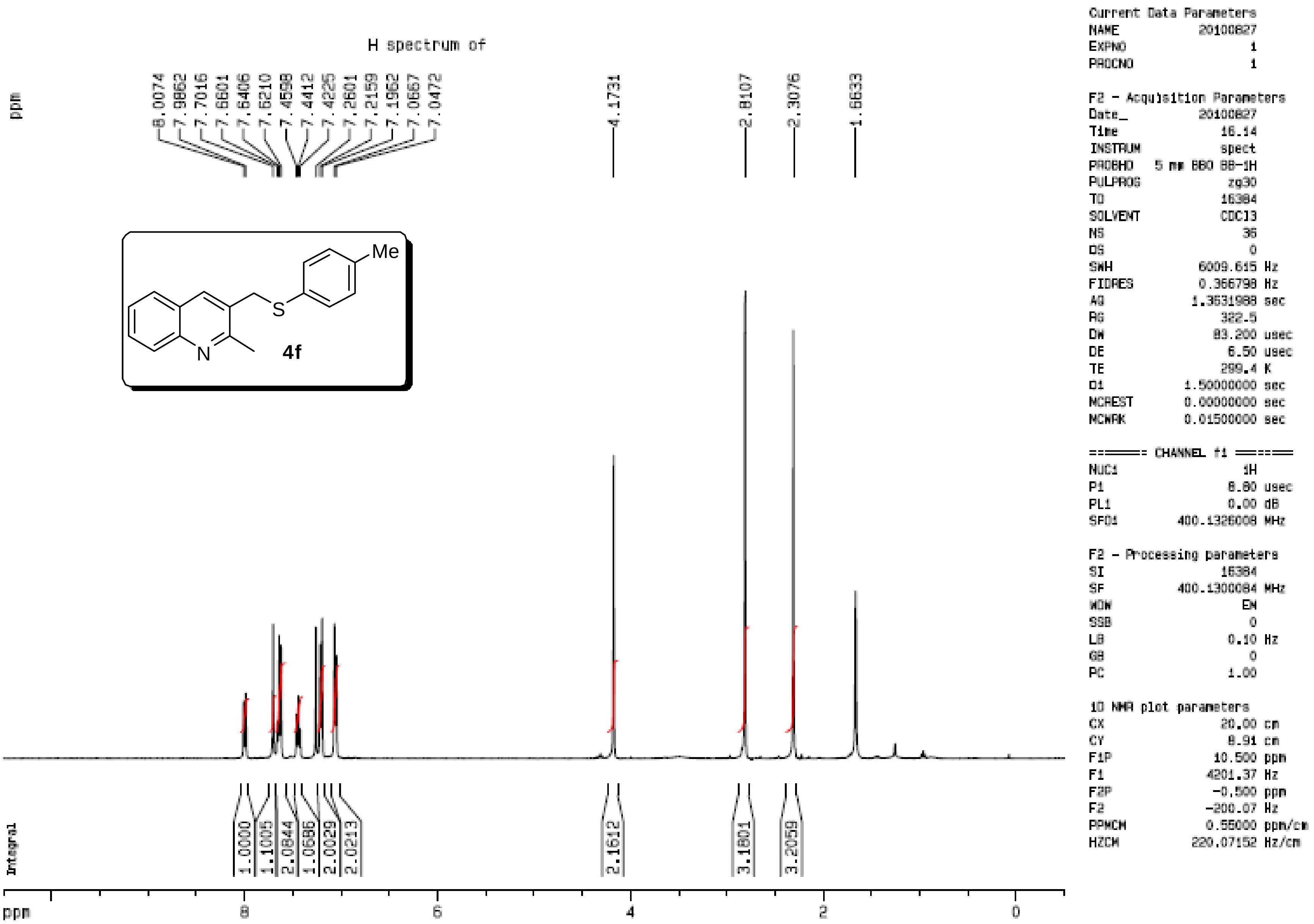
H spectrum of
ppm
8.0074
7.9862
7.7016
7.6801
7.6406
7.6210
7.4598
7.4412
7.4225
7.2601
7.2159
7.1962
7.0667
7.0472
4.1731
2.8107
2.3076
1.6633
Me
S
N
4f
Integral
1.0000
1.1005
2.0844
1.0686
2.0029
2.0213
2.1612
3.1801
3.2059
ppm
8
6
4
2
0
Current Data Parameters
NAME 20100827
EXPNO 1
PROCNO 1
F2 - Acquisition Parameters
Date_ 20100827
Time 16.14
INSTRUM spect
PROBHD 5 mm BBO BB-1H
PULPROG zg30
TD 16384
SOLVENT CDCl3
NS 36
DS 0
SWH 6009.615 Hz
FIDRES 0.366798 Hz
AQ 1.3631988 sec
RG 322.5
DW 83.200 usec
DE 6.50 usec
TE 299.4 K
D1 1.50000000 sec
MCREST 0.00000000 sec
MCWRK 0.01500000 sec
======== CHANNEL f1 ========
NUC1 1H
P1 8.80 usec
PL1 0.00 dB
SFO1 400.1326008 MHz
F2 - Processing parameters
SI 16384
SF 400.1300084 MHz
WDW EM
SSB 0
LB 0.10 Hz
GB 0
PC 1.00
1D NMR plot parameters
CX 20.00 cm
CY 8.91 cm
F1P 10.500 ppm
F1 4201.37 Hz
F2P -0.500 ppm
F2 -200.07 Hz
PPMCM 0.55000 ppm/cm
HZCM 220.07152 Hz/cm

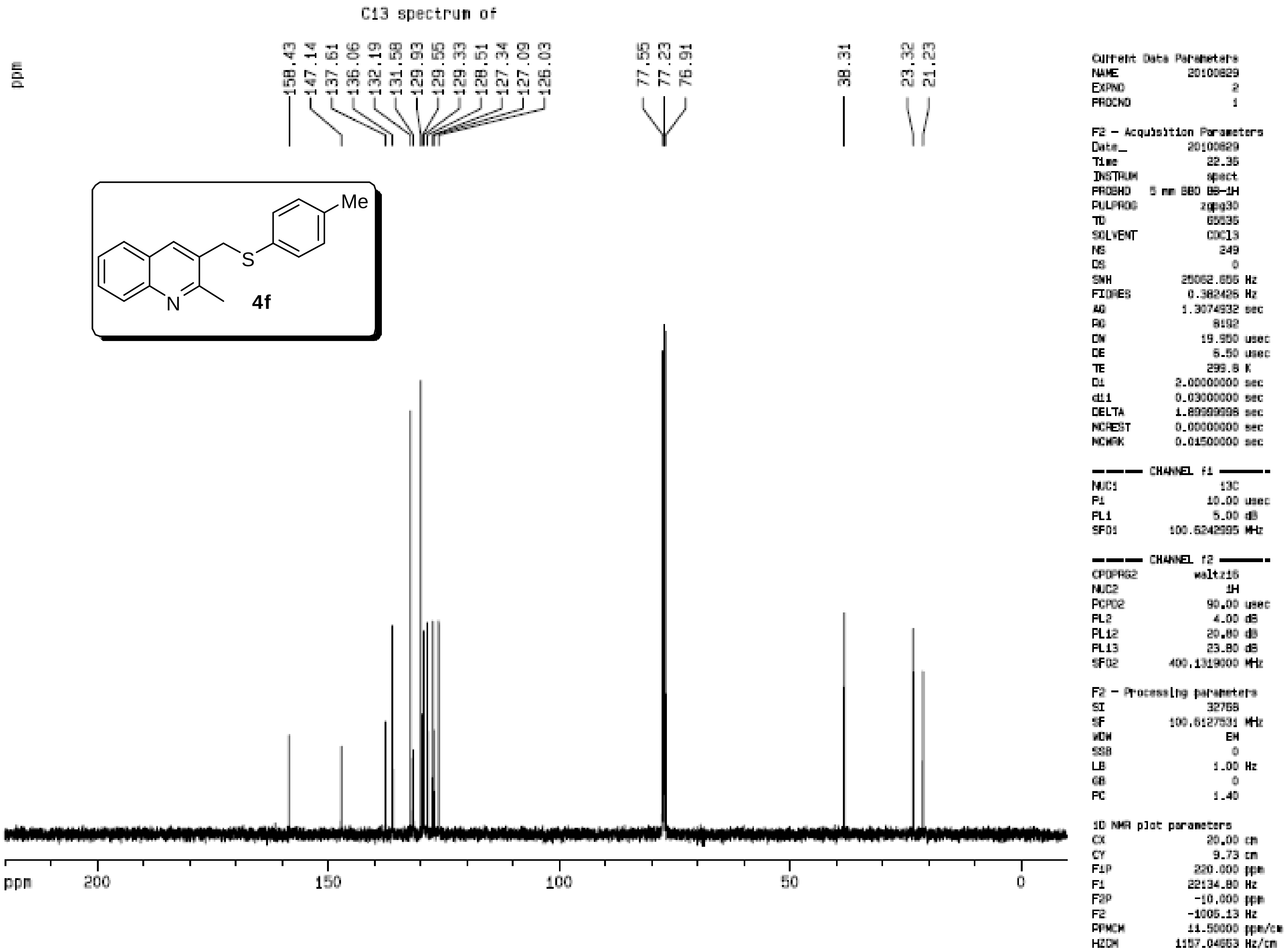
C13 spectrum of
ppm
158.43
147.14
137.61
136.06
132.19
131.58
129.93
129.55
129.33
128.51
127.34
127.09
126.03
77.55
77.23
76.91
38.31
23.32
21.23
Me
S
N
4f
ppm 200 150 100 50 0
Current Data Parameters
NAME 20100829
EXPNO 2
PROCNO 1
F2 - Acquisition Parameters
Date_ 20100829
Time 22.35
INSTRUM spect
PROBHD 5 mm BBO BB-1H
PULPROG zgpg30
TD 65536
SOLVENT CDCl3
NS 249
DS 0
SWH 25062.656 Hz
FIDRES 0.382426 Hz
AQ 1.3074932 sec
RG 8192
DW 19.950 usec
DE 6.50 usec
TE 299.8 K
D1 2.00000000 sec
d11 0.03000000 sec
DELTA 1.89999998 sec
MCREST 0.00000000 sec
MCWRK 0.01500000 sec
CHANNEL f1
NUC1 13C
P1 10.00 usec
PL1 5.00 dB
SFO1 100.6242995 MHz
CHANNEL f2
CPDPRG2 waltz16
NUC2 1H
PCPD2 90.00 usec
PL2 4.00 dB
PL12 20.80 dB
PL13 23.80 dB
SFO2 400.1319000 MHz
F2 - Processing parameters
SI 32768
SF 100.6127531 MHz
WDW EM
SSB 0
LB 1.00 Hz
GB 0
PC 1.40
1D NMR plot parameters
CX 20.00 cm
CY 9.73 cm
F1P 220.000 ppm
F1 22134.80 Hz
F2P -10.000 ppm
F2 -1006.13 Hz
PPMCM 11.50000 ppm/cm
HZCM 1157.04663 Hz/cm

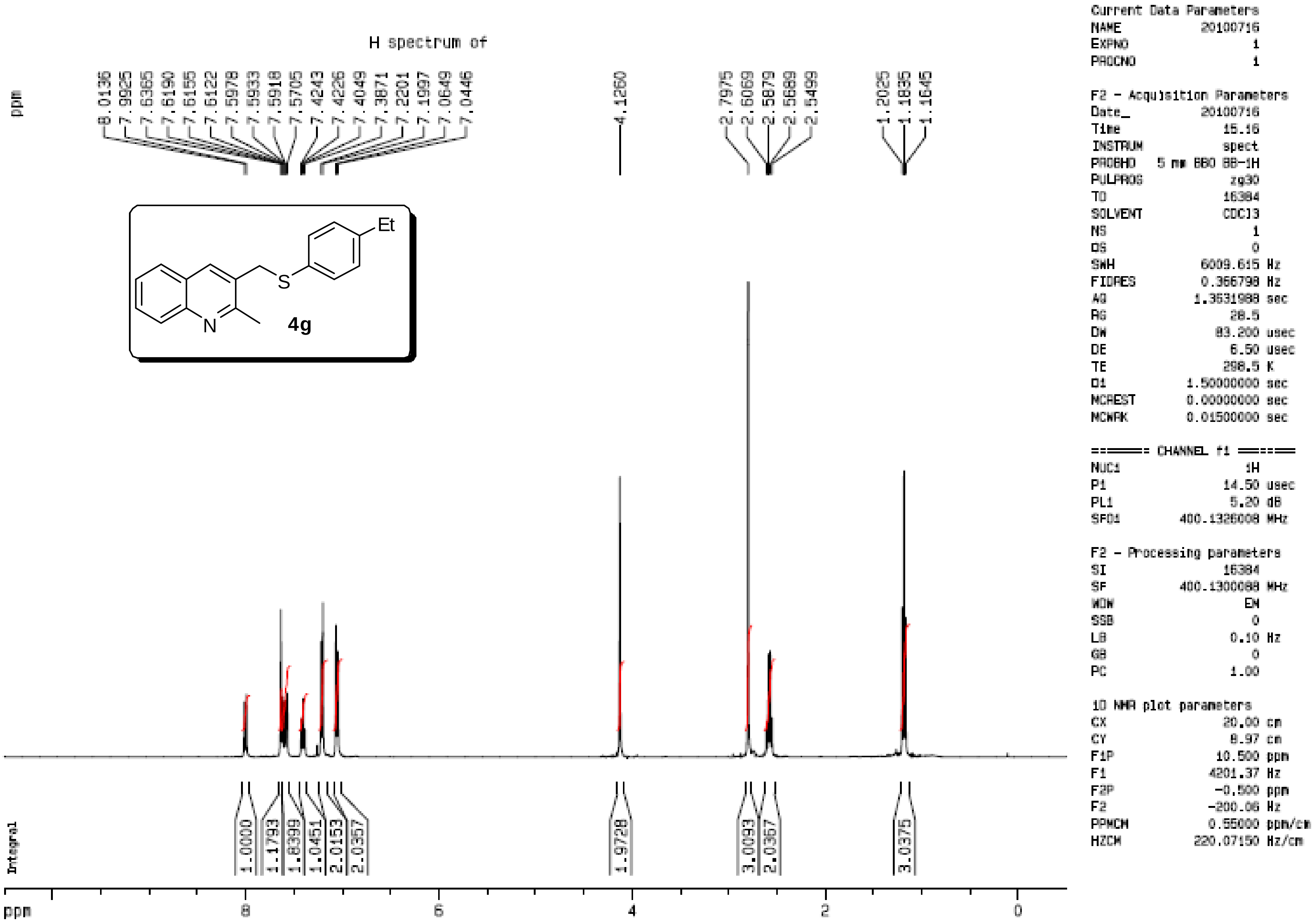
H spectrum of
8.0136
7.9925
7.6365
7.6190
7.6155
7.6122
7.5978
7.5933
7.5918
7.5705
7.4243
7.4226
7.4049
7.3871
7.2201
7.1997
7.0649
7.0446
4.1260
2.7975
2.6069
2.5879
2.5689
2.5499
1.2025
1.1835
1.1645
Et
S
N
4g
Integral
1.0000
1.1793
1.8399
1.0451
2.0153
2.0357
1.9728
3.0093
2.0357
3.0375
ppm
8
6
4
2
0
Current Data Parameters
NAME 20100716
EXPNO 1
PROCNO 1
F2 - Acquisition Parameters
Date_ 20100716
Time 15.16
INSTRUM spect
PROBHD 5 mm BBO BB-1H
PULPROG zg30
TD 16384
SOLVENT CDCl3
NS 1
DS 0
SWH 6009.615 Hz
FIDRES 0.366798 Hz
AQ 1.3631988 sec
RG 28.5
DW 83.200 usec
DE 6.50 usec
TE 298.5 K
D1 1.50000000 sec
MCREST 0.00000000 sec
MCWRK 0.01500000 sec
======== CHANNEL f1 ========
NUC1 1H
P1 14.50 usec
PL1 5.20 dB
SFO1 400.1326008 MHz
F2 - Processing parameters
SI 16384
SF 400.1300088 MHz
WDW EM
SSB 0
LB 0.10 Hz
GB 0
PC 1.00
1D NMR plot parameters
CX 20.00 cm
CY 8.97 cm
F1P 10.500 ppm
F1 4201.37 Hz
F2P -0.500 ppm
F2 -200.06 Hz
PPMCM 0.55000 ppm/cm
HZCM 220.07150 Hz/cm

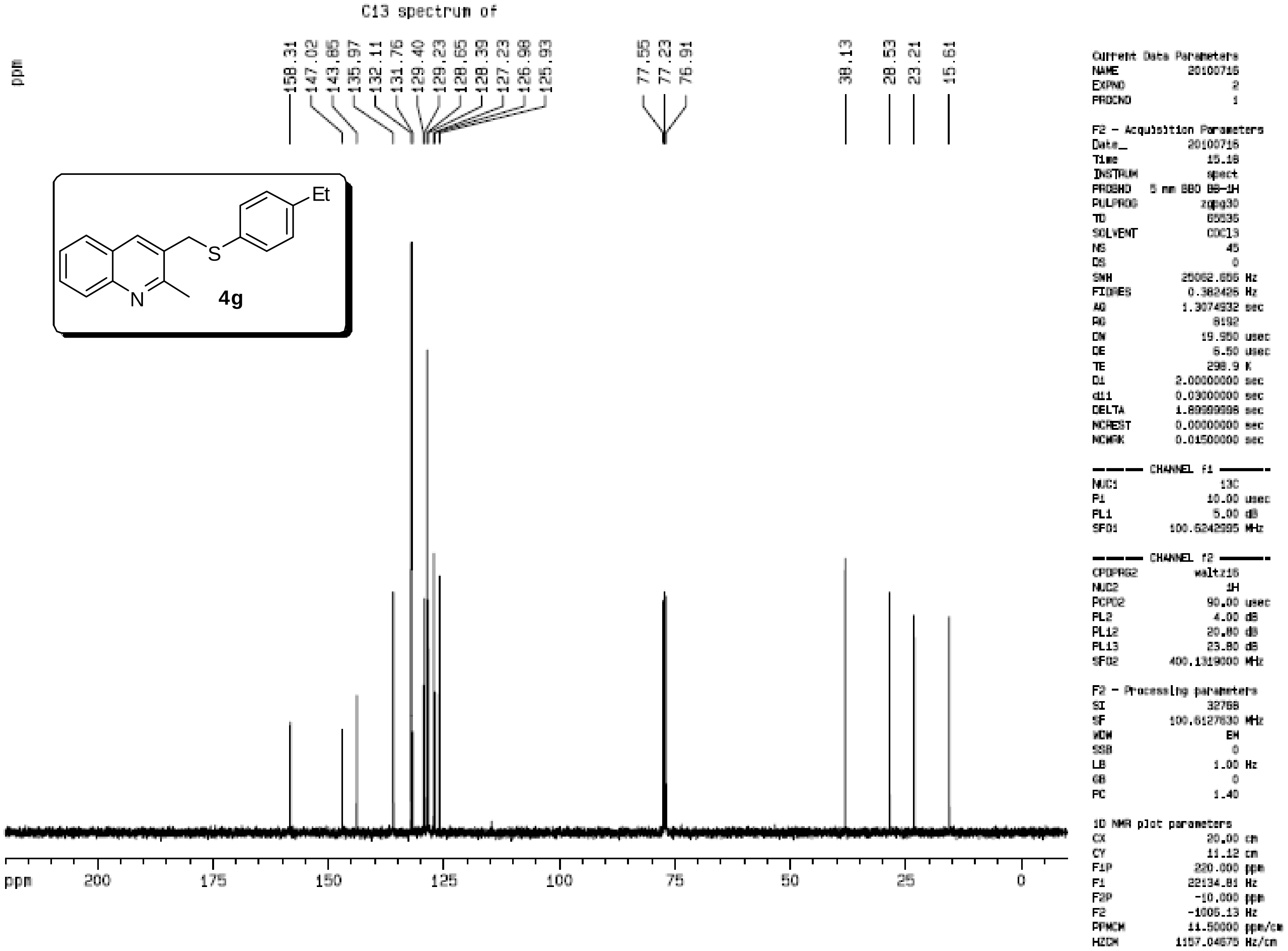
C13 spectrum of
ppm
158.31
147.02
143.65
135.97
132.11
131.76
129.40
129.23
128.65
128.39
127.23
126.98
125.93
77.55
77.23
76.91
38.13
28.53
23.21
15.61
Et
S
N
4g
ppm
200
175
150
125
100
75
50
25
0
Current Data Parameters
NAME 20100716
EXPNO 2
PROCNO 1
F2 - Acquisition Parameters
Date_ 20100716
Time 15.18
INSTRUM spect
PROBHD 5 mm BBO BB-1H
PULPROG zgpg30
TD 65536
SOLVENT CDCl3
NS 45
DS 0
SWH 25062.656 Hz
FIDRES 0.382426 Hz
AQ 1.3074932 sec
RG 8192
DW 19.950 usec
DE 6.50 usec
TE 298.9 K
D1 2.00000000 sec
d11 0.03000000 sec
DELTA 1.89999998 sec
MCREST 0.00000000 sec
MCWRK 0.01500000 sec
CHANNEL f1
NUC1 13C
P1 10.00 usec
PL1 5.00 dB
SFO1 100.6242995 MHz
CHANNEL f2
CPDPRG2 waltz16
NUC2 1H
PCPD2 90.00 usec
PL2 4.00 dB
PL12 20.80 dB
PL13 23.80 dB
SFO2 400.1319000 MHz
F2 - Processing parameters
SI 32768
SF 100.6127630 MHz
WDW EM
SSB 0
LB 1.00 Hz
GB 0
PC 1.40
1D NMR plot parameters
CX 20.00 cm
CY 11.12 cm
F1P 220.000 ppm
F1 22134.81 Hz
F2P -10.000 ppm
F2 -1006.13 Hz
PPMCM 11.50000 ppm/cm
HZCM 1157.04675 Hz/cm

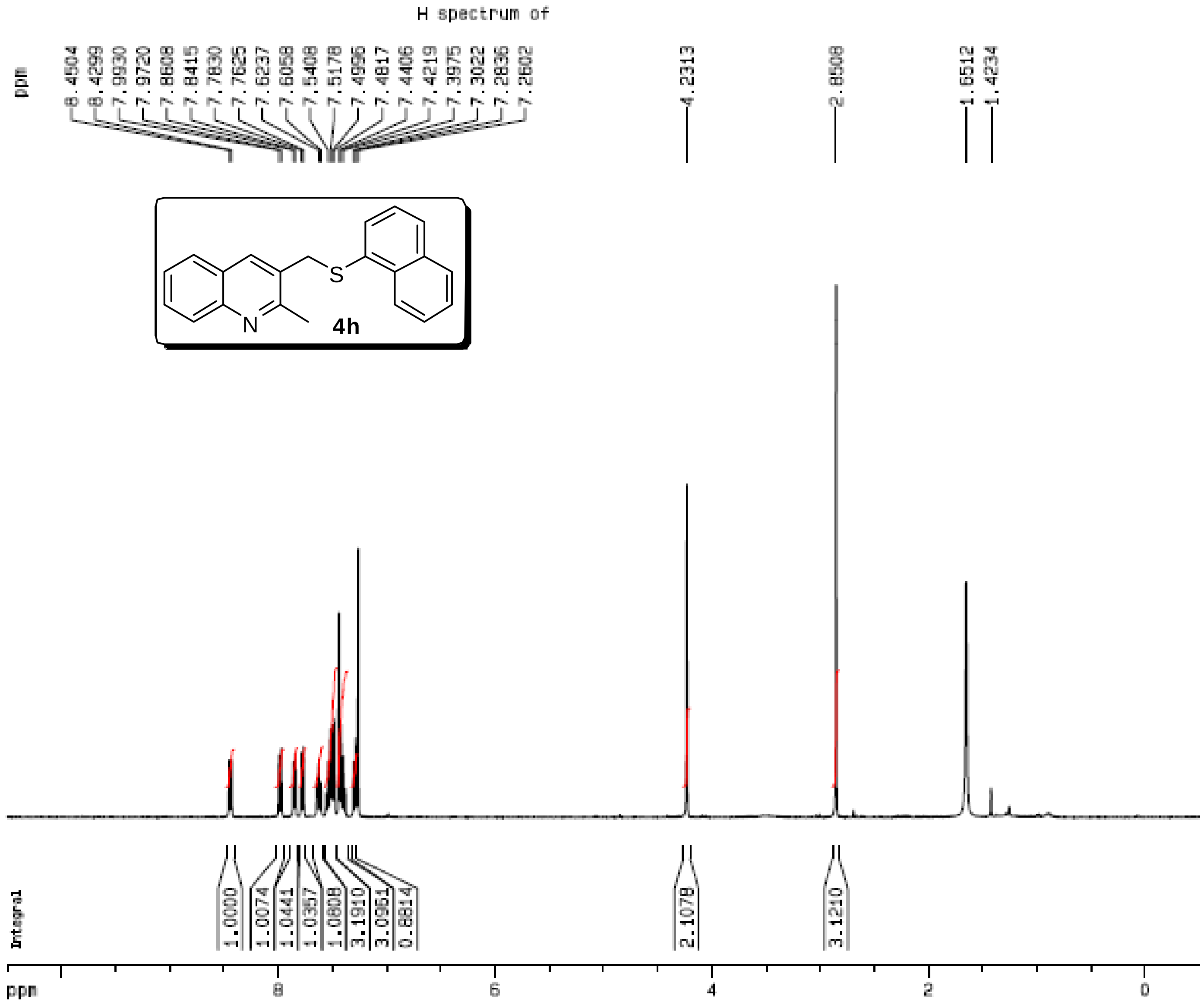

Current Data Parameters
NAME 20100813
EXPNO 1
PROCNO 1

F2 - Acquisition Parameters
Date_ 20100813
Time 10.45
INSTRUM spect
PROBHD 5 mm BBO BB-1H
PULPROG zg30
TD 16384
SOLVENT CDCl3
NS 16
DS 0
SWH 6009.615 Hz
FIDRES 0.366798 Hz
AQ 1.3631988 sec
RG 322.5
DW 83.200 usec
DE 6.50 usec
TE 299.2 K
D1 1.50000000 sec
MCREST 0.00000000 sec
MCWRK 0.01500000 sec

======== CHANNEL f1 ========
NUC1 1H
P1 14.50 usec
PL1 5.20 dB
SFO1 400.1326008 MHz

F2 - Processing parameters
SI 16384
SF 400.1300084 MHz
WDW EM
SSB 0
LB 0.10 Hz
GB 0
PC 1.00

1D NMR plot parameters
CX 20.00 cm
CY 8.94 cm
F1P 10.500 ppm
F1 4201.37 Hz
F2P -0.500 ppm
F2 -200.06 Hz
PPMCM 0.55000 ppm/cm
HZCM 220.07150 Hz/cm

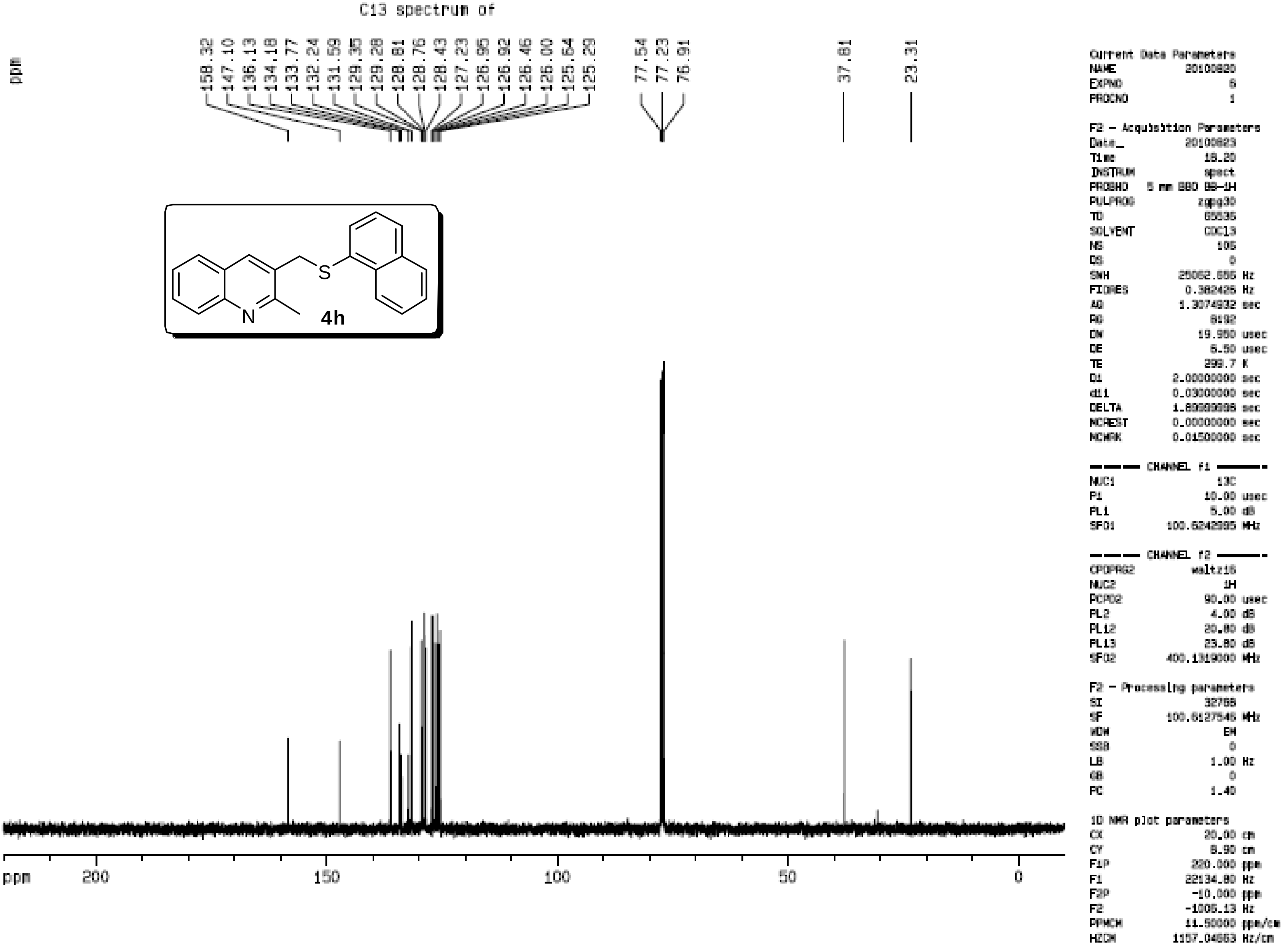
C13 spectrum of
ppm
158.32
147.10
136.13
134.18
133.77
132.24
131.59
129.35
129.28
128.81
128.76
128.43
127.23
126.95
126.92
126.46
126.00
125.64
125.29
77.54
77.23
76.91
37.81
23.31
S
N
4h
ppm 200 150 100 50 0
Current Data Parameters
NAME 20100820
EXPNO 6
PROCNO 1
F2 - Acquisition Parameters
Date_ 20100823
Time 18.20
INSTRUM spect
PROBHD 5 mm BBO BB-1H
PULPROG zgpg30
TD 65536
SOLVENT CDCl3
NS 105
DS 0
SWH 25062.656 Hz
FIDRES 0.382426 Hz
AQ 1.3074932 sec
RG 8192
DW 19.950 usec
DE 6.50 usec
TE 299.7 K
D1 2.00000000 sec
d11 0.03000000 sec
DELTA 1.89999998 sec
MCREST 0.00000000 sec
MCWRK 0.01500000 sec
CHANNEL f1
NUC1 13C
P1 10.00 usec
PL1 5.00 dB
SFO1 100.6242995 MHz
CHANNEL f2
CPDPRG2 waltz16
NUC2 1H
PCPD2 90.00 usec
PL2 4.00 dB
PL12 20.80 dB
PL13 23.80 dB
SFO2 400.1319000 MHz
F2 - Processing parameters
SI 32768
SF 100.6127546 MHz
WDW EM
SSB 0
LB 1.00 Hz
GB 0
PC 1.40
1D NMR plot parameters
CX 20.00 cm
CY 8.90 cm
F1P 220.000 ppm
F1 22134.80 Hz
F2P -10.000 ppm
F2 -1006.13 Hz
PPMCM 11.50000 ppm/cm
HZCM 1157.04663 Hz/cm

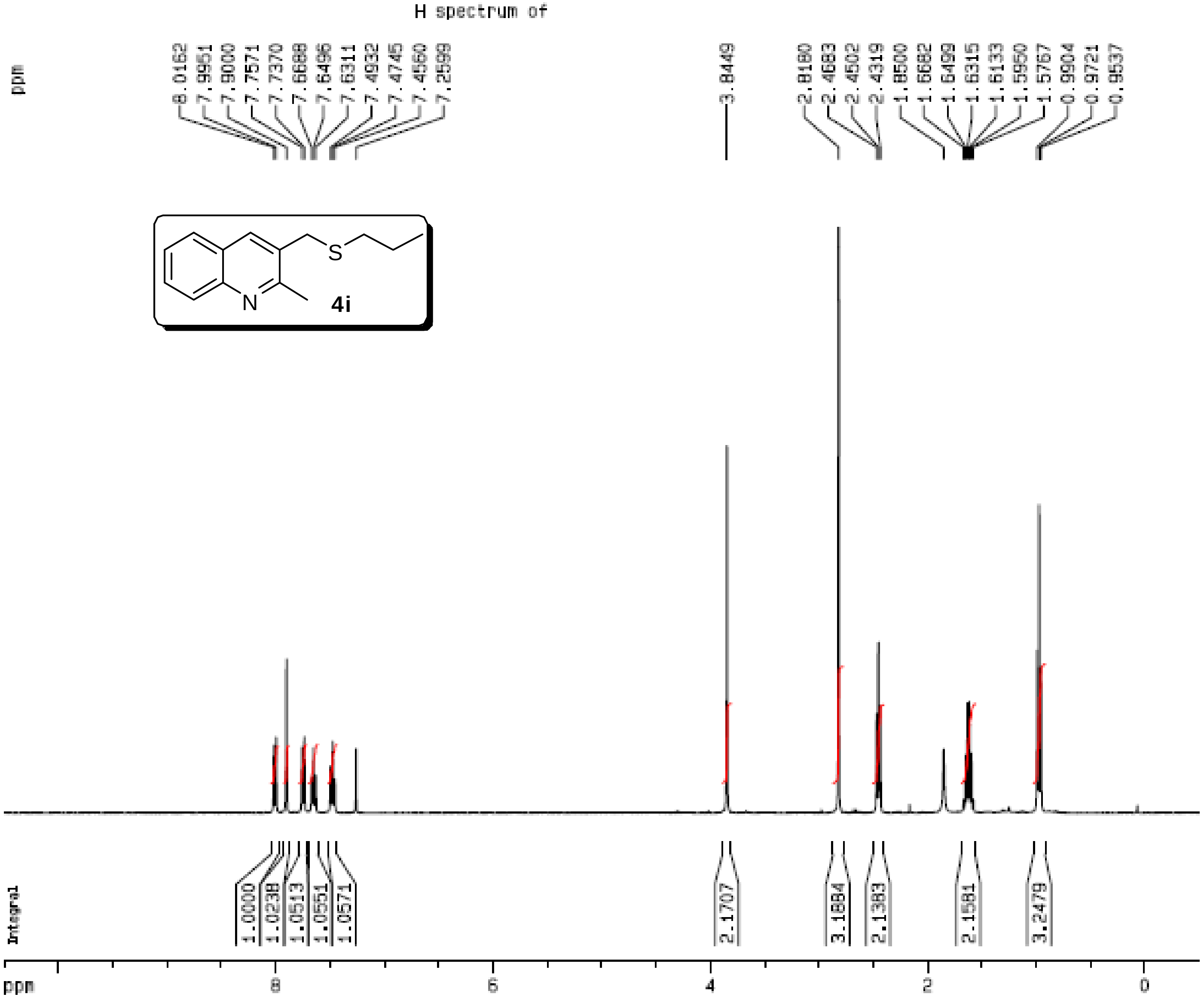

Current Data Parameters
NAME 20100818
EXPNO 5
PROCNO 1

F2 - Acquisition Parameters
Date_ 20100818
Time 16.39
INSTRUM spect
PROBHD 5 mm BBO BB-1H
PULPROG zg30
TD 16384
SOLVENT CDCl3
NS 16
DS 0
SWH 6009.615 Hz
FIDRES 0.366798 Hz
AQ 1.3631988 sec
RG 128
DW 83.200 usec
DE 6.50 usec
TE 297.2 K
D1 1.50000000 sec
MCREST 0.00000000 sec
MCWRK 0.01500000 sec

======== CHANNEL f1 ========
NUC1 1H
P1 8.80 usec
PL1 0.00 dB
SFO1 400.1326008 MHz

F2 - Processing parameters
SI 16384
SF 400.1300088 MHz
WDW EM
SSB 0
LB 0.10 Hz
GB 0
PC 1.00

1D NMR plot parameters
CX 20.00 cm
CY 9.92 cm
F1P 10.500 ppm
F1 4201.37 Hz
F2P -0.500 ppm
F2 -200.06 Hz
PPMCM 0.55000 ppm/cm
HZCM 220.07150 Hz/cm

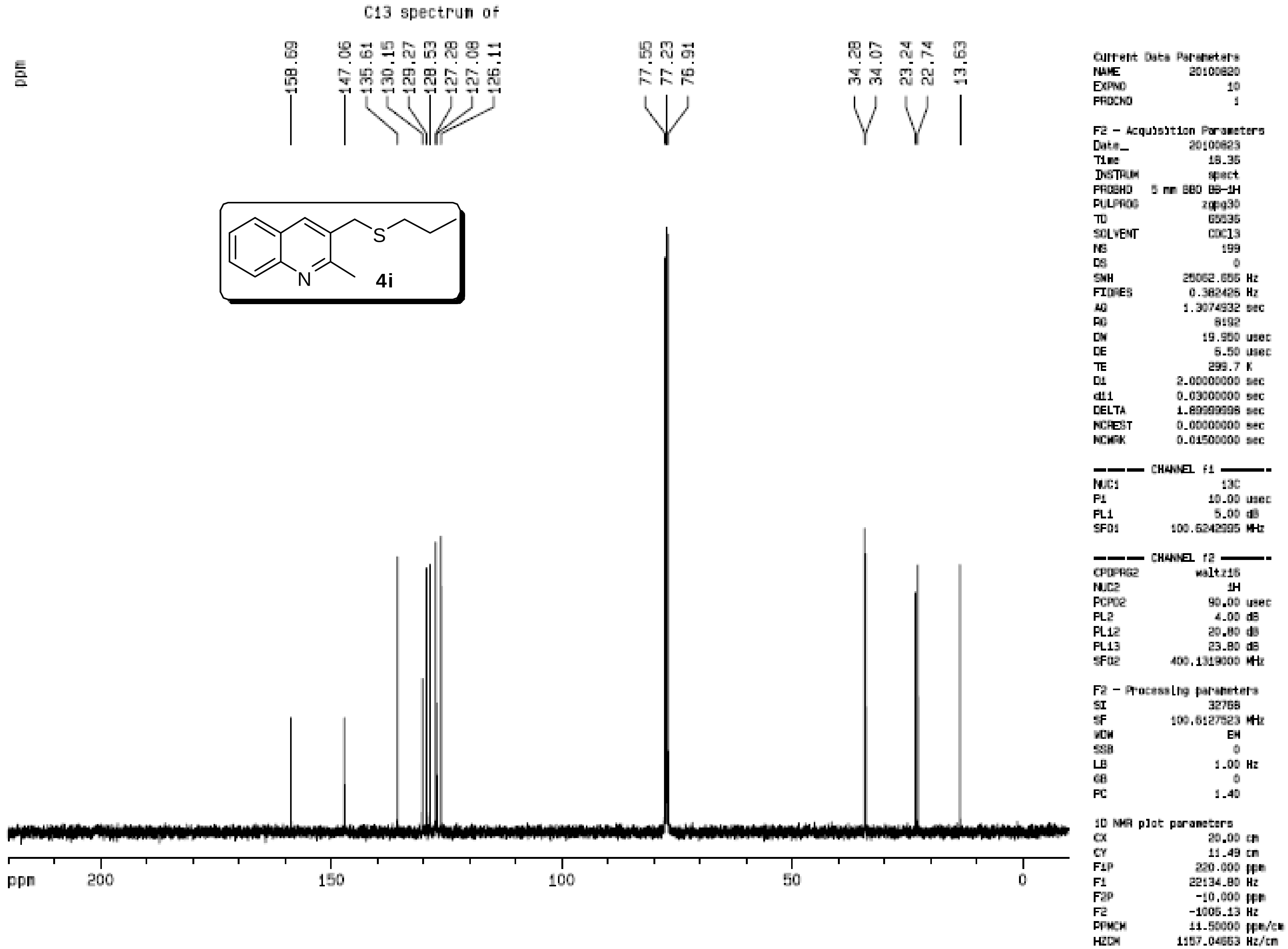
C13 spectrum of
ppm
158.69
147.06
135.61
130.15
129.27
128.53
127.28
127.08
126.11
77.55
77.23
76.91
34.28
34.07
23.24
22.74
13.63
S
N
4i
ppm 200 150 100 50 0
Current Data Parameters
NAME 20100820
EXPNO 10
PROCNO 1
F2 - Acquisition Parameters
Date_ 20100823
Time 18.35
INSTRUM spect
PROBHD 5 mm BBO BB-1H
PULPROG zgpg30
TD 65536
SOLVENT CDCl3
NS 199
DS 0
SWH 25062.656 Hz
FIDRES 0.382426 Hz
AQ 1.3074932 sec
RG 8192
DW 19.950 usec
DE 6.50 usec
TE 299.7 K
D1 2.00000000 sec
d11 0.03000000 sec
DELTA 1.89999998 sec
MCREST 0.00000000 sec
MCWRK 0.01500000 sec
CHANNEL f1
NUC1 13C
P1 10.00 usec
PL1 5.00 dB
SFO1 100.6242995 MHz
CHANNEL f2
CPDPRG2 waltz16
NUC2 1H
PCPD2 90.00 usec
PL2 4.00 dB
PL12 20.80 dB
PL13 23.80 dB
SFO2 400.1319000 MHz
F2 - Processing parameters
SI 32768
SF 100.6127523 MHz
WDW EM
SSB 0
LB 1.00 Hz
GB 0
PC 1.40
1D NMR plot parameters
CX 20.00 cm
CY 11.49 cm
F1P 220.000 ppm
F1 22134.80 Hz
F2P -10.000 ppm
F2 -1006.13 Hz
PPMCM 11.50000 ppm/cm
HZCM 1157.04663 Hz/cm

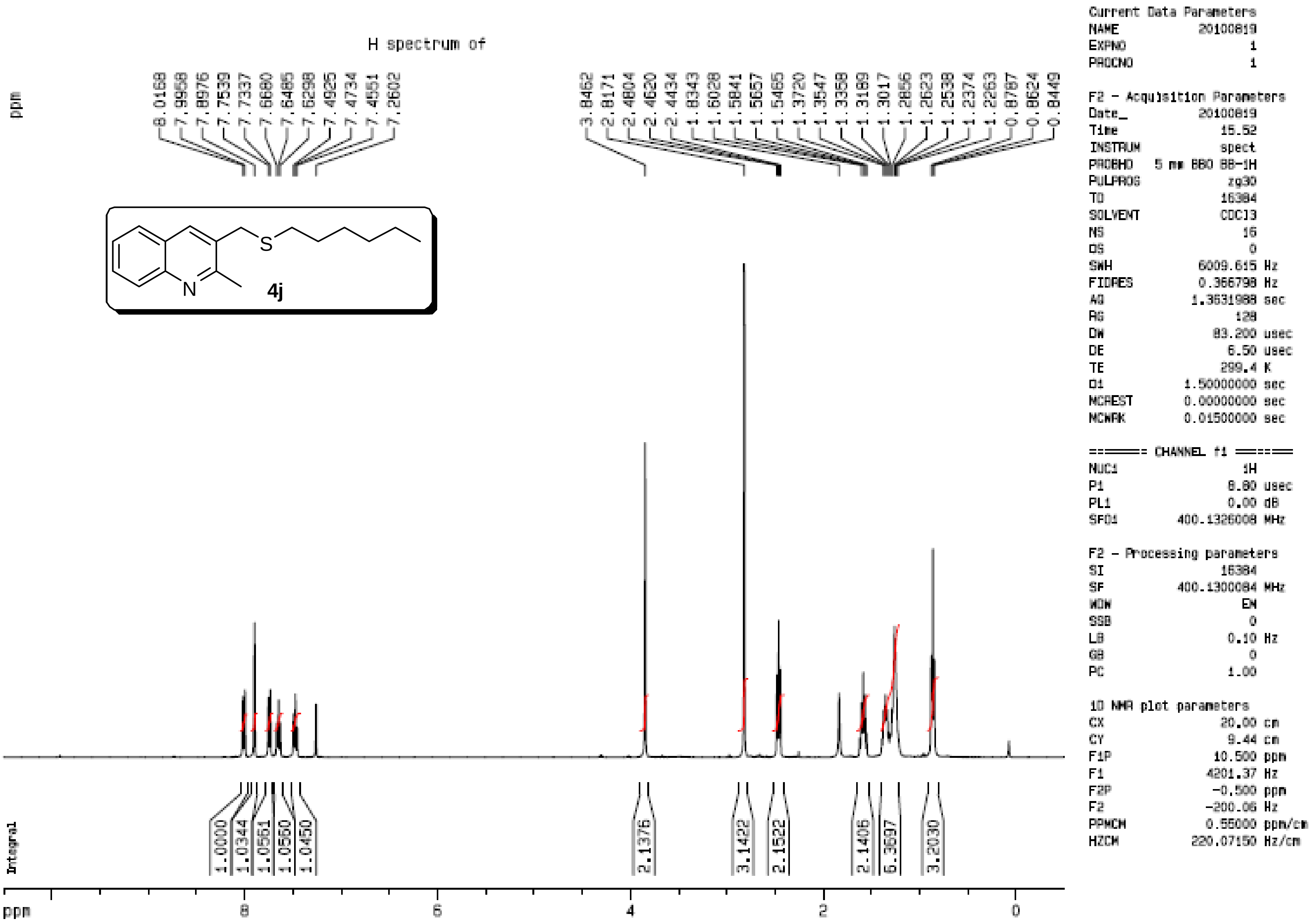
H spectrum of
4j
N
S
ppm
Integral
1.0000
1.0344
1.0561
1.0560
1.0450
2.1376
3.1422
2.1522
2.1406
6.3697
3.2030
8.0168
7.9958
7.8976
7.7539
7.7337
7.6680
7.6485
7.6298
7.4925
7.4734
7.4551
7.2602
3.8462
2.8171
2.4804
2.4620
2.4434
1.8343
1.6028
1.5841
1.5657
1.5465
1.3720
1.3547
1.3358
1.3189
1.3017
1.2856
1.2623
1.2538
1.2374
1.2263
0.8787
0.8624
0.8449
Current Data Parameters
NAME 20100819
EXPNO 1
PROCNO 1
F2 - Acquisition Parameters
Date_ 20100819
Time 15.52
INSTRUM spect
PROBHD 5 mm BBO BB-1H
PULPROG zg30
TD 16384
SOLVENT CDCl3
NS 16
DS 0
SWH 6009.615 Hz
FIDRES 0.366798 Hz
AQ 1.3631988 sec
RG 128
DW 83.200 usec
DE 6.50 usec
TE 299.4 K
D1 1.50000000 sec
MCREST 0.00000000 sec
MCWRK 0.01500000 sec
======== CHANNEL f1 ========
NUC1 1H
P1 8.80 usec
PL1 0.00 dB
SFO1 400.1326008 MHz
F2 - Processing parameters
SI 16384
SF 400.1300084 MHz
WDW EM
SSB 0
LB 0.10 Hz
GB 0
PC 1.00
1D NMR plot parameters
CX 20.00 cm
CY 9.44 cm
F1P 10.500 ppm
F1 4201.37 Hz
F2P -0.500 ppm
F2 -200.06 Hz
PPMCM 0.55000 ppm/cm
HZCM 220.07150 Hz/cm
8
6
4
2
0

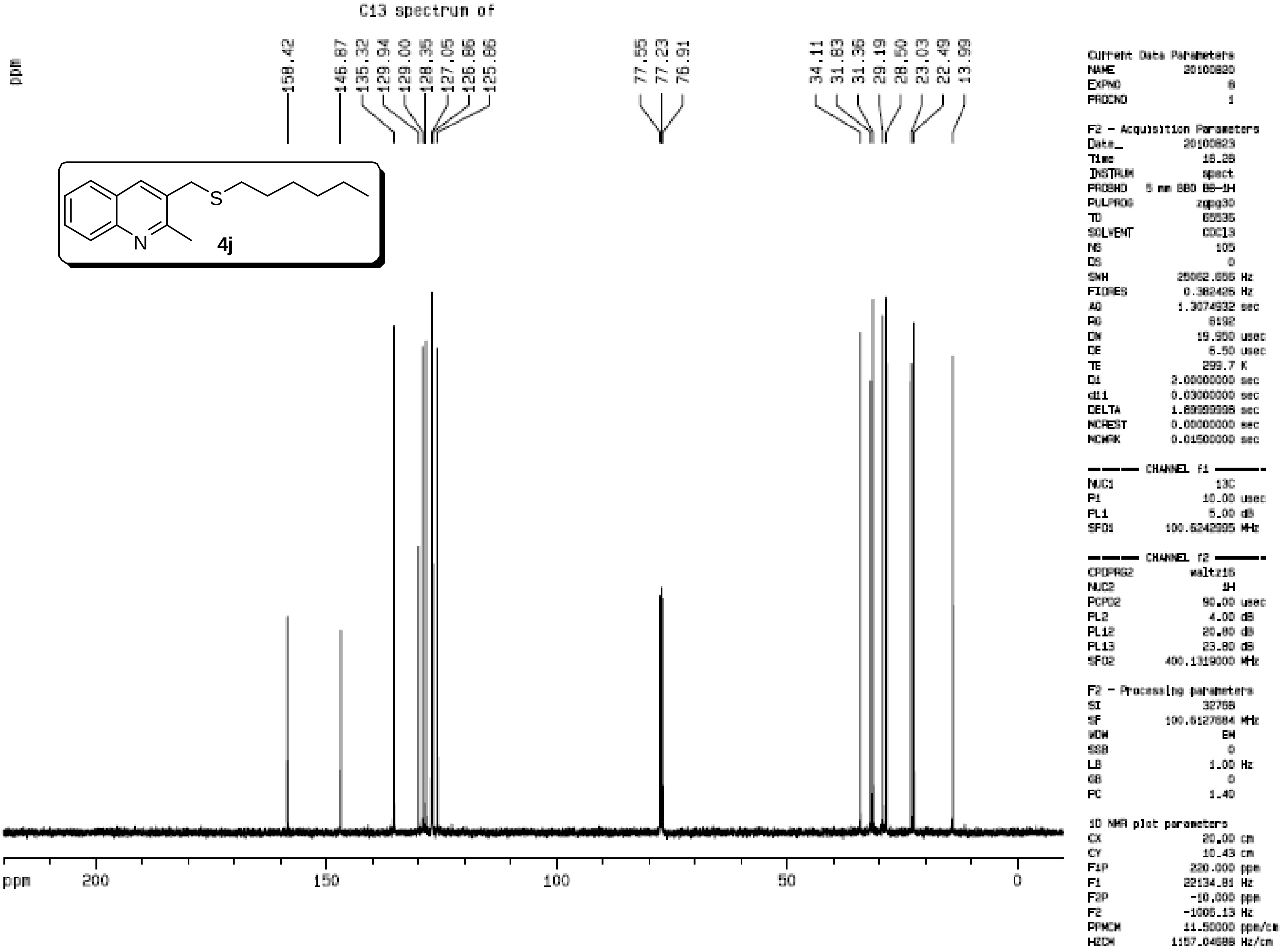
C13 spectrum of
158.42
146.87
135.32
129.94
129.00
128.35
127.05
126.86
125.86
77.55
77.23
76.91
34.11
31.83
31.36
29.19
28.50
23.03
22.49
13.99
ppm
N
S
4j
ppm 200 150 100 50 0

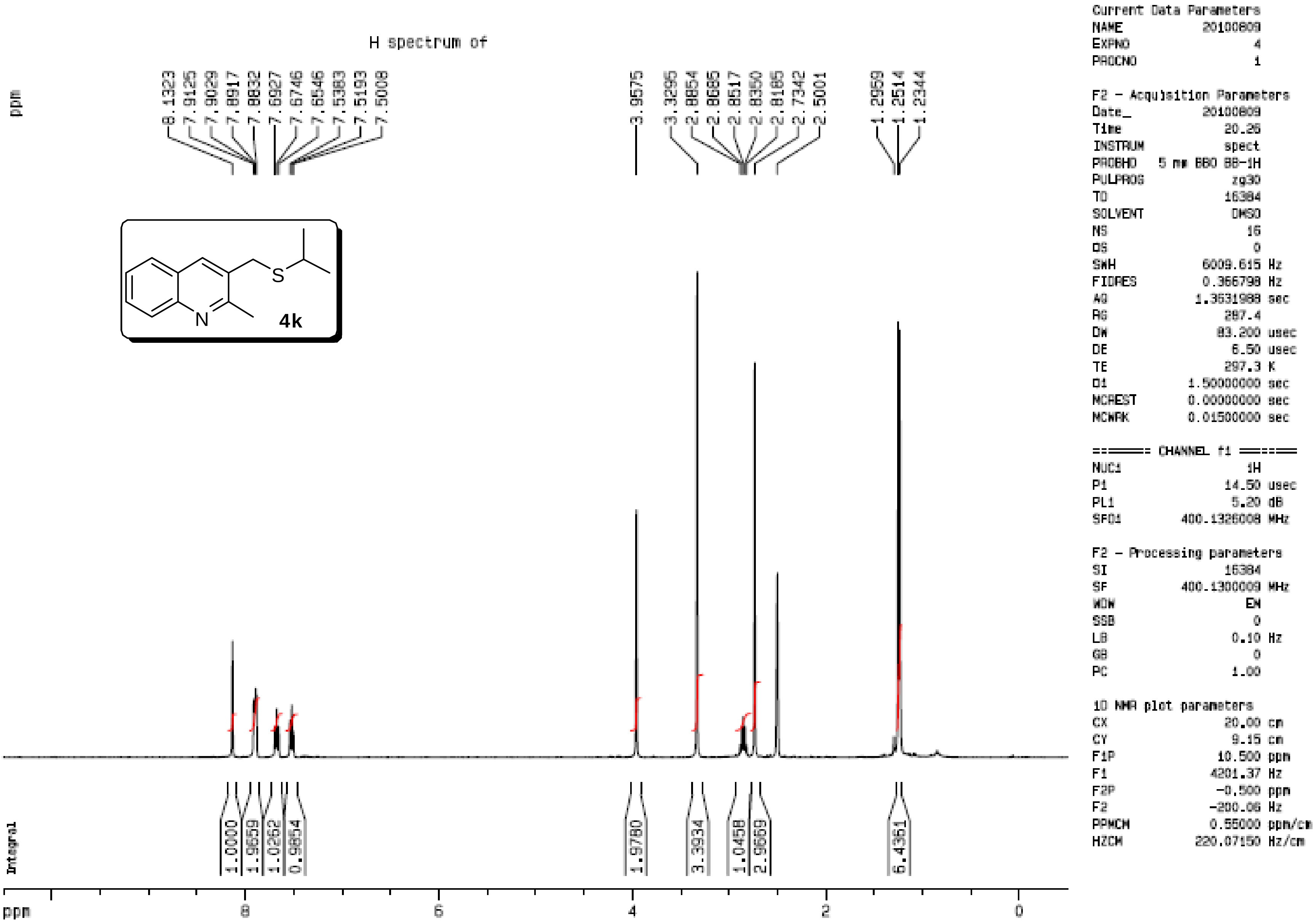
H spectrum of
ppm
8.1323
7.9125
7.9029
7.8917
7.8832
7.6927
7.6746
7.6546
7.5383
7.5193
7.5008
3.9575
3.3295
2.8854
2.8685
2.8517
2.8350
2.8165
2.7342
2.5001
1.2959
1.2514
1.2344
S
N
4k
Integral
1.0000
1.9659
1.0262
0.9854
1.9780
3.3934
1.0458
2.9669
6.4361
ppm
8
6
4
2
0
Current Data Parameters
NAME 20100809
EXPNO 4
PROCNO 1
F2 - Acquisition Parameters
Date_ 20100809
Time 20.26
INSTRUM spect
PROBHD 5 mm BBO BB-1H
PULPROG zg30
TD 16384
SOLVENT DMSO
NS 16
DS 0
SWH 6009.615 Hz
FIDRES 0.366798 Hz
AQ 1.3631988 sec
RG 287.4
DW 83.200 usec
DE 6.50 usec
TE 297.3 K
D1 1.50000000 sec
MCREST 0.00000000 sec
MCWRK 0.01500000 sec
======== CHANNEL f1 ========
NUC1 1H
P1 14.50 usec
PL1 5.20 dB
SFO1 400.1326008 MHz
F2 - Processing parameters
SI 16384
SF 400.1300009 MHz
WDW EM
SSB 0
LB 0.10 Hz
GB 0
PC 1.00
1D NMR plot parameters
CX 20.00 cm
CY 9.15 cm
F1P 10.500 ppm
F1 4201.37 Hz
F2P -0.500 ppm
F2 -200.06 Hz
PPMCM 0.55000 ppm/cm
HZCM 220.07150 Hz/cm

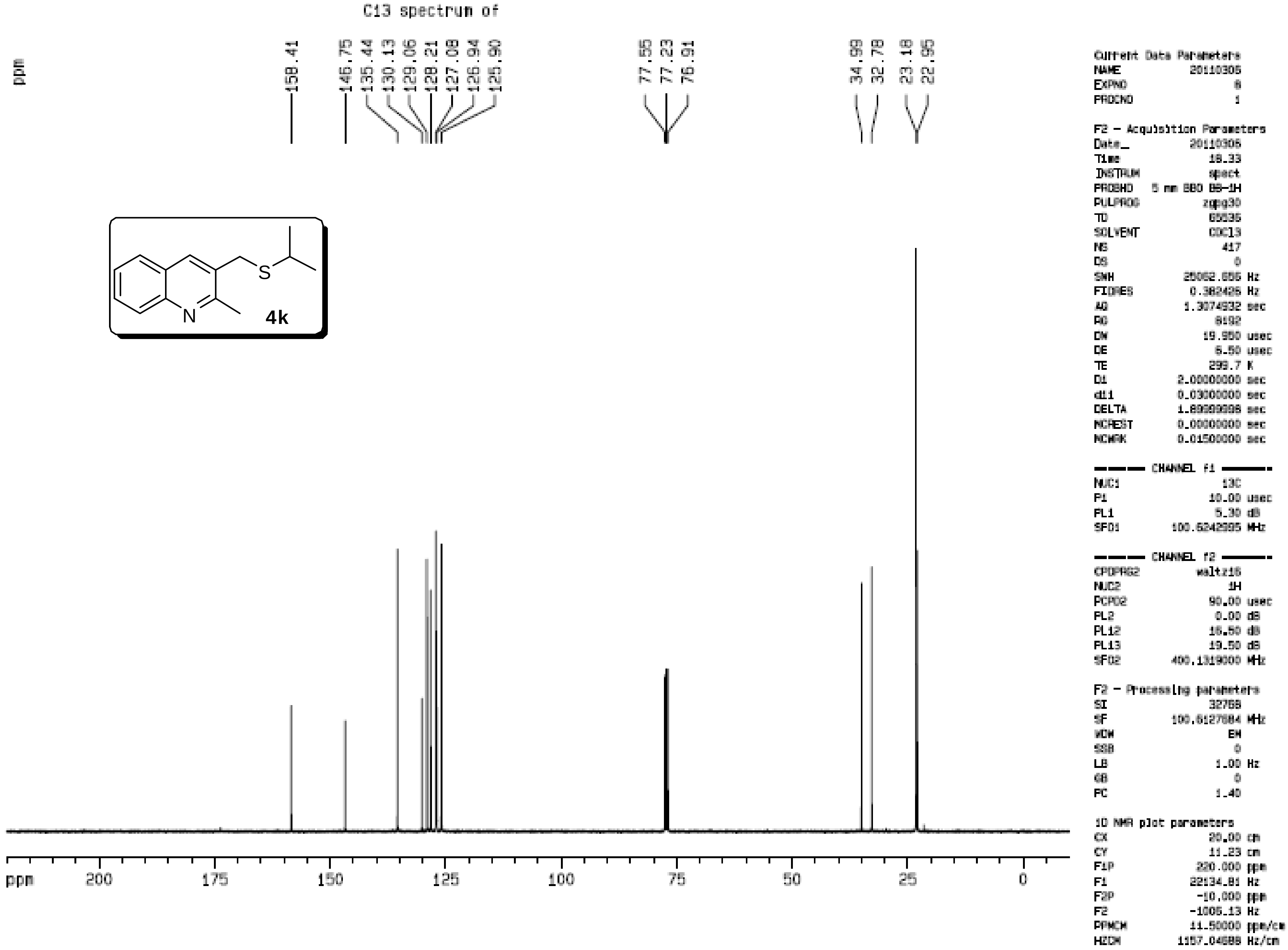

C13 spectrum of
ppm
158.41
146.75
135.44
130.13
129.06
128.21
127.08
126.94
125.90
77.55
77.23
76.91
34.99
32.78
23.18
22.95
S
N
4k
ppm 200 175 150 125 100 75 50 25 0
Current Data Parameters
NAME 20110305
EXPNO 6
PROCNO 1
F2 - Acquisition Parameters
Date_ 20110305
Time 18.33
INSTRUM spect
PROBHD 5 mm BBO BB-1H
PULPROG zgpg30
TD 65536
SOLVENT CDCl3
NS 417
DS 0
SWH 25062.656 Hz
FIDRES 0.382426 Hz
AQ 1.3074932 sec
RG 8192
DW 19.950 usec
DE 6.50 usec
TE 299.7 K
D1 2.00000000 sec
d11 0.03000000 sec
DELTA 1.89999998 sec
MCREST 0.00000000 sec
MCWRK 0.01500000 sec
CHANNEL f1
NUC1 13C
P1 10.00 usec
PL1 5.30 dB
SFO1 100.6242995 MHz
CHANNEL f2
CPDPRG2 waltz16
NUC2 1H
PCPD2 90.00 usec
PL2 0.00 dB
PL12 16.50 dB
PL13 19.50 dB
SFO2 400.1319000 MHz
F2 - Processing parameters
SI 32768
SF 100.6127684 MHz
WDW EM
SSB 0
LB 1.00 Hz
GB 0
PC 1.40
1D NMR plot parameters
CX 20.00 cm
CY 11.23 cm
F1P 220.000 ppm
F1 22134.81 Hz
F2P -10.000 ppm
F2 -1006.13 Hz
PPMCM 11.50000 ppm/cm
HZCM 1157.04688 Hz/cm

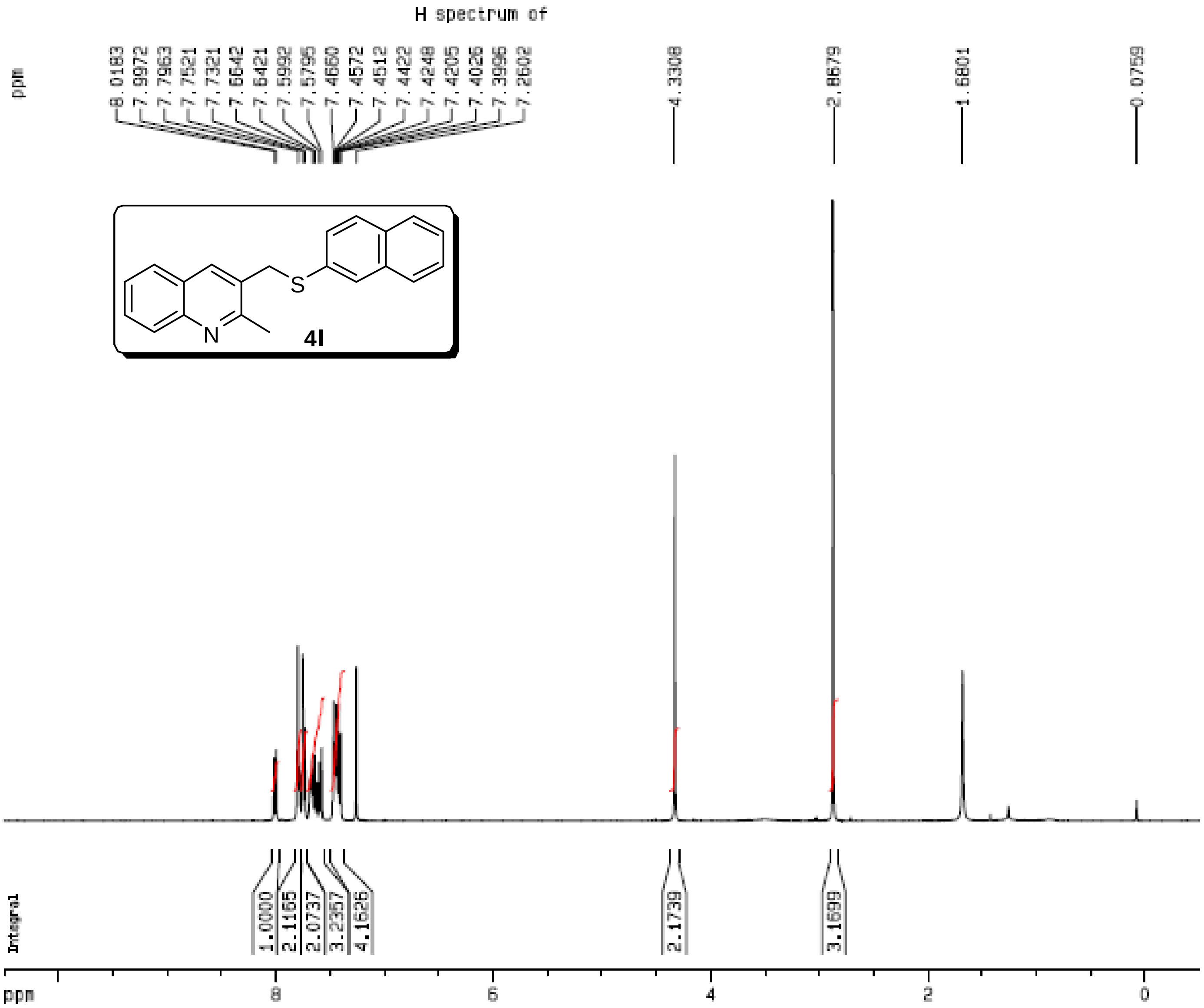

Current Data Parameters
NAME 20101012
EXPNO 1
PROCNO 1

F2 - Acquisition Parameters
Date_ 20101012
Time 16.48
INSTRUM spect
PROBHD 5 mm BBO BB-1H
PULPROG zg30
TD 16384
SOLVENT CDCl3
NS 16
DS 0
SWH 6009.615 Hz
FIDRES 0.366798 Hz
AQ 1.3631988 sec
RG 256
DW 83.200 usec
DE 6.50 usec
TE 299.5 K
D1 1.50000000 sec
MCREST 0.00000000 sec
MCWRK 0.01500000 sec

======== CHANNEL f1 ========
NUC1 1H
P1 11.20 usec
PL1 0.00 dB
SFO1 400.1326008 MHz

F2 - Processing parameters
SI 16384
SF 400.1300088 MHz
WDW EM
SSB 0
LB 0.10 Hz
GB 0
PC 1.00

1D NMR plot parameters
CX 20.00 cm
CY 10.40 cm
F1P 10.500 ppm
F1 4201.37 Hz
F2P -0.500 ppm
F2 -200.06 Hz
PPMCM 0.55000 ppm/cm
HZCM 220.07150 Hz/cm

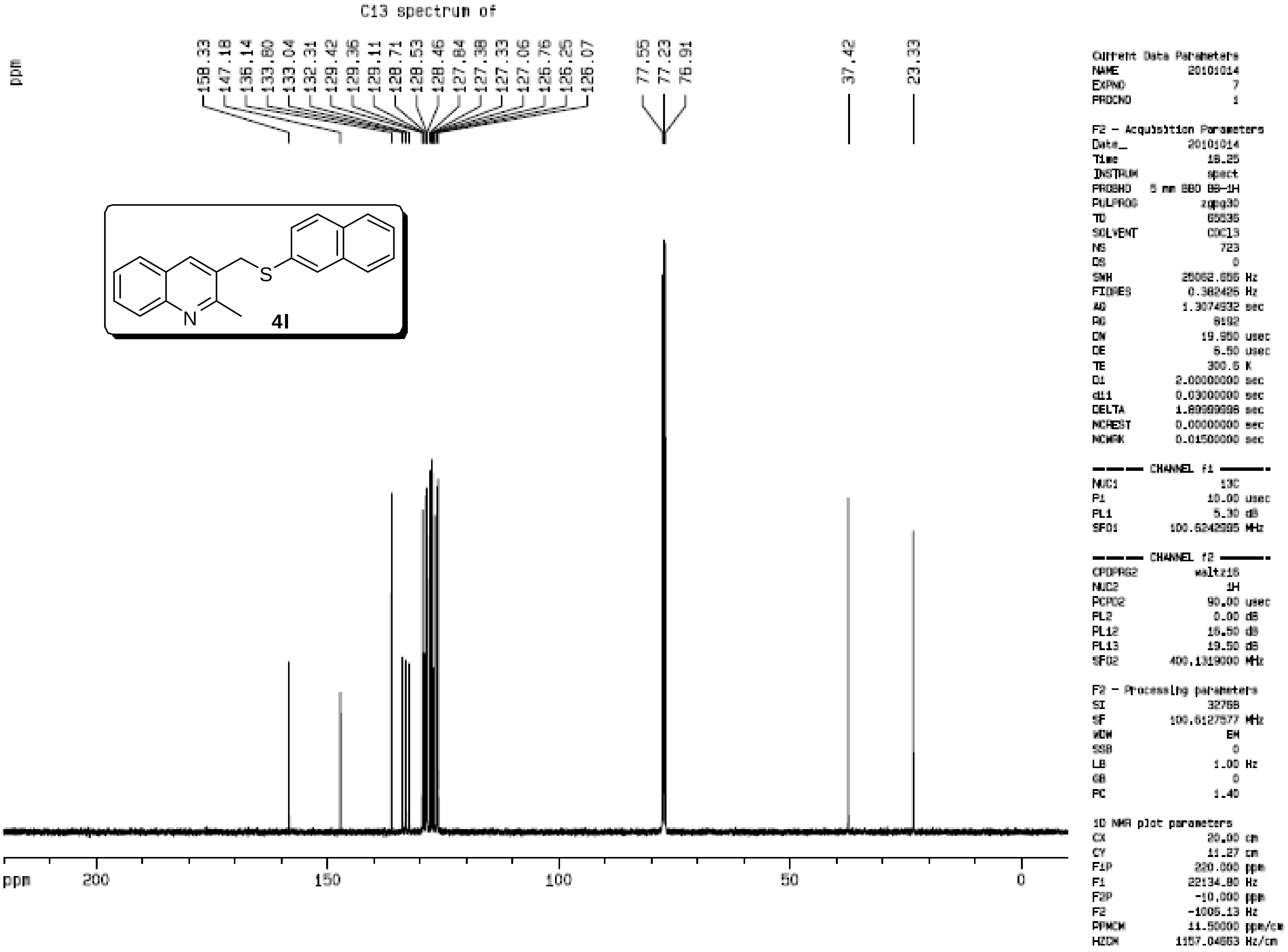
C13 spectrum of
158.33
147.18
136.14
133.80
133.04
132.31
129.42
129.36
129.11
128.71
128.53
128.46
127.84
127.38
127.33
127.06
126.76
126.25
126.07
77.55
77.23
76.91
37.42
23.33
S
N
4l
ppm
200
150
100
50
0
Current Data Parameters
NAME 20101014
EXPNO 7
PROCNO 1
F2 - Acquisition Parameters
Date_ 20101014
Time 18.25
INSTRUM spect
PROBHD 5 mm BBO BB-1H
PULPROG zgpg30
TD 65536
SOLVENT CDCl3
NS 723
DS 0
SWH 25062.656 Hz
FIDRES 0.382426 Hz
AQ 1.3074932 sec
RG 8192
DW 19.950 usec
DE 6.50 usec
TE 300.6 K
D1 2.00000000 sec
d11 0.03000000 sec
DELTA 1.89999998 sec
MCREST 0.00000000 sec
MCWRK 0.01500000 sec
CHANNEL f1
NUC1 13C
P1 10.00 usec
PL1 5.30 dB
SFO1 100.6242995 MHz
CHANNEL f2
CPDPRG2 waltz16
NUC2 1H
PCPD2 90.00 usec
PL2 0.00 dB
PL12 16.50 dB
PL13 19.50 dB
SFO2 400.1319000 MHz
F2 - Processing parameters
SI 32768
SF 100.6127577 MHz
WDW EM
SSB 0
LB 1.00 Hz
GB 0
PC 1.40
1D NMR plot parameters
CX 20.00 cm
CY 11.27 cm
F1P 220.000 ppm
F1 22134.80 Hz
F2P -10.000 ppm
F2 -1006.13 Hz
PPMCM 11.50000 ppm/cm
HZCM 1157.04663 Hz/cm

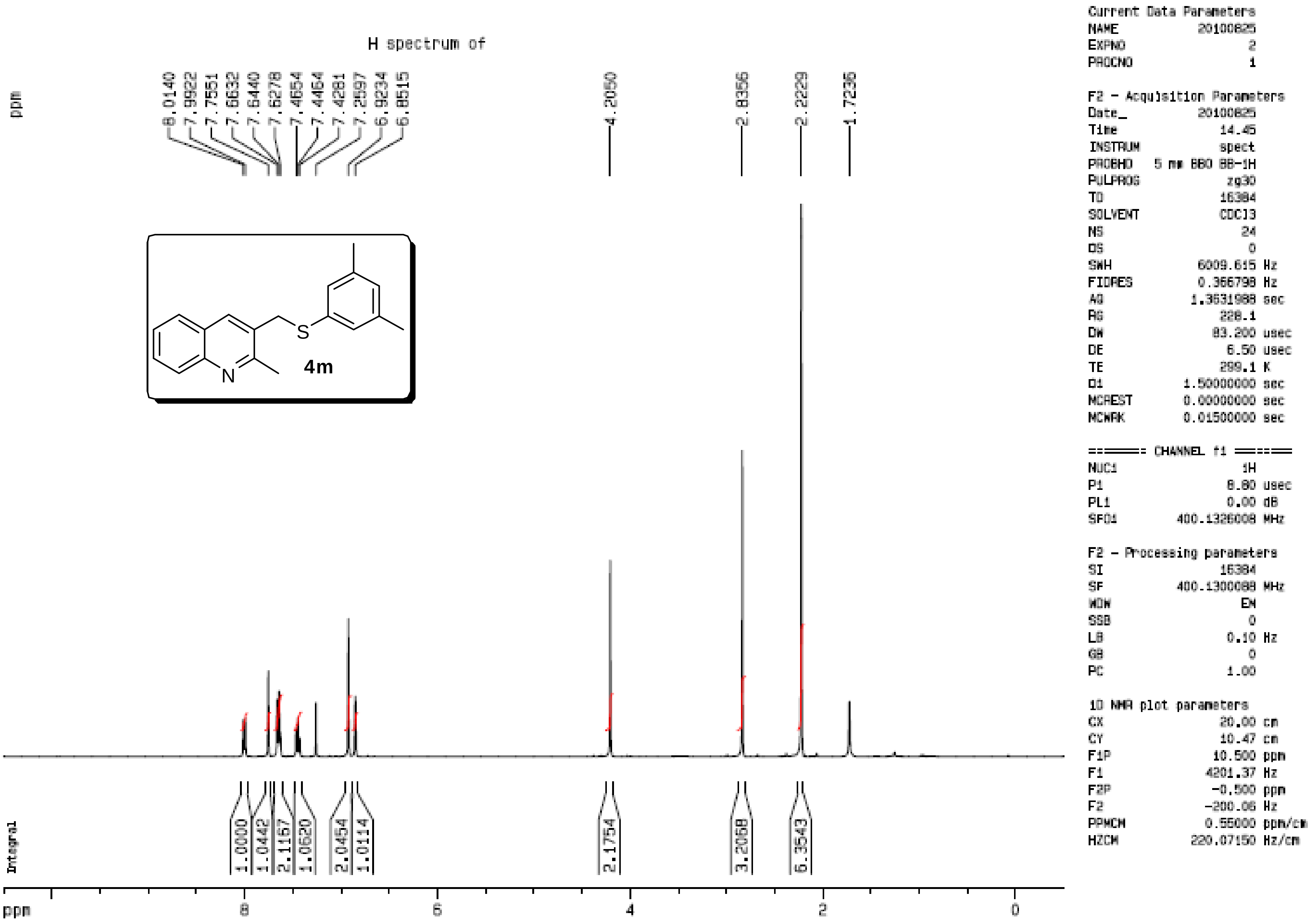
H spectrum of
4m
N
S
8.0140
7.9922
7.7551
7.6632
7.6440
7.6278
7.4654
7.4464
7.4281
7.2597
6.9234
6.8515
4.2050
2.8356
2.2229
1.7236
1.0000
1.0442
2.1157
1.0620
2.0454
1.0114
2.1754
3.2068
6.3543
Integral
ppm
8
6
4
2
0
Current Data Parameters
NAME 20100825
EXPNO 2
PROCNO 1
F2 - Acquisition Parameters
Date_ 20100825
Time 14.45
INSTRUM spect
PROBHD 5 mm BBO BB-1H
PULPROG zg30
TD 16384
SOLVENT CDCl3
NS 24
DS 0
SWH 6009.615 Hz
FIDRES 0.366798 Hz
AQ 1.3631988 sec
RG 228.1
DW 83.200 usec
DE 6.50 usec
TE 299.1 K
D1 1.50000000 sec
MCREST 0.00000000 sec
MCWRK 0.01500000 sec
======== CHANNEL f1 ========
NUC1 1H
P1 8.80 usec
PL1 0.00 dB
SFO1 400.1326008 MHz
F2 - Processing parameters
SI 16384
SF 400.1300088 MHz
WDW EM
SSB 0
LB 0.10 Hz
GB 0
PC 1.00
1D NMR plot parameters
CX 20.00 cm
CY 10.47 cm
F1P 10.500 ppm
F1 4201.37 Hz
F2P -0.500 ppm
F2 -200.06 Hz
PPMCM 0.55000 ppm/cm
HZCM 220.07150 Hz/cm

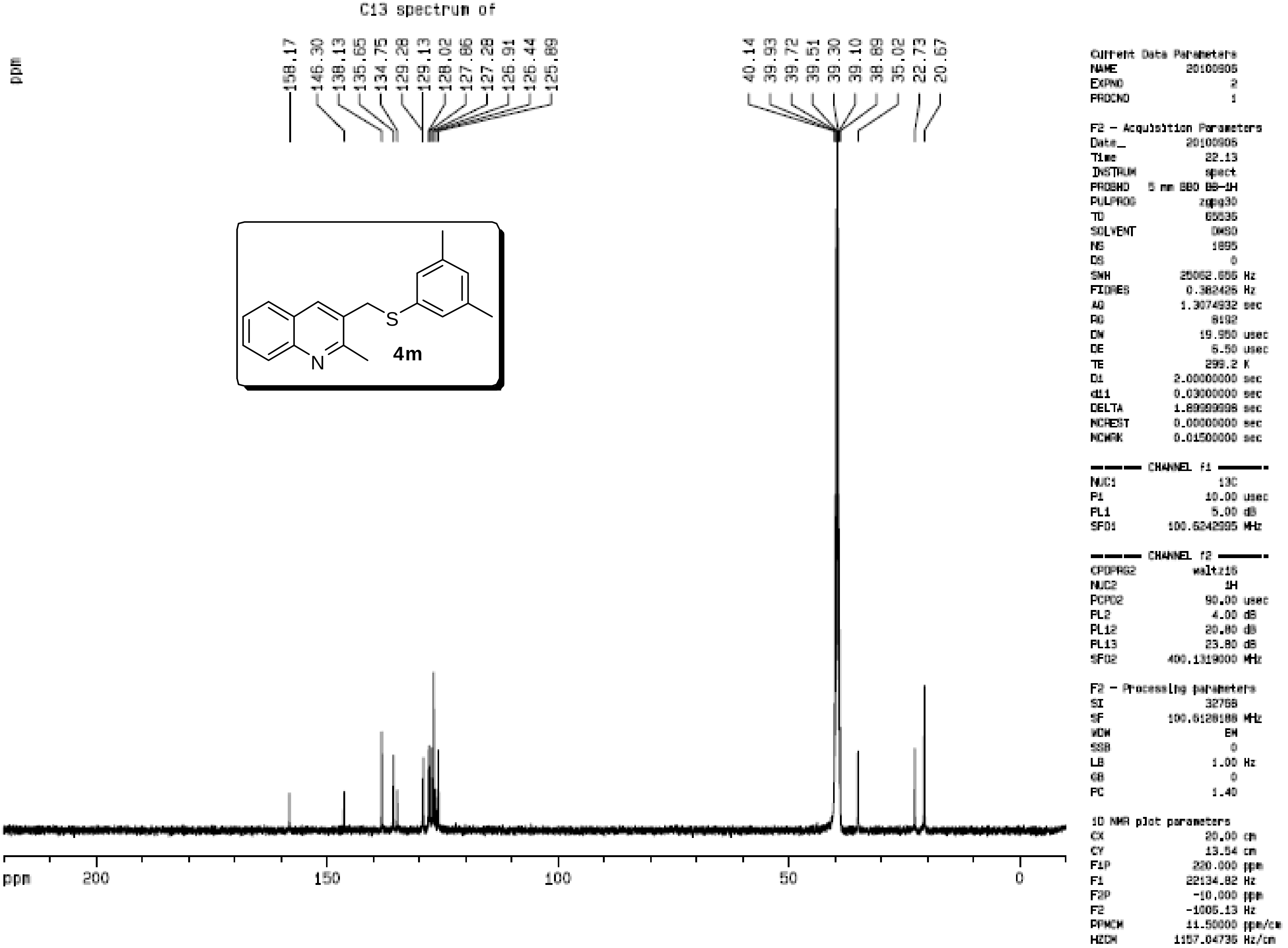
C13 spectrum of
158.17
146.30
138.13
135.65
134.75
129.28
129.13
128.02
127.86
127.28
126.91
126.44
125.89
40.14
39.93
39.72
39.51
39.30
39.10
38.89
35.02
22.73
20.67
S
N
4m
ppm 200 150 100 50 0
Current Data Parameters
NAME 20100906
EXPNO 2
PROCNO 1
F2 - Acquisition Parameters
Date_ 20100906
Time 22.13
INSTRUM spect
PROBHD 5 mm BBO BB-1H
PULPROG zgpg30
TD 65536
SOLVENT DMSO
NS 1895
DS 0
SWH 25062.656 Hz
FIDRES 0.382426 Hz
AQ 1.3074932 sec
RG 8192
DW 19.950 usec
DE 6.50 usec
TE 298.2 K
D1 2.00000000 sec
d11 0.03000000 sec
DELTA 1.89999998 sec
MCREST 0.00000000 sec
MCWRK 0.01500000 sec
CHANNEL f1
NUC1 13C
P1 10.00 usec
PL1 5.00 dB
SFO1 100.6242995 MHz
CHANNEL f2
CPDPRG2 waltz16
NUC2 1H
PCPD2 90.00 usec
PL2 4.00 dB
PL12 20.80 dB
PL13 23.80 dB
SFO2 400.1319000 MHz
F2 - Processing parameters
SI 32768
SF 100.6128188 MHz
WDW EM
SSB 0
LB 1.00 Hz
GB 0
PC 1.40
1D NMR plot parameters
CX 20.00 cm
CY 13.54 cm
F1P 220.000 ppm
F1 22134.82 Hz
F2P -10.000 ppm
F2 -1006.13 Hz
PPMCM 11.50000 ppm/cm
HZCM 1157.04736 Hz/cm

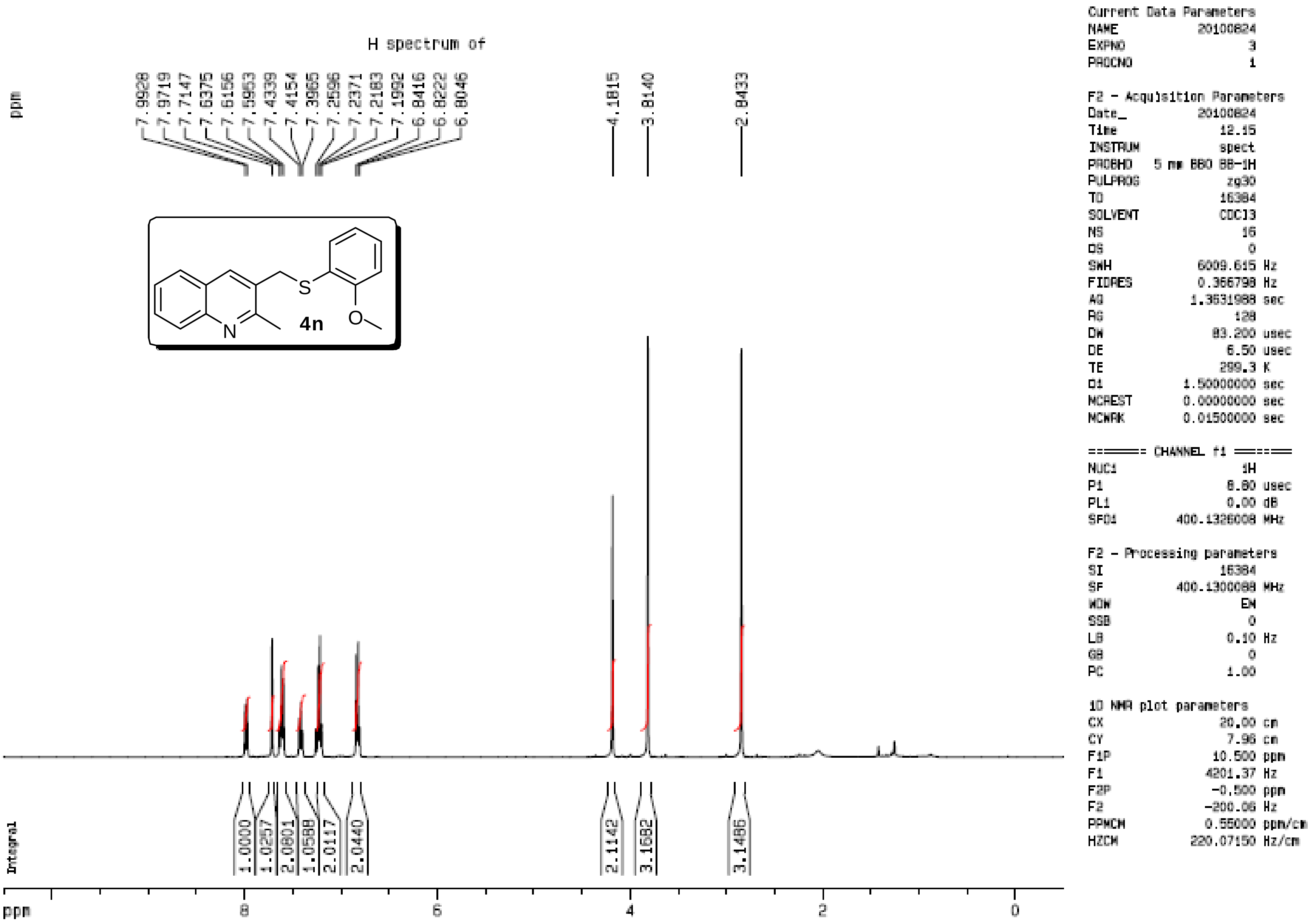

H spectrum of
4n
7.9928
7.9719
7.7147
7.6375
7.6156
7.5953
7.4339
7.4154
7.3965
7.2596
7.2371
7.2183
7.1992
6.8416
6.8222
6.8046
4.1815
3.8140
2.8433
1.0000
1.0257
2.0801
1.0588
2.0117
2.0440
2.1142
3.1682
3.1486
Integral
ppm
8
6
4
2
0
Current Data Parameters
NAME 20100824
EXPNO 3
PROCNO 1
F2 - Acquisition Parameters
Date_ 20100824
Time 12.15
INSTRUM spect
PROBHD 5 mm BBO BB-1H
PULPROG zg30
TD 16384
SOLVENT CDCl3
NS 16
DS 0
SWH 6009.615 Hz
FIDRES 0.366798 Hz
AQ 1.3631988 sec
RG 128
DW 83.200 usec
DE 6.50 usec
TE 299.3 K
D1 1.50000000 sec
MCREST 0.00000000 sec
MCWRK 0.01500000 sec
======= CHANNEL f1 =======
NUC1 1H
P1 8.80 usec
PL1 0.00 dB
SFO1 400.1326008 MHz
F2 - Processing parameters
SI 16384
SF 400.1300088 MHz
WDW EM
SSB 0
LB 0.10 Hz
GB 0
PC 1.00
1D NMR plot parameters
CX 20.00 cm
CY 7.96 cm
F1P 10.500 ppm
F1 4201.37 Hz
F2P -0.500 ppm
F2 -200.06 Hz
PPMCM 0.55000 ppm/cm
HZCM 220.07150 Hz/cm

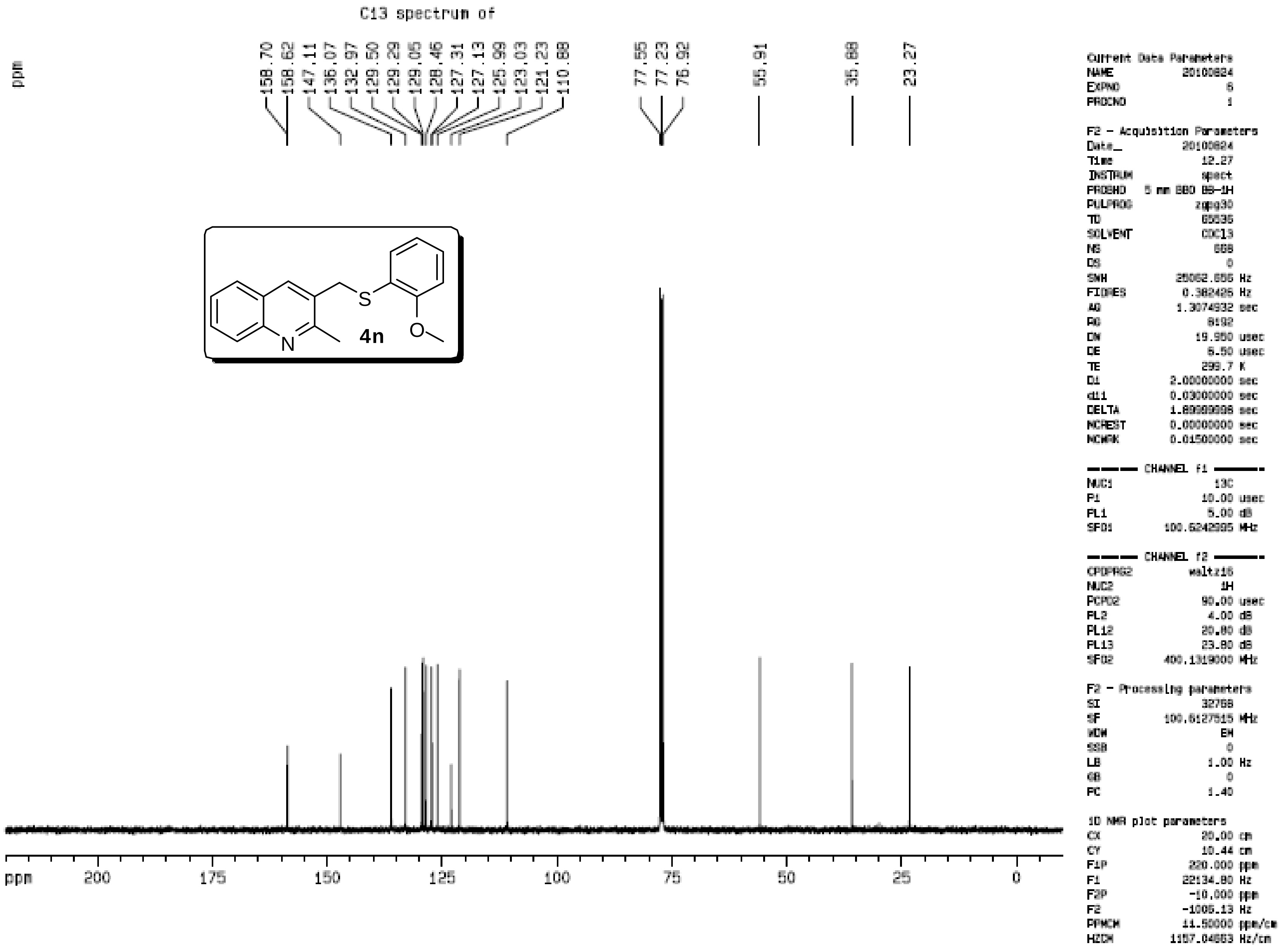
C13 spectrum of
158.70
158.62
147.11
136.07
132.97
129.50
129.29
129.05
128.46
127.31
127.13
125.99
123.03
121.23
110.88
77.55
77.23
76.92
55.91
35.88
23.27
4n
N
S
O
ppm
200
175
150
125
100
75
50
25
0

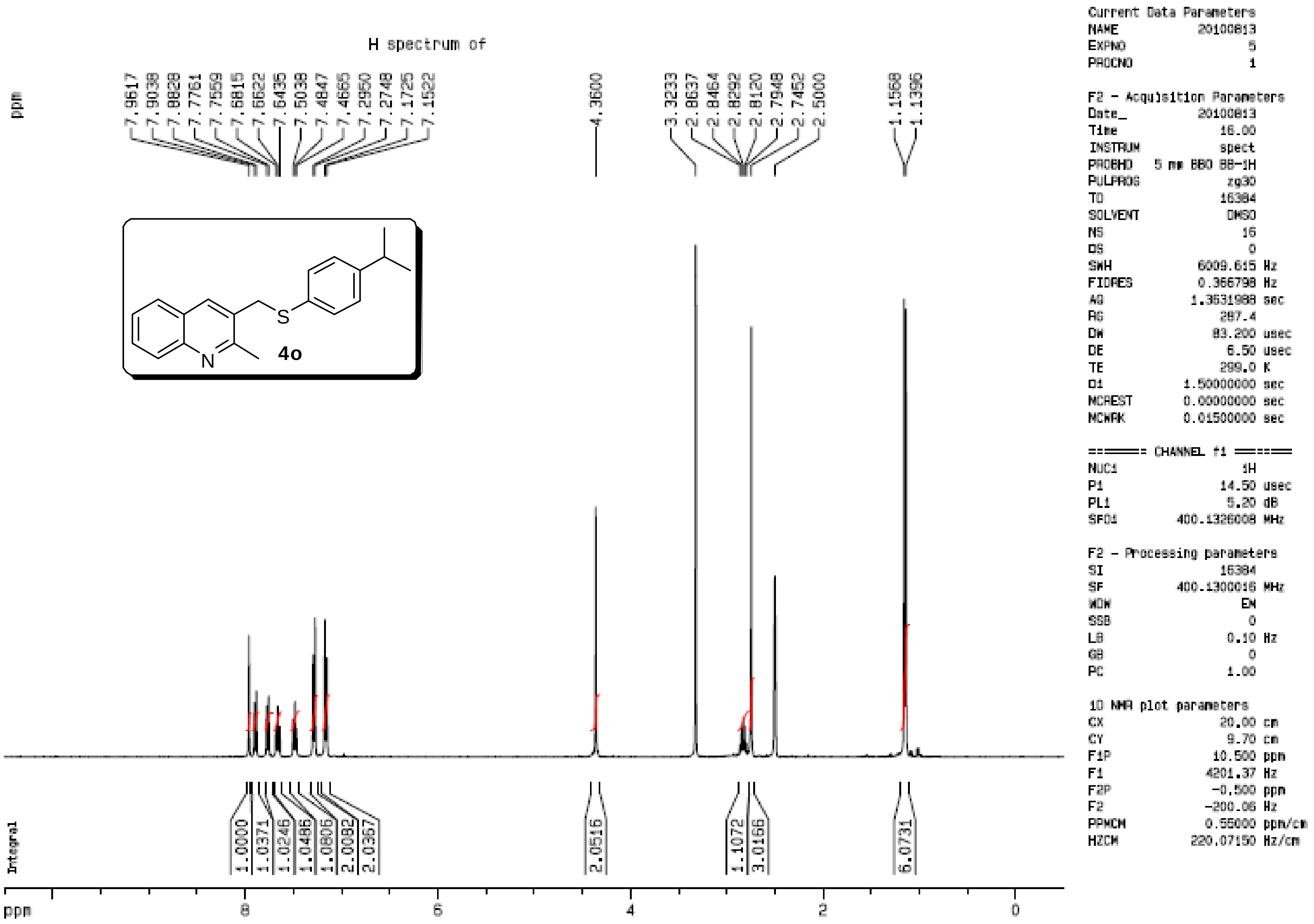
H spectrum of
ppm
7.9617
7.9038
7.8828
7.7761
7.7559
7.6815
7.6622
7.6435
7.5038
7.4847
7.4665
7.2950
7.2748
7.1725
7.1522
4.3600
3.3233
2.8637
2.8464
2.8292
2.8120
2.7948
2.7452
2.5000
1.1568
1.1396
S
N
4o
Integral
1.0000
1.0371
1.0246
1.0486
1.0806
2.0082
2.0367
2.0516
1.1072
3.0166
6.0731
ppm
8
6
4
2
0
Current Data Parameters
NAME 20100813
EXPNO 5
PROCNO 1
F2 - Acquisition Parameters
Date_ 20100813
Time 16.00
INSTRUM spect
PROBHD 5 mm BBO BB-1H
PULPROG zg30
TD 16384
SOLVENT DMSO
NS 16
DS 0
SWH 6009.615 Hz
FIDRES 0.366798 Hz
AQ 1.3631988 sec
RG 287.4
DW 83.200 usec
DE 6.50 usec
TE 299.0 K
D1 1.50000000 sec
MCREST 0.00000000 sec
MCWRK 0.01500000 sec
======== CHANNEL f1 ========
NUC1 1H
P1 14.50 usec
PL1 5.20 dB
SFO1 400.1326008 MHz
F2 - Processing parameters
SI 16384
SF 400.1300016 MHz
WDW EM
SSB 0
LB 0.10 Hz
GB 0
PC 1.00
1D NMR plot parameters
CX 20.00 cm
CY 9.70 cm
F1P 10.500 ppm
F1 4201.37 Hz
F2P -0.500 ppm
F2 -200.06 Hz
PPMCM 0.55000 ppm/cm
HZCM 220.07150 Hz/cm

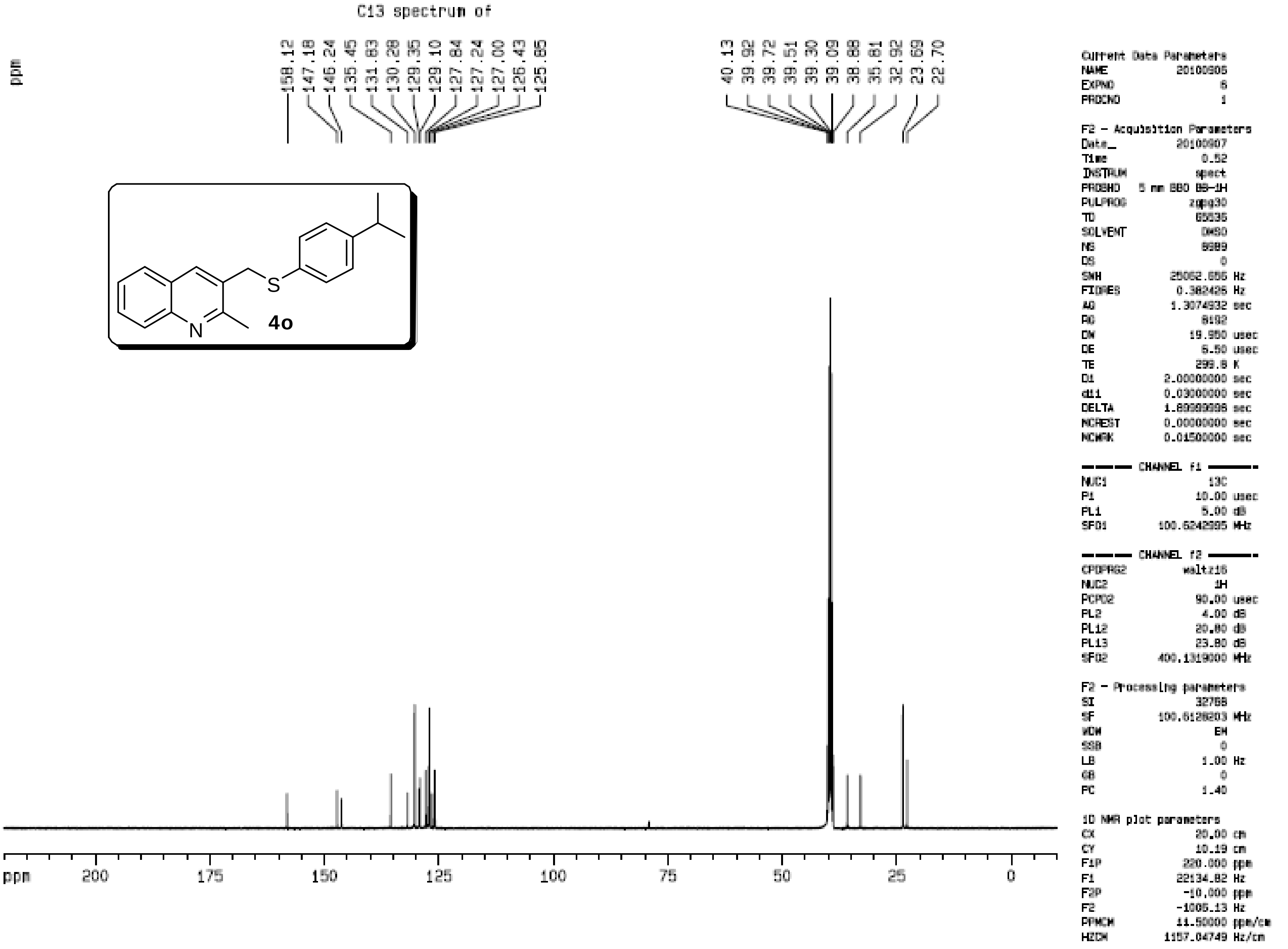
C13 spectrum of
158.12
147.18
146.24
135.45
131.63
130.28
129.35
129.10
127.84
127.24
127.00
126.43
125.85
40.13
39.92
39.72
39.51
39.30
39.09
38.88
35.61
32.92
23.69
22.70
4o
ppm
200
175
150
125
100
75
50
25
0

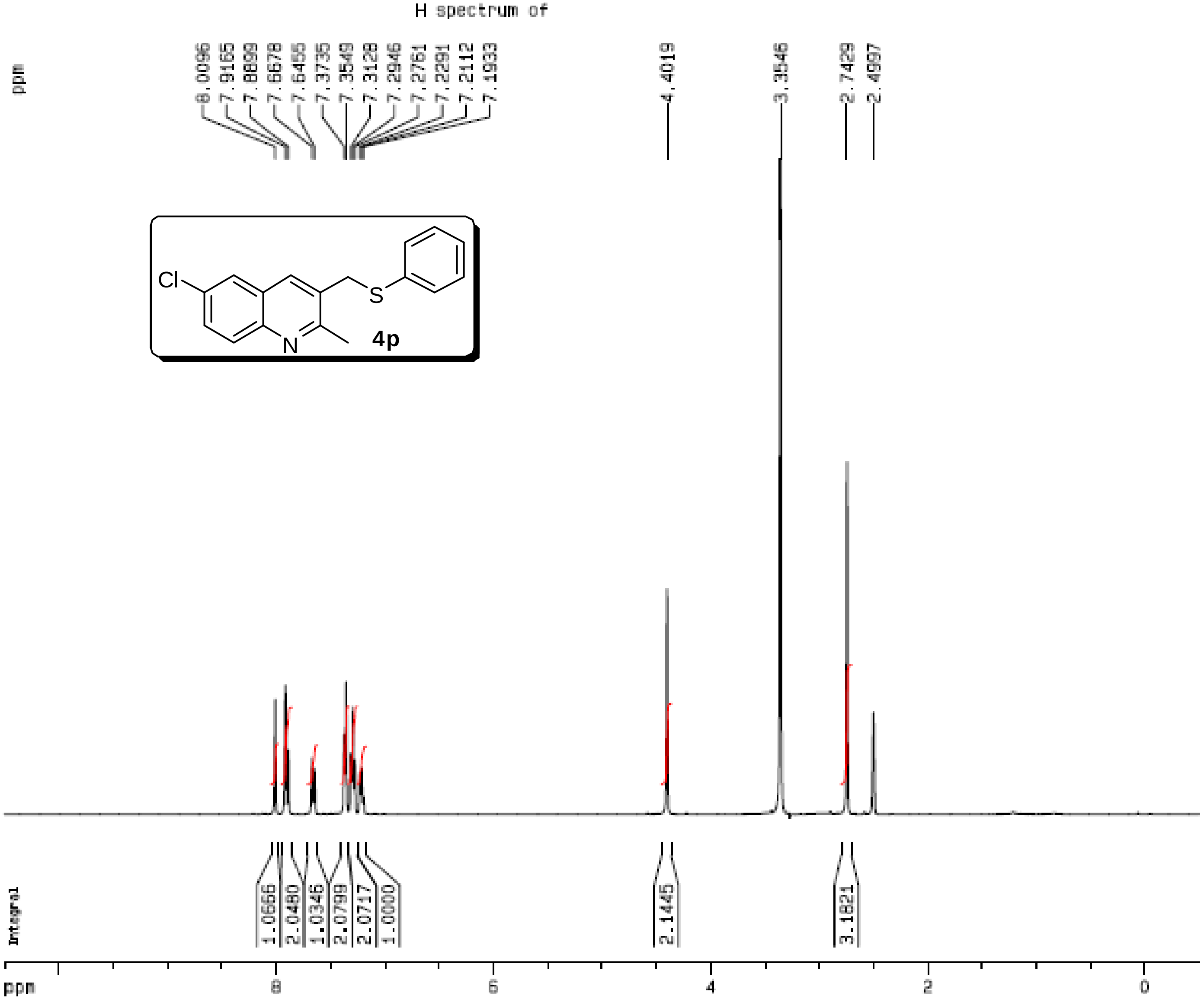


```
Current Data Parameters
NAME              20101006
EXPNO                    1
PROCNO                   1

F2 - Acquisition Parameters
Date_             20101006
Time                 12.05
INSTRUM              spect
PROBHD    5 mm BBO BB-1H
PULPROG               zg30
TD                   16384
SOLVENT               DMSO
NS                      36
DS                       0
SWH              6009.615 Hz
FIDRES           0.366798 Hz
AQ              1.3631988 sec
RG                     128
DW                  83.200 usec
DE                    6.50 usec
TE                   300.2 K
D1              1.50000000 sec
MCREST          0.00000000 sec
MCWRK           0.01500000 sec

======== CHANNEL f1 ========
NUC1                    1H
P1                   11.20 usec
PL1                   0.00 dB
SFO1           400.1326008 MHz

F2 - Processing parameters
SI                   16384
SF             400.1300016 MHz
WDW                     EM
SSB                      0
LB                    0.10 Hz
GB                       0
PC                    1.00

1D NMR plot parameters
CX                   20.00 cm
CY                   22.34 cm
F1P                 10.500 ppm
F1                 4201.37 Hz
F2P                 -0.500 ppm
F2                 -200.06 Hz
PPMCM              0.55000 ppm/cm
HZCM             220.07150 Hz/cm
```

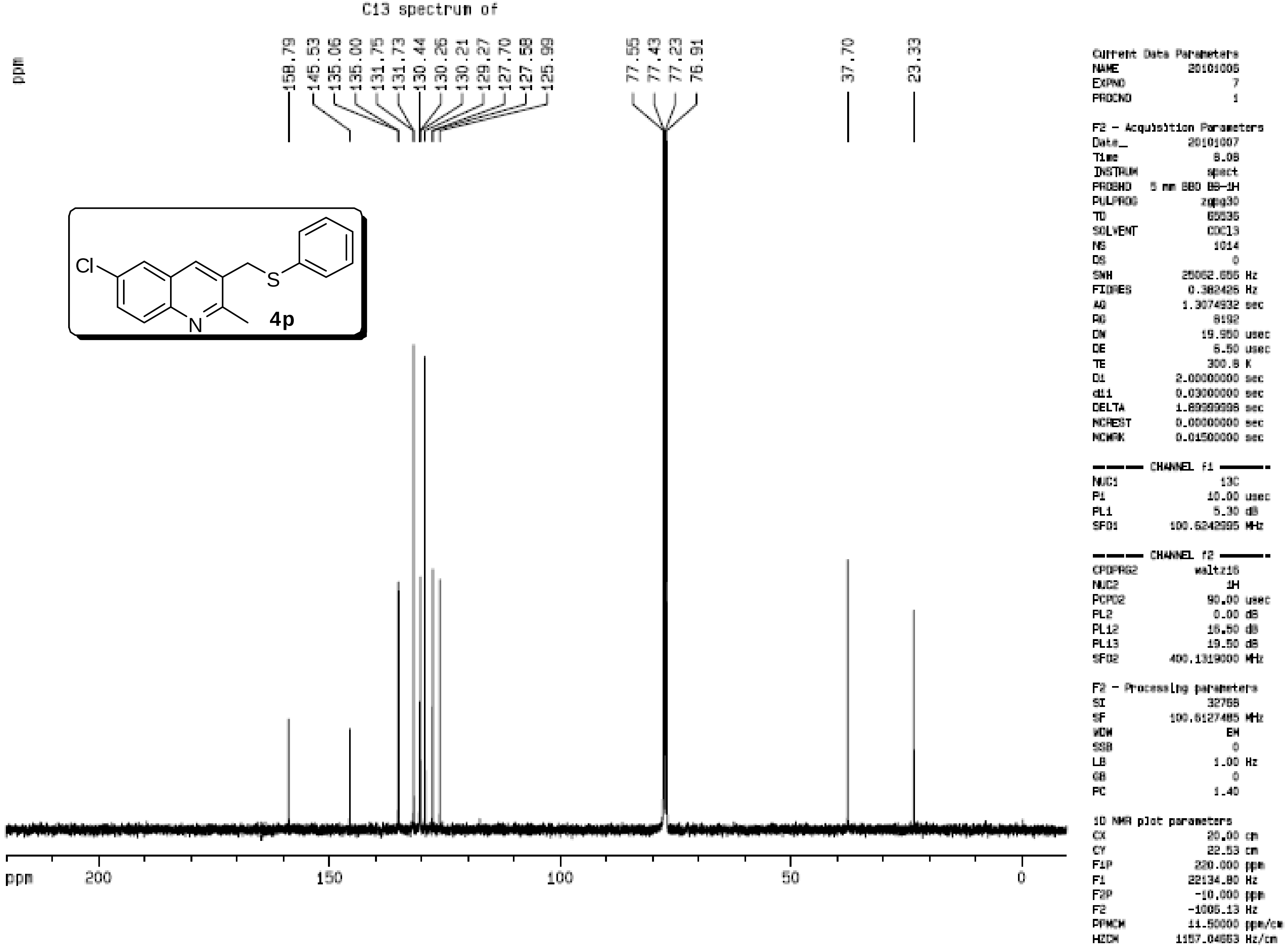

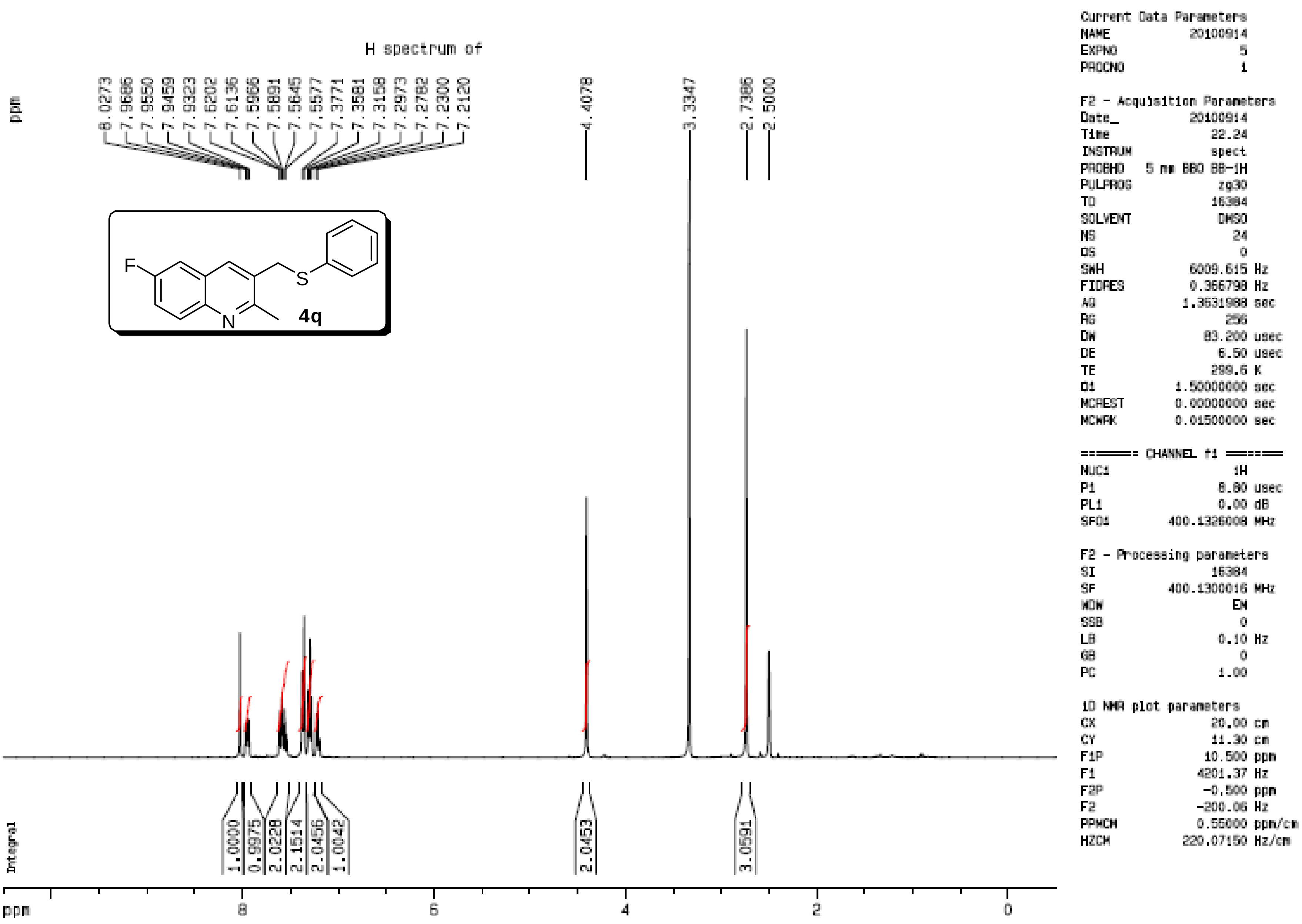
H spectrum of
4q
Current Data Parameters
NAME 20100914
EXPNO 5
PROCNO 1
F2 - Acquisition Parameters
Date_ 20100914
Time 22.24
INSTRUM spect
PROBHD 5 mm BBO BB-1H
PULPROG zg30
TD 16384
SOLVENT DMSO
NS 24
DS 0
SWH 6009.615 Hz
FIDRES 0.366798 Hz
AQ 1.3631988 sec
RG 256
DW 83.200 usec
DE 6.50 usec
TE 299.6 K
D1 1.50000000 sec
MCREST 0.00000000 sec
MCWRK 0.01500000 sec
======== CHANNEL f1 ========
NUC1 1H
P1 8.80 usec
PL1 0.00 dB
SFO1 400.1326008 MHz
F2 - Processing parameters
SI 16384
SF 400.1300016 MHz
WDW EM
SSB 0
LB 0.10 Hz
GB 0
PC 1.00
1D NMR plot parameters
CX 20.00 cm
CY 11.30 cm
F1P 10.500 ppm
F1 4201.37 Hz
F2P -0.500 ppm
F2 -200.06 Hz
PPMCM 0.55000 ppm/cm
HZCM 220.07150 Hz/cm

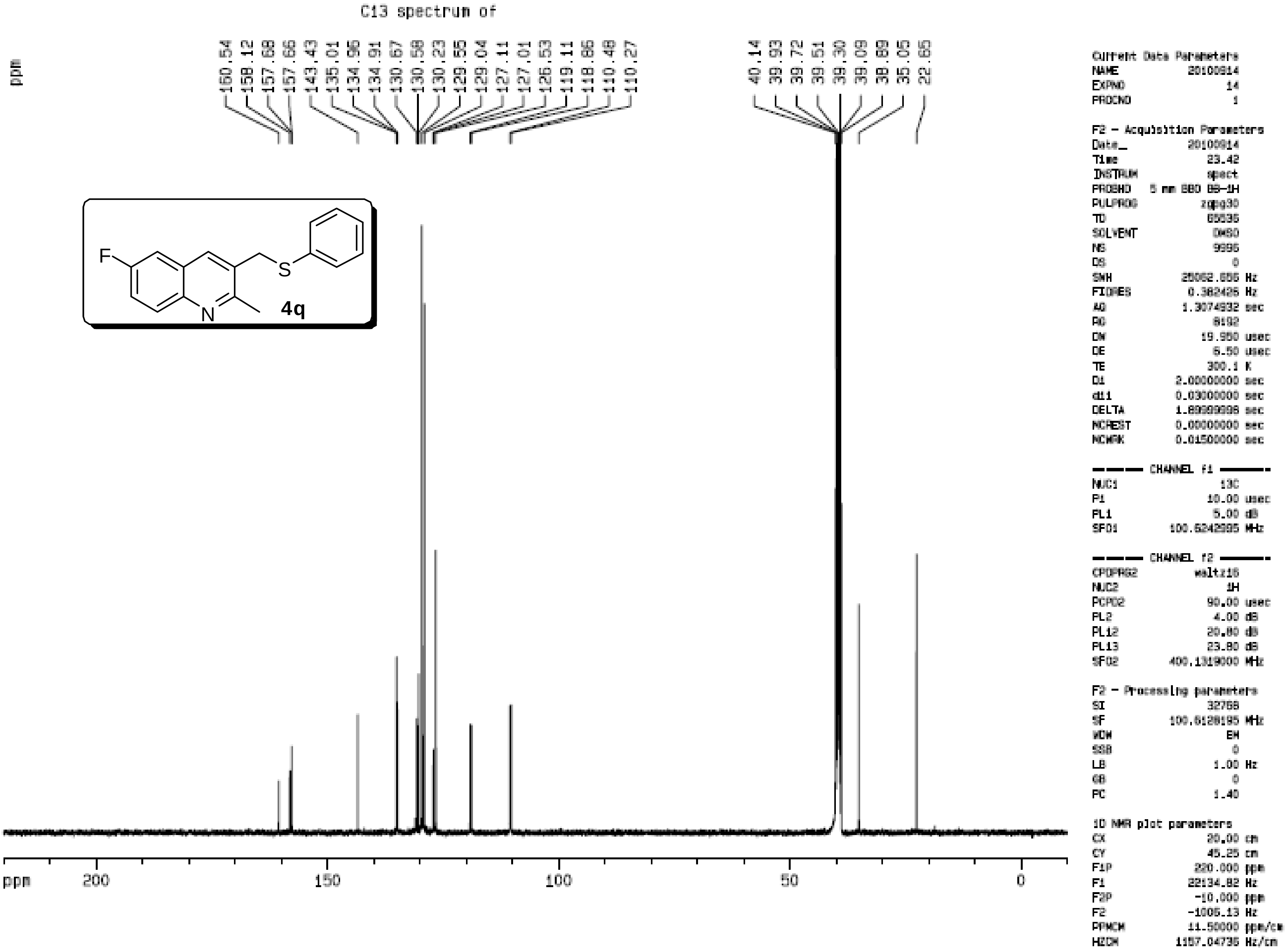
C13 spectrum of
ppm
160.54
158.12
157.68
157.66
143.43
135.01
134.96
134.91
130.67
130.58
130.23
129.55
129.04
127.11
127.01
126.53
119.11
118.86
110.48
110.27
40.14
39.93
39.72
39.51
39.30
39.09
38.89
35.05
22.65
F
S
N
4q
ppm 200 150 100 50 0
Current Data Parameters
NAME 20100914
EXPNO 14
PROCNO 1
F2 - Acquisition Parameters
Date_ 20100914
Time 23.42
INSTRUM spect
PROBHD 5 mm BBO BB-1H
PULPROG zgpg30
TD 65536
SOLVENT DMSO
NS 9996
DS 0
SWH 25062.656 Hz
FIDRES 0.382426 Hz
AQ 1.3074932 sec
RG 8192
DW 19.950 usec
DE 6.50 usec
TE 300.1 K
D1 2.00000000 sec
d11 0.03000000 sec
DELTA 1.89999998 sec
MCREST 0.00000000 sec
MCWRK 0.01500000 sec
CHANNEL f1
NUC1 13C
P1 10.00 usec
PL1 5.00 dB
SFO1 100.6242995 MHz
CHANNEL f2
CPDPRG2 waltz16
NUC2 1H
PCPD2 90.00 usec
PL2 4.00 dB
PL12 20.80 dB
PL13 23.80 dB
SFO2 400.1319000 MHz
F2 - Processing parameters
SI 32768
SF 100.6128195 MHz
WDW EM
SSB 0
LB 1.00 Hz
GB 0
PC 1.40
1D NMR plot parameters
CX 20.00 cm
CY 45.25 cm
F1P 220.000 ppm
F1 22134.82 Hz
F2P -10.000 ppm
F2 -1006.13 Hz
PPMCM 11.50000 ppm/cm
HZCM 1157.04736 Hz/cm

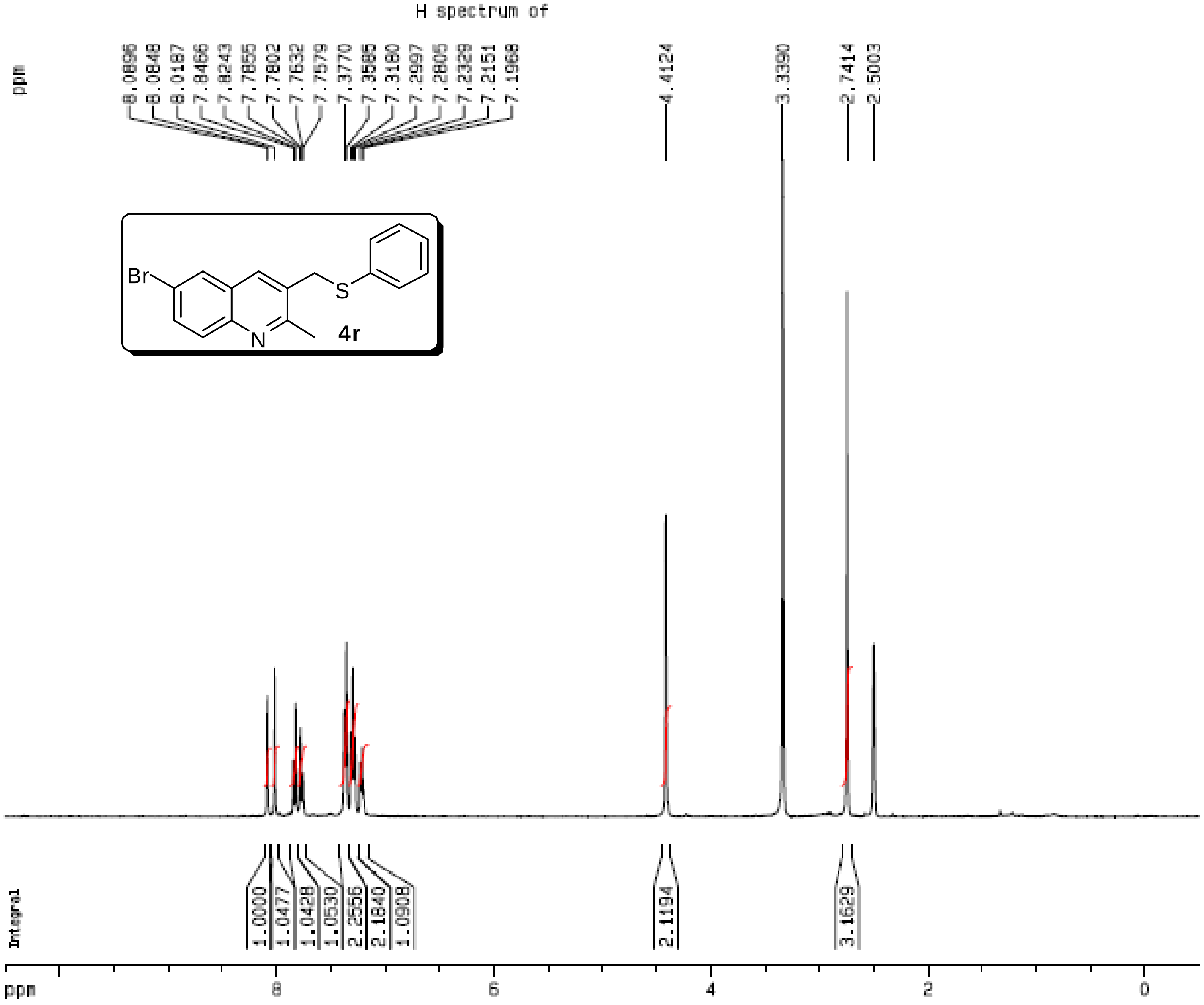

Current Data Parameters
NAME 20110216
EXPNO 5
PROCNO 1

F2 - Acquisition Parameters
Date_ 20110216
Time 20.27
INSTRUM spect
PROBHD 5 mm BBO BB-1H
PULPROG zg30
TD 16384
SOLVENT DMSO
NS 24
DS 0
SWH 6009.615 Hz
FIDRES 0.366798 Hz
AQ 1.3631988 sec
RG 287.4
DW 83.200 usec
DE 6.50 usec
TE 297.2 K
D1 1.50000000 sec
MCREST 0.00000000 sec
MCWRK 0.01500000 sec

======== CHANNEL f1 ========
NUC1 1H
P1 11.20 usec
PL1 0.00 dB
SFO1 400.1326008 MHz

F2 - Processing parameters
SI 16384
SF 400.1300013 MHz
WDW EM
SSB 0
LB 0.10 Hz
GB 0
PC 1.00

1D NMR plot parameters
CX 20.00 cm
CY 22.63 cm
F1P 10.500 ppm
F1 4201.37 Hz
F2P -0.500 ppm
F2 -200.06 Hz
PPMCM 0.55000 ppm/cm
HZCM 220.07150 Hz/cm

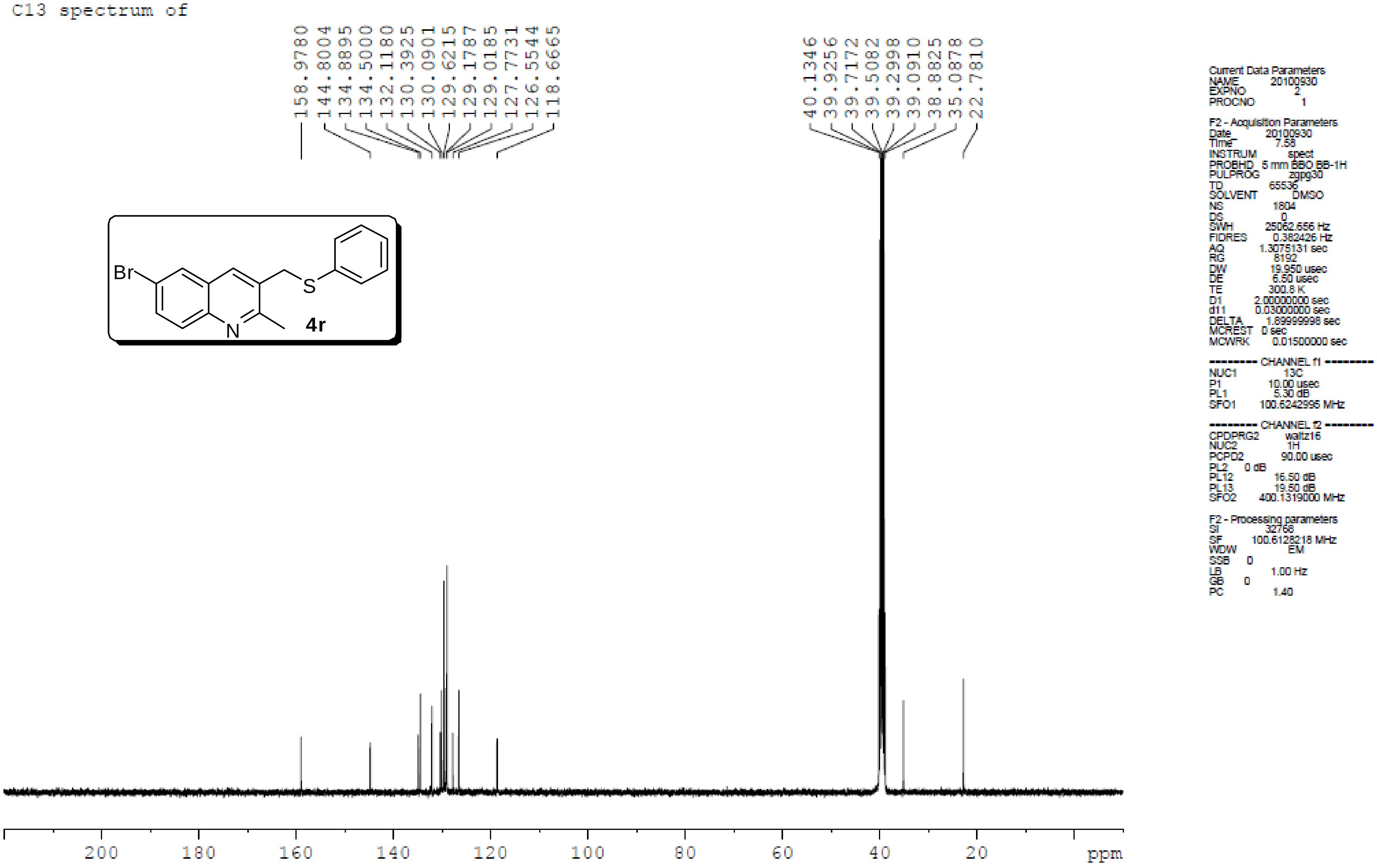
C13 spectrum of
158.9780
144.8004
134.8895
134.5000
132.1180
130.3925
130.0901
129.6215
129.1787
129.0185
127.7731
126.5544
118.6665
40.1346
39.9256
39.7172
39.5082
39.2998
39.0910
38.8825
35.0878
22.7810
Br
S
N
4r
200
180
160
140
120
100
80
60
40
20
ppm
Current Data Parameters
NAME 20100930
EXPNO 2
PROCNO 1
F2 - Acquisition Parameters
Date_ 20100930
Time 7.58
INSTRUM spect
PROBHD 5 mm BBO BB-1H
PULPROG zgpg30
TD 65536
SOLVENT DMSO
NS 1804
DS 0
SWH 25062.656 Hz
FIDRES 0.382426 Hz
AQ 1.3075131 sec
RG 8192
DW 19.950 usec
DE 6.50 usec
TE 300.8 K
D1 2.00000000 sec
d11 0.03000000 sec
DELTA 1.89999998 sec
MCREST 0 sec
MCWRK 0.01500000 sec
======== CHANNEL f1 ========
NUC1 13C
P1 10.00 usec
PL1 5.30 dB
SFO1 100.6242996 MHz
======== CHANNEL f2 ========
CPDPRG2 waltz16
NUC2 1H
PCPD2 90.00 usec
PL2 0 dB
PL12 16.50 dB
PL13 19.50 dB
SFO2 400.1319000 MHz
F2 - Processing parameters
SI 32768
SF 100.6128218 MHz
WDW EM
SSB 0
LB 1.00 Hz
GB 0
PC 1.40

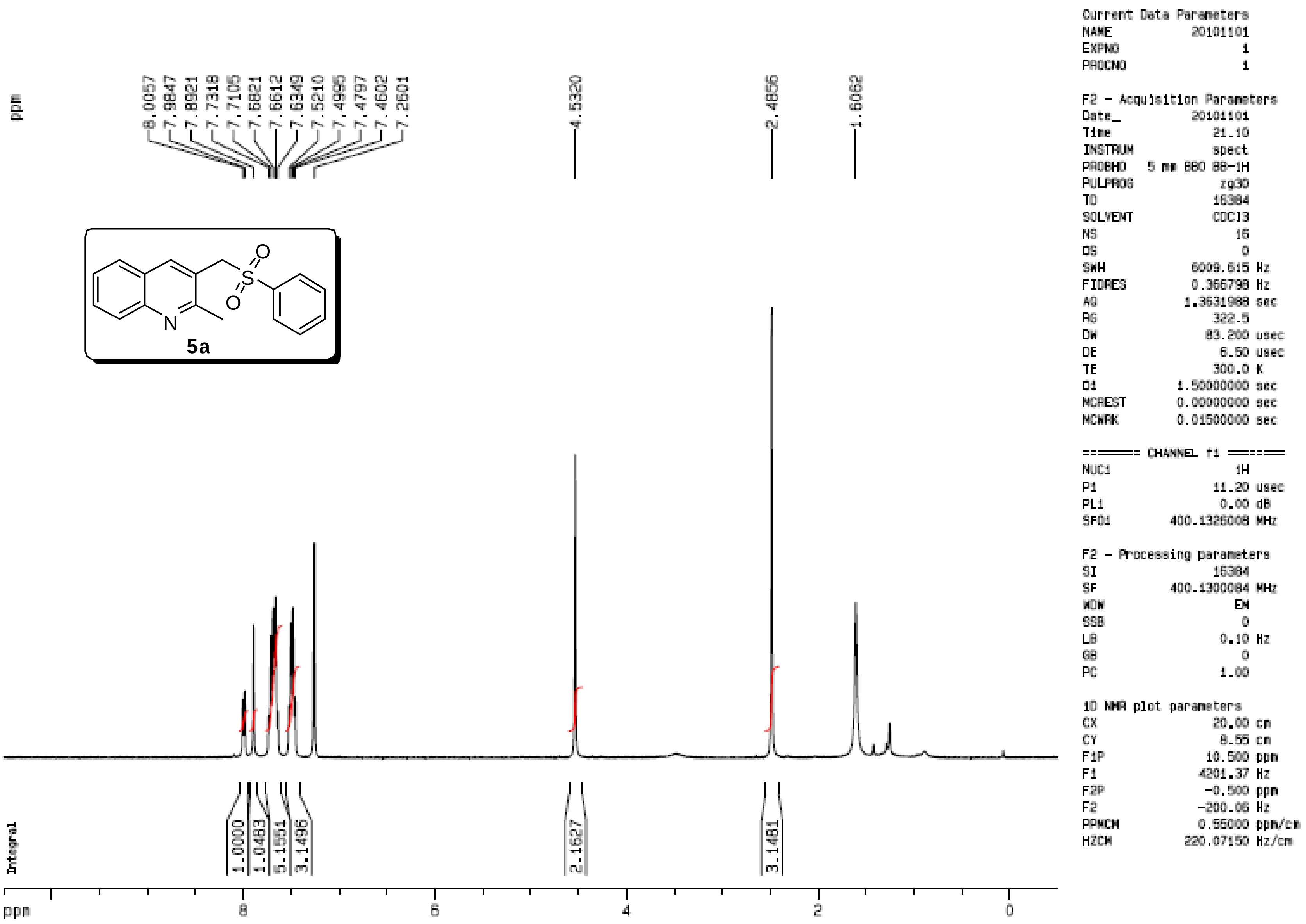
5a
8.0057
7.9847
7.8921
7.7318
7.7105
7.6821
7.6612
7.6349
7.5210
7.4995
7.4797
7.4602
7.2601
4.5320
2.4856
1.6062
1.0000
1.0483
5.1551
3.1496
2.1627
3.1481
Integral
ppm
Current Data Parameters
NAME 20101101
EXPNO 1
PROCNO 1
F2 - Acquisition Parameters
Date_ 20101101
Time 21.10
INSTRUM spect
PROBHD 5 mm BBO BB-1H
PULPROG zg30
TD 16384
SOLVENT CDCl3
NS 16
DS 0
SWH 6009.615 Hz
FIDRES 0.366798 Hz
AQ 1.3631988 sec
RG 322.5
DW 83.200 usec
DE 6.50 usec
TE 300.0 K
D1 1.50000000 sec
MCREST 0.00000000 sec
MCWRK 0.01500000 sec
======== CHANNEL f1 ========
NUC1 1H
P1 11.20 usec
PL1 0.00 dB
SFO1 400.1326008 MHz
F2 - Processing parameters
SI 16384
SF 400.1300084 MHz
WDW EM
SSB 0
LB 0.10 Hz
GB 0
PC 1.00
1D NMR plot parameters
CX 20.00 cm
CY 8.55 cm
F1P 10.500 ppm
F1 4201.37 Hz
F2P -0.500 ppm
F2 -200.06 Hz
PPMCM 0.55000 ppm/cm
HZCM 220.07150 Hz/cm

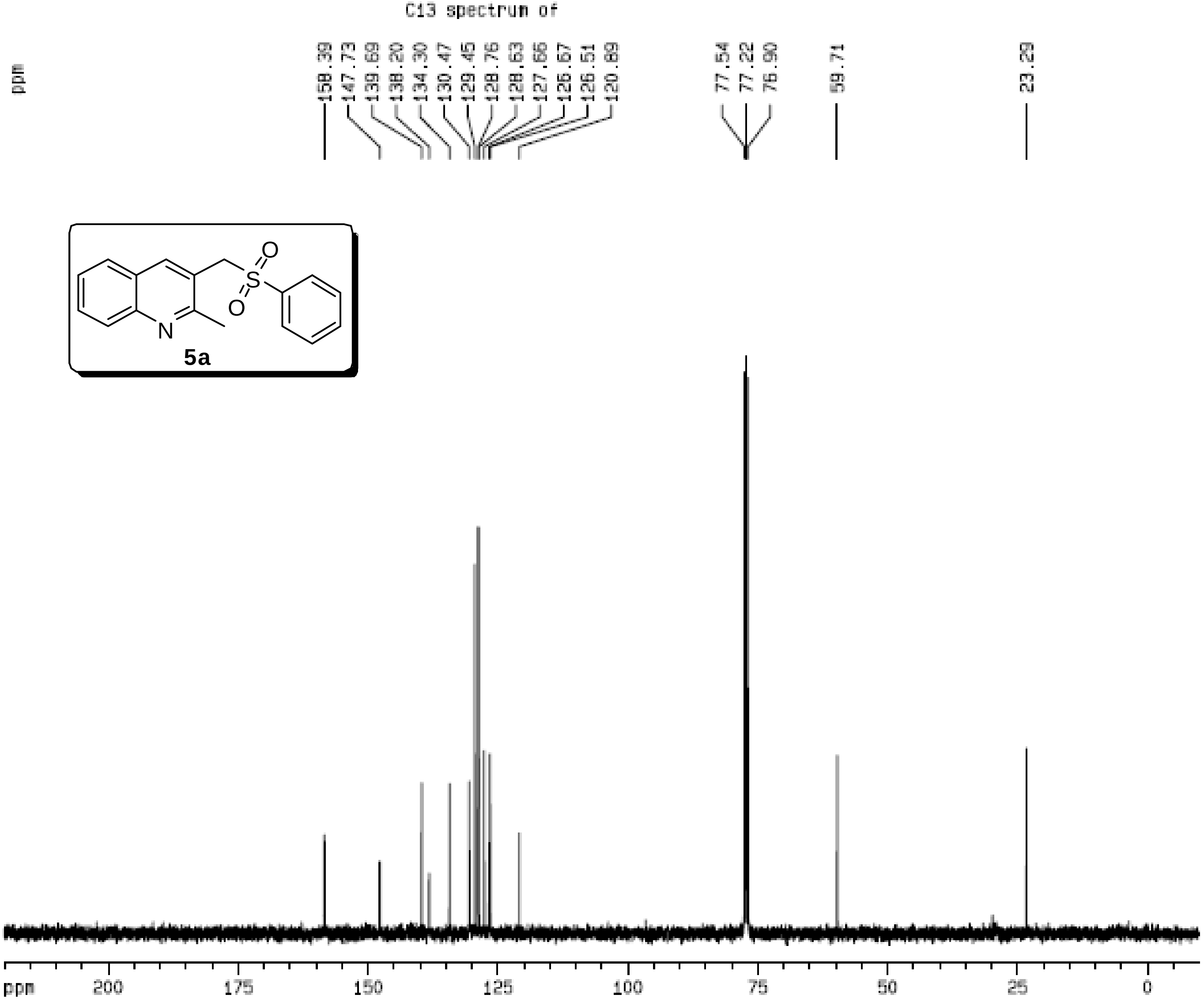

Current Data Parameters
NAME 20101028
EXPNO 2
PROCNO 1

F2 - Acquisition Parameters
Date_ 20101028
Time 15.40
INSTRUM spect
PROBHD 5 mm BBO BB-1H
PULPROG zgpg30
TD 65536
SOLVENT CDCl3
NS 153
DS 0
SWH 25062.656 Hz
FIDRES 0.382426 Hz
AQ 1.3074932 sec
RG 8192
DW 19.950 usec
DE 6.50 usec
TE 301.8 K
D1 2.00000000 sec
d11 0.03000000 sec
DELTA 1.89999998 sec
MCREST 0.00000000 sec
MCWRK 0.01500000 sec

==== CHANNEL f1 ====
NUC1 13C
P1 10.00 usec
PL1 5.30 dB
SFO1 100.6242995 MHz

==== CHANNEL f2 ====
CPDPRG2 waltz16
NUC2 1H
PCPD2 90.00 usec
PL2 0.00 dB
PL12 16.50 dB
PL13 19.50 dB
SFO2 400.1319000 MHz

F2 - Processing parameters
SI 32768
SF 100.6127538 MHz
WDW EM
SSB 0
LB 1.00 Hz
GB 0
PC 1.40

1D NMR plot parameters
CX 20.00 cm
CY 9.69 cm
F1P 220.000 ppm
F1 22134.80 Hz
F2P -10.000 ppm
F2 -1006.13 Hz
PPMCM 11.50000 ppm/cm
HZCM 1157.04663 Hz/cm

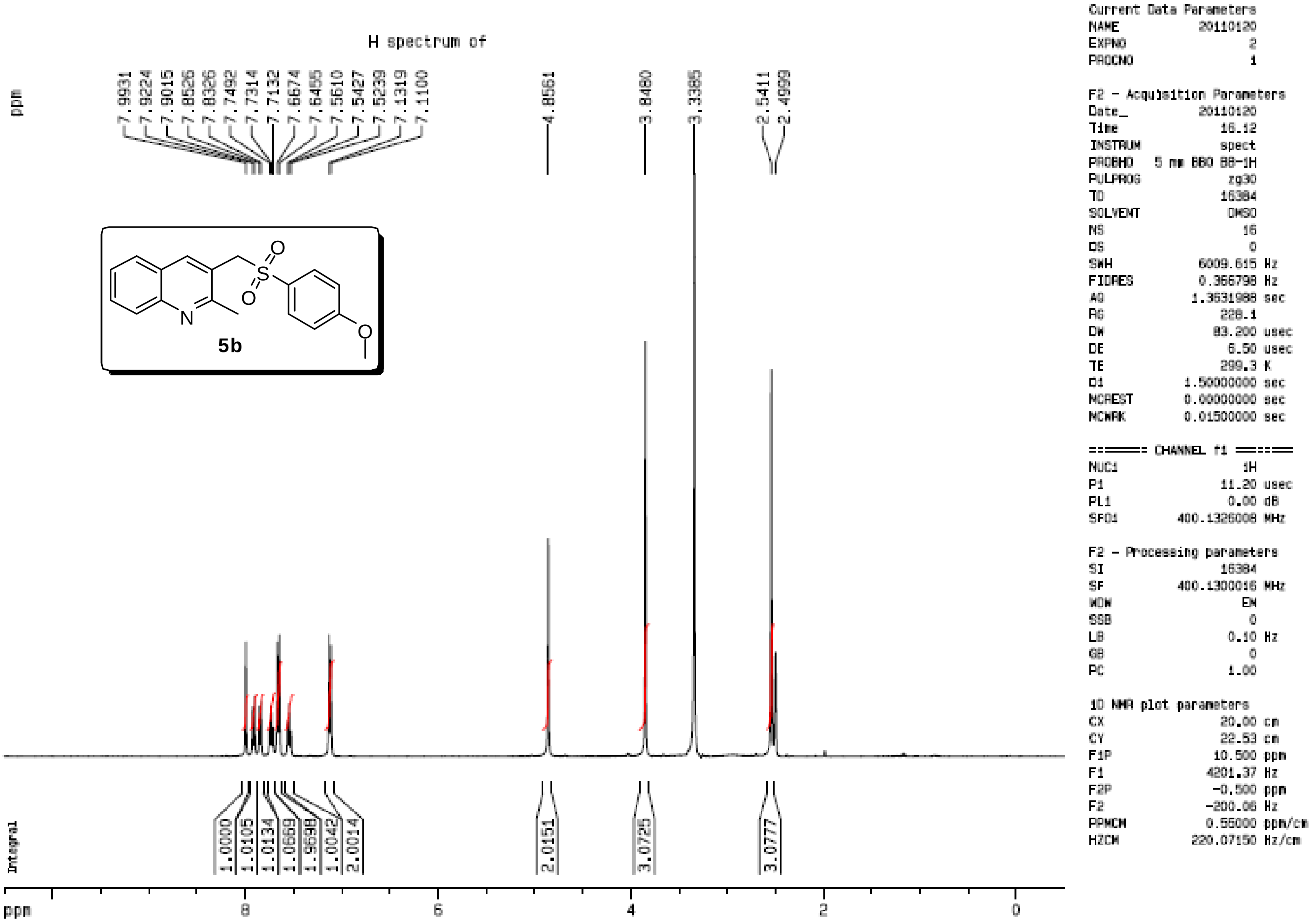
H spectrum of
5b
ppm
7.9931
7.9224
7.9015
7.8526
7.8326
7.7492
7.7314
7.7132
7.6674
7.6455
7.5610
7.5427
7.5239
7.1319
7.1100
4.8561
3.8480
3.3385
2.5411
2.4999
Integral
1.0000
1.0105
1.0134
1.0669
1.9698
1.0042
2.0014
2.0151
3.0725
3.0777
8
6
4
2
0
Current Data Parameters
NAME 20110120
EXPNO 2
PROCNO 1
F2 - Acquisition Parameters
Date_ 20110120
Time 16.12
INSTRUM spect
PROBHD 5 mm BBO BB-1H
PULPROG zg30
TD 16384
SOLVENT DMSO
NS 16
DS 0
SWH 6009.615 Hz
FIDRES 0.366798 Hz
AQ 1.3631988 sec
RG 228.1
DW 83.200 usec
DE 6.50 usec
TE 299.3 K
D1 1.50000000 sec
MCREST 0.00000000 sec
MCWRK 0.01500000 sec
======= CHANNEL f1 =======
NUC1 1H
P1 11.20 usec
PL1 0.00 dB
SFO1 400.1326008 MHz
F2 - Processing parameters
SI 16384
SF 400.1300016 MHz
WDW EM
SSB 0
LB 0.10 Hz
GB 0
PC 1.00
1D NMR plot parameters
CX 20.00 cm
CY 22.53 cm
F1P 10.500 ppm
F1 4201.37 Hz
F2P -0.500 ppm
F2 -200.06 Hz
PPMCM 0.55000 ppm/cm
HZCM 220.07150 Hz/cm

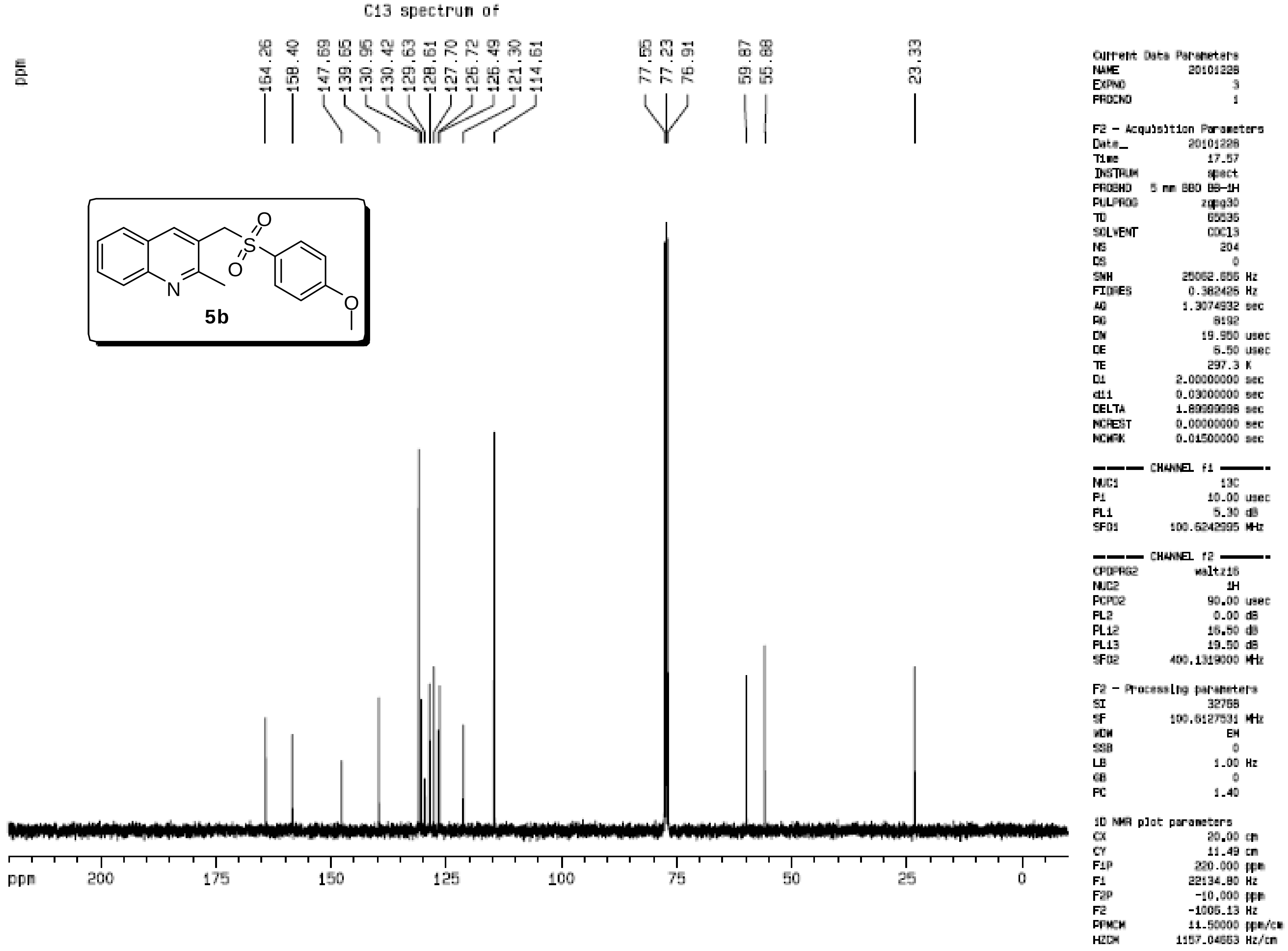
C13 spectrum of
ppm
164.26
158.40
147.69
139.65
130.95
130.42
129.63
128.61
127.70
126.72
126.49
121.30
114.61
77.55
77.23
76.91
59.87
55.88
23.33
N
S
O
O
O
5b
ppm
200
175
150
125
100
75
50
25
0
Current Data Parameters
NAME 20101228
EXPNO 3
PROCNO 1
F2 - Acquisition Parameters
Date_ 20101228
Time 17.57
INSTRUM spect
PROBHD 5 mm BBO BB-1H
PULPROG zgpg30
TD 65536
SOLVENT CDCl3
NS 204
DS 0
SWH 25062.656 Hz
FIDRES 0.382426 Hz
AQ 1.3074932 sec
RG 8192
DW 19.950 usec
DE 6.50 usec
TE 297.3 K
D1 2.00000000 sec
d11 0.03000000 sec
DELTA 1.89999998 sec
MCREST 0.00000000 sec
MCWRK 0.01500000 sec
CHANNEL f1
NUC1 13C
P1 10.00 usec
PL1 5.30 dB
SFO1 100.6242995 MHz
CHANNEL f2
CPDPRG2 waltz16
NUC2 1H
PCPD2 90.00 usec
PL2 0.00 dB
PL12 16.50 dB
PL13 19.50 dB
SFO2 400.1319000 MHz
F2 - Processing parameters
SI 32768
SF 100.6127531 MHz
WDW EM
SSB 0
LB 1.00 Hz
GB 0
PC 1.40
1D NMR plot parameters
CX 20.00 cm
CY 11.49 cm
F1P 220.000 ppm
F1 22134.80 Hz
F2P -10.000 ppm
F2 -1006.13 Hz
PPMCM 11.50000 ppm/cm
HZCM 1157.04663 Hz/cm

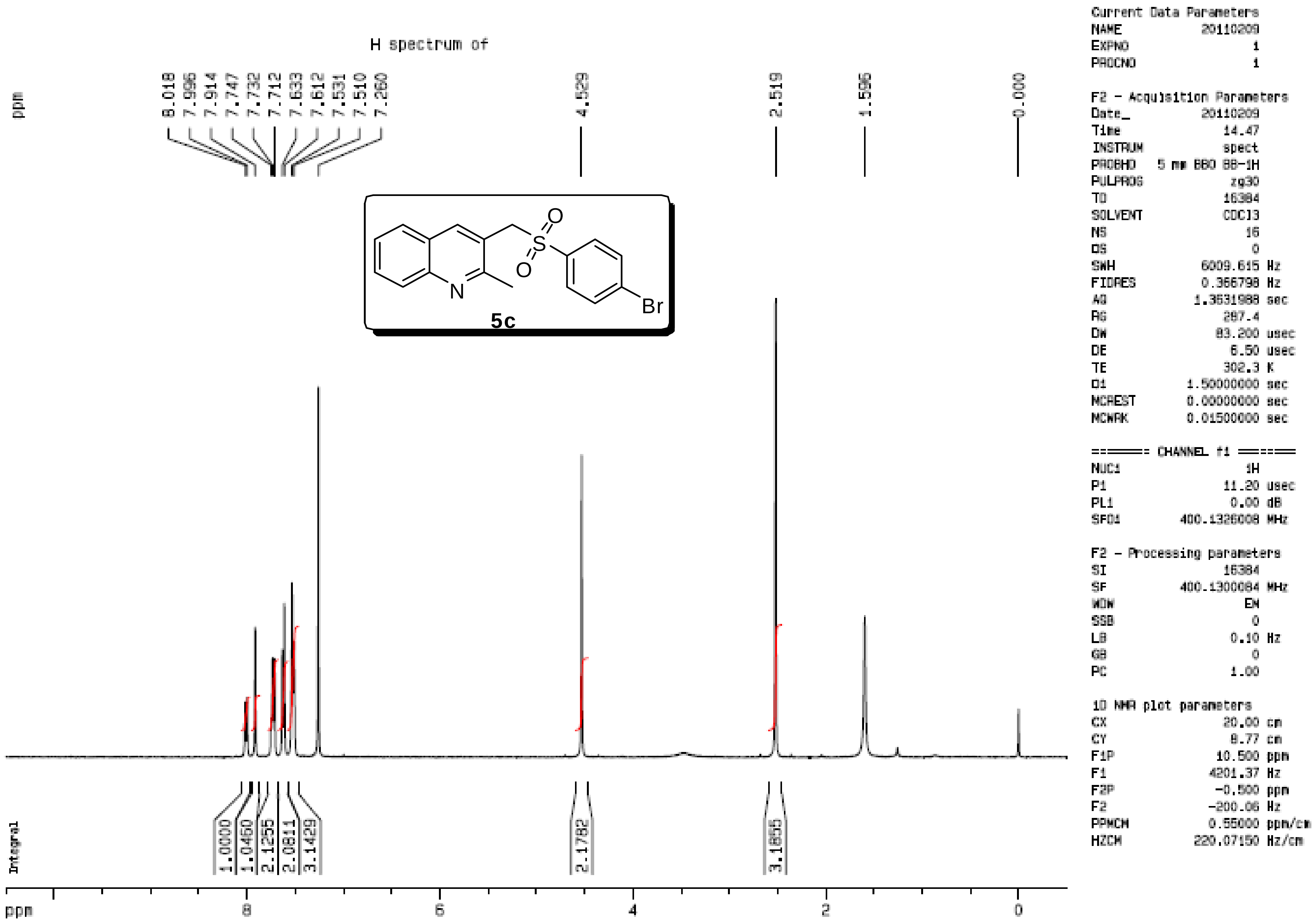
H spectrum of
ppm
8.018 7.996 7.914 7.747 7.732 7.712 7.633 7.612 7.531 7.510 7.260
4.529
2.519
1.596
0.000
N
O
S
O
Br
5c
Integral
1.0000 1.0450 2.1255 2.0811 3.1429
2.1782
3.1865
ppm
8
6
4
2
0
Current Data Parameters
NAME 20110209
EXPNO 1
PROCNO 1
F2 - Acquisition Parameters
Date_ 20110209
Time 14.47
INSTRUM spect
PROBHD 5 mm BBO BB-1H
PULPROG zg30
TD 16384
SOLVENT CDCl3
NS 16
DS 0
SWH 6009.615 Hz
FIDRES 0.366798 Hz
AQ 1.3631988 sec
RG 287.4
DW 83.200 usec
DE 6.50 usec
TE 302.3 K
D1 1.50000000 sec
MCREST 0.00000000 sec
MCWRK 0.01500000 sec
======== CHANNEL f1 ========
NUC1 1H
P1 11.20 usec
PL1 0.00 dB
SFO1 400.1326008 MHz
F2 - Processing parameters
SI 16384
SF 400.1300084 MHz
WDW EM
SSB 0
LB 0.10 Hz
GB 0
PC 1.00
1D NMR plot parameters
CX 20.00 cm
CY 8.77 cm
F1P 10.500 ppm
F1 4201.37 Hz
F2P -0.500 ppm
F2 -200.06 Hz
PPMCM 0.55000 ppm/cm
HZCM 220.07150 Hz/cm

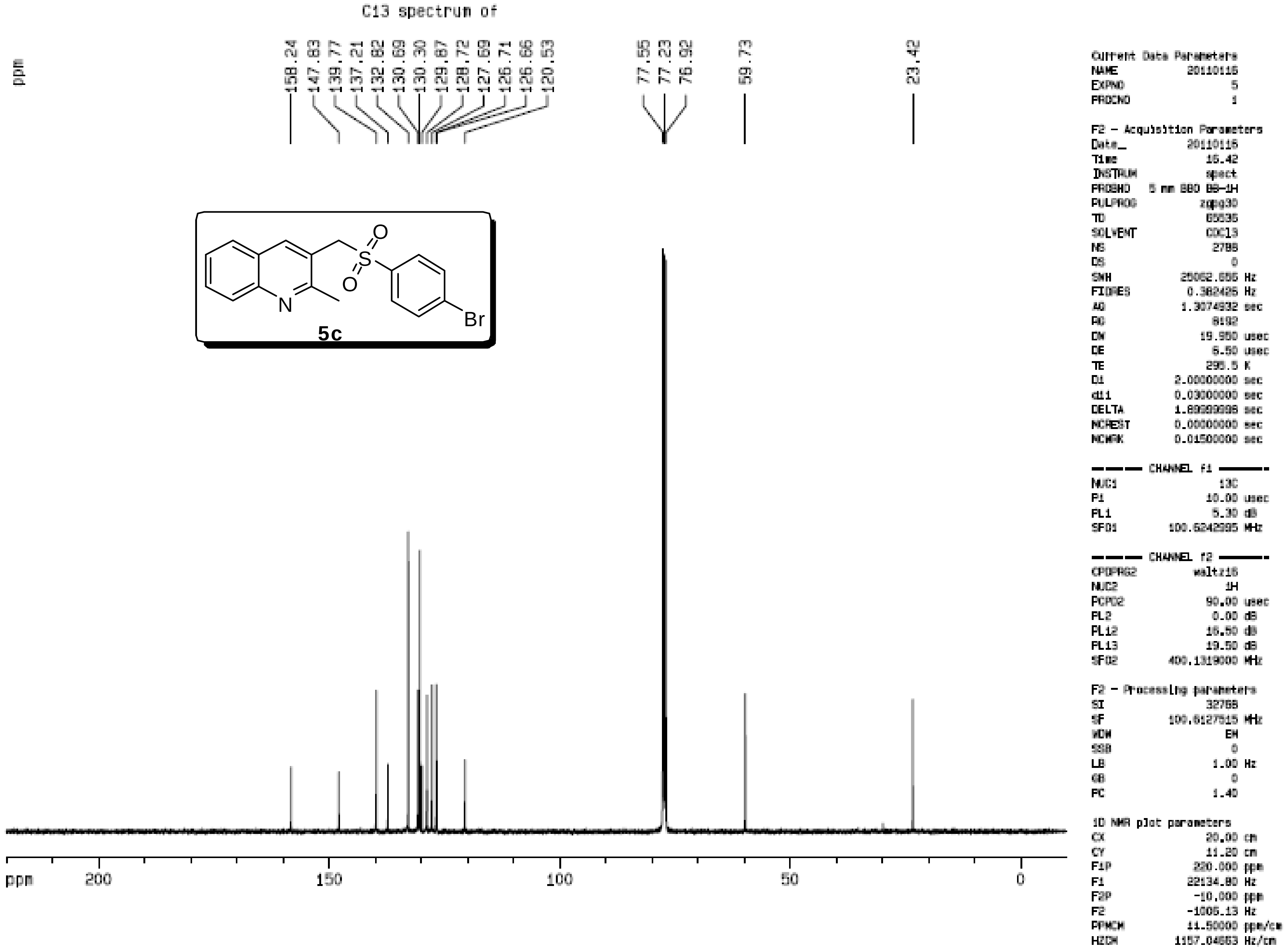
C13 spectrum of
ppm
158.24
147.83
139.77
137.21
132.82
130.69
130.30
129.87
128.72
127.69
126.71
126.66
120.53
77.55
77.23
76.92
59.73
23.42
O
S
O
N
Br
5c
ppm 200 150 100 50 0
Current Data Parameters
NAME 20110116
EXPNO 5
PROCNO 1
F2 - Acquisition Parameters
Date_ 20110116
Time 16.42
INSTRUM spect
PROBHD 5 mm BBO BB-1H
PULPROG zgpg30
TD 65536
SOLVENT CDCl3
NS 2788
DS 0
SWH 25062.656 Hz
FIDRES 0.382426 Hz
AQ 1.3074932 sec
RG 8192
DW 19.950 usec
DE 6.50 usec
TE 295.5 K
D1 2.00000000 sec
d11 0.03000000 sec
DELTA 1.89999998 sec
MCREST 0.00000000 sec
MCWRK 0.01500000 sec
CHANNEL f1
NUC1 13C
P1 10.00 usec
PL1 5.30 dB
SFO1 100.6242995 MHz
CHANNEL f2
CPDPRG2 waltz16
NUC2 1H
PCPD2 90.00 usec
PL2 0.00 dB
PL12 16.50 dB
PL13 19.50 dB
SFO2 400.1319000 MHz
F2 - Processing parameters
SI 32768
SF 100.6127515 MHz
WDW EM
SSB 0
LB 1.00 Hz
GB 0
PC 1.40
1D NMR plot parameters
CX 20.00 cm
CY 11.20 cm
F1P 220.000 ppm
F1 22134.80 Hz
F2P -10.000 ppm
F2 -1006.13 Hz
PPMCM 11.50000 ppm/cm
HZCM 1157.04663 Hz/cm

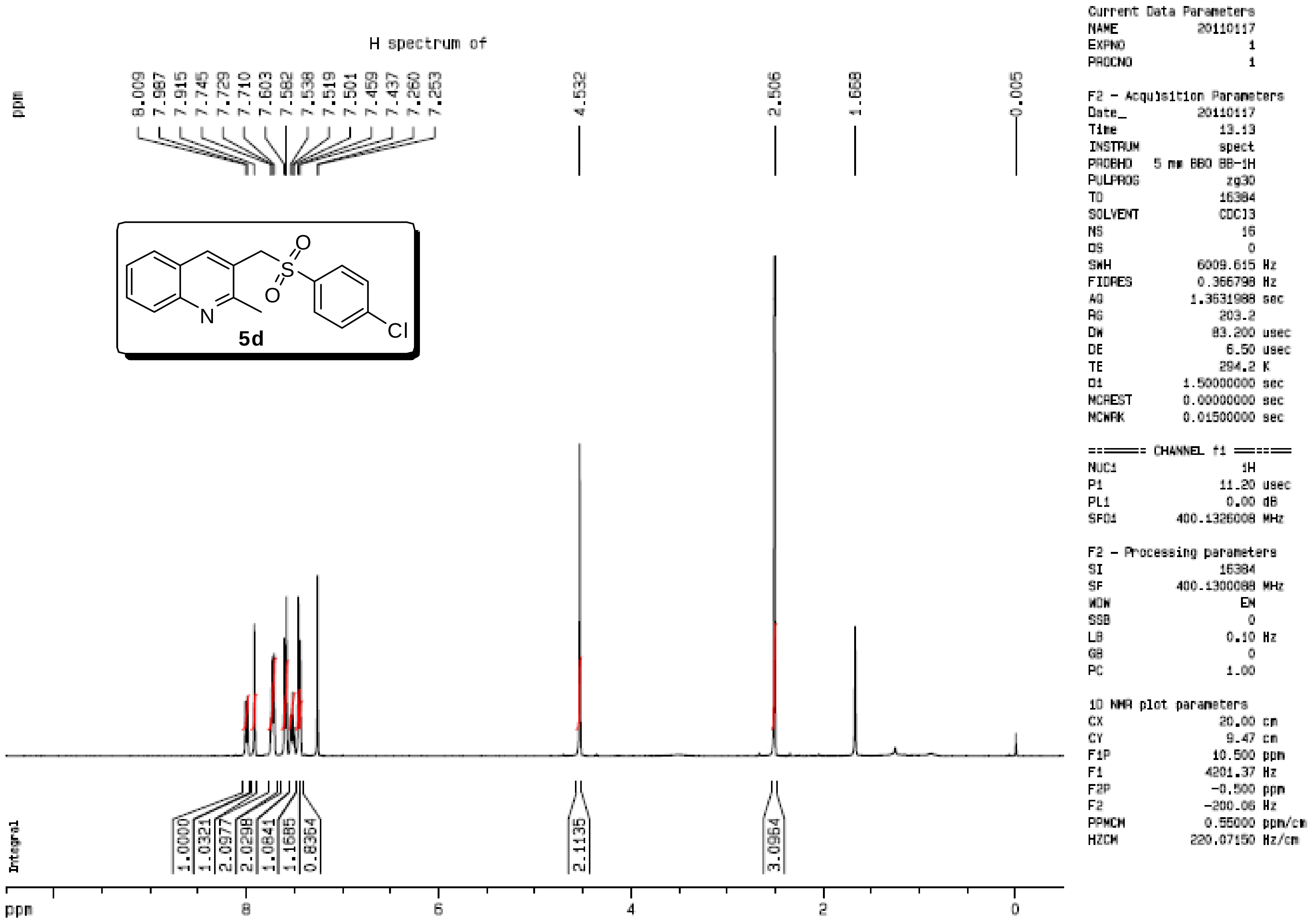
H spectrum of
8.009
7.987
7.915
7.745
7.729
7.710
7.603
7.582
7.538
7.519
7.501
7.459
7.437
7.260
7.253
4.532
2.506
1.668
0.005
5d
Integral
1.0000
1.0321
2.0977
2.0298
1.0841
1.1685
0.8364
2.1135
3.0964
ppm
8
6
4
2
0
Current Data Parameters
NAME 20110117
EXPNO 1
PROCNO 1
F2 - Acquisition Parameters
Date_ 20110117
Time 13.13
INSTRUM spect
PROBHD 5 mm BBO BB-1H
PULPROG zg30
TD 16384
SOLVENT CDCl3
NS 16
DS 0
SWH 6009.615 Hz
FIDRES 0.366798 Hz
AQ 1.3631988 sec
RG 203.2
DW 83.200 usec
DE 6.50 usec
TE 294.2 K
D1 1.50000000 sec
MCREST 0.00000000 sec
MCWRK 0.01500000 sec
======== CHANNEL f1 ========
NUC1 1H
P1 11.20 usec
PL1 0.00 dB
SFO1 400.1326008 MHz
F2 - Processing parameters
SI 16384
SF 400.1300088 MHz
WDW EM
SSB 0
LB 0.10 Hz
GB 0
PC 1.00
1D NMR plot parameters
CX 20.00 cm
CY 9.47 cm
F1P 10.500 ppm
F1 4201.37 Hz
F2P -0.500 ppm
F2 -200.06 Hz
PPMCM 0.55000 ppm/cm
HZCM 220.07150 Hz/cm

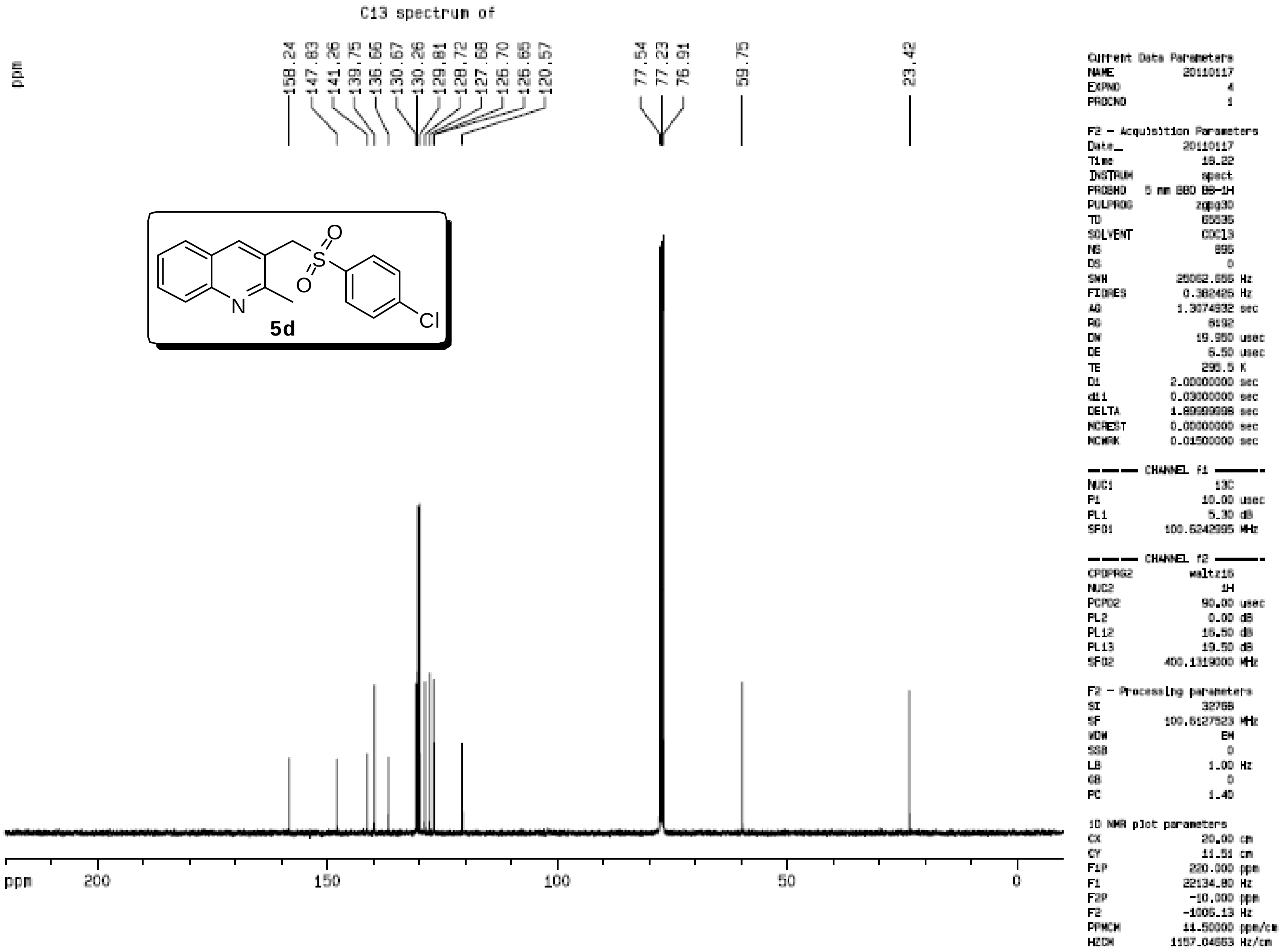
C13 spectrum of
158.24
147.83
141.26
139.75
136.66
130.67
130.26
129.61
128.72
127.68
126.70
126.65
120.57
77.54
77.23
76.91
59.75
23.42
5d
N
O
S
Cl
ppm
200
150
100
50
0
Current Data Parameters
NAME 20110117
EXPNO 4
PROCNO 1
F2 - Acquisition Parameters
INSTRUM spect
SOLVENT CDCl3
PULPROG zgpg30
TD 65536
NUC1 13C
CPDPRG2 waltz16
NUC2 1H
SF 100.6127523 MHz
SFO2 400.1319000 MHz

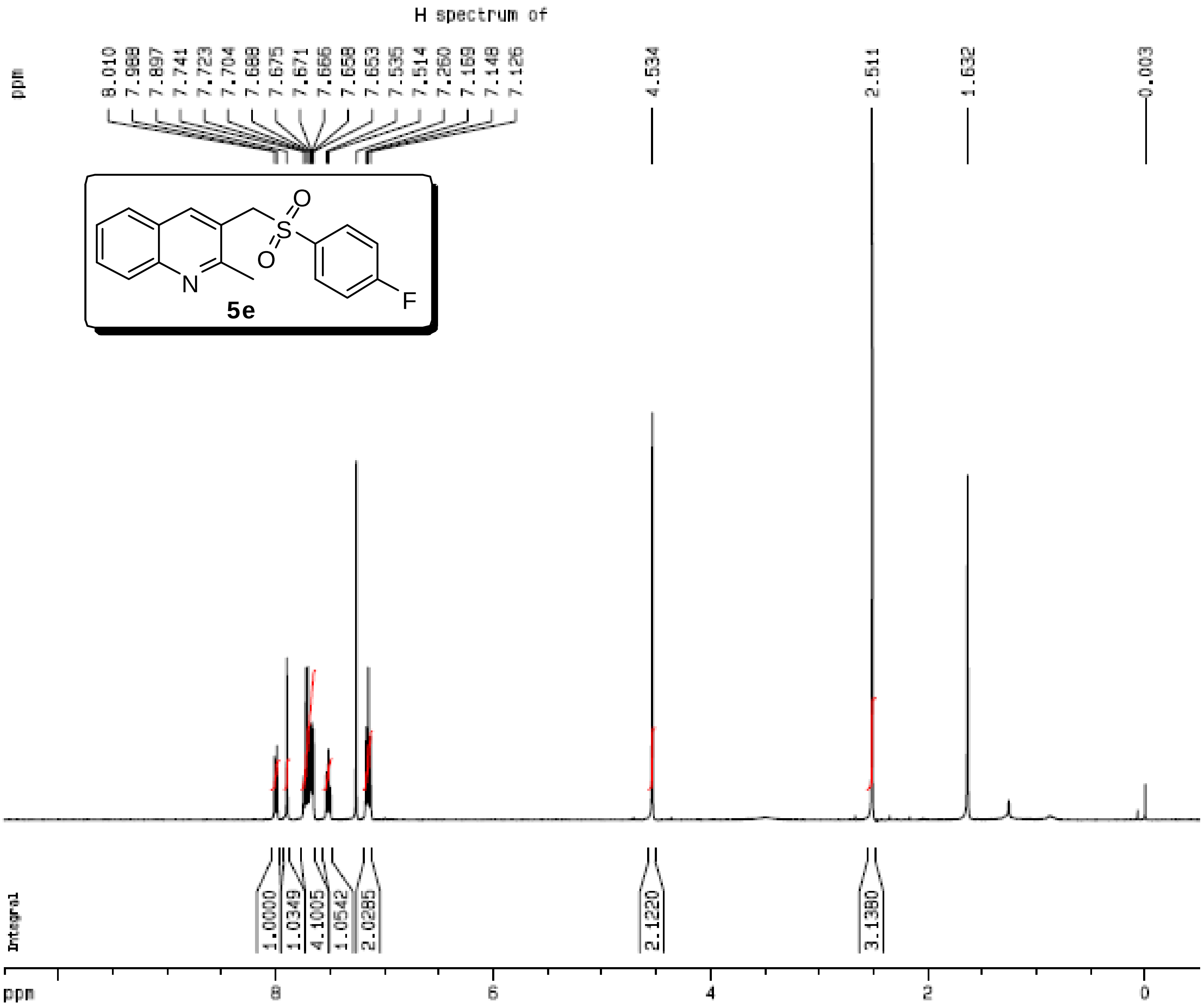

Current Data Parameters
NAME 20110120
EXPNO 1
PROCNO 1

F2 - Acquisition Parameters
Date_ 20110120
Time 14.56
INSTRUM spect
PROBHD 5 mm BBO BB-1H
PULPROG zg30
TD 16384
SOLVENT CDCl3
NS 16
DS 0
SWH 6009.615 Hz
FIDRES 0.366798 Hz
AQ 1.3631988 sec
RG 322.5
DW 83.200 usec
DE 6.50 usec
TE 299.1 K
D1 1.50000000 sec
MCREST 0.00000000 sec
MCWRK 0.01500000 sec

======== CHANNEL f1 ========
NUC1 1H
P1 11.20 usec
PL1 0.00 dB
SFO1 400.1326008 MHz

F2 - Processing parameters
SI 16384
SF 400.1300088 MHz
WDW EM
SSB 0
LB 0.10 Hz
GB 0
PC 1.00

1D NMR plot parameters
CX 20.00 cm
CY 11.42 cm
F1P 10.500 ppm
F1 4201.37 Hz
F2P -0.500 ppm
F2 -200.06 Hz
PPMCM 0.55000 ppm/cm
HZCM 220.07150 Hz/cm

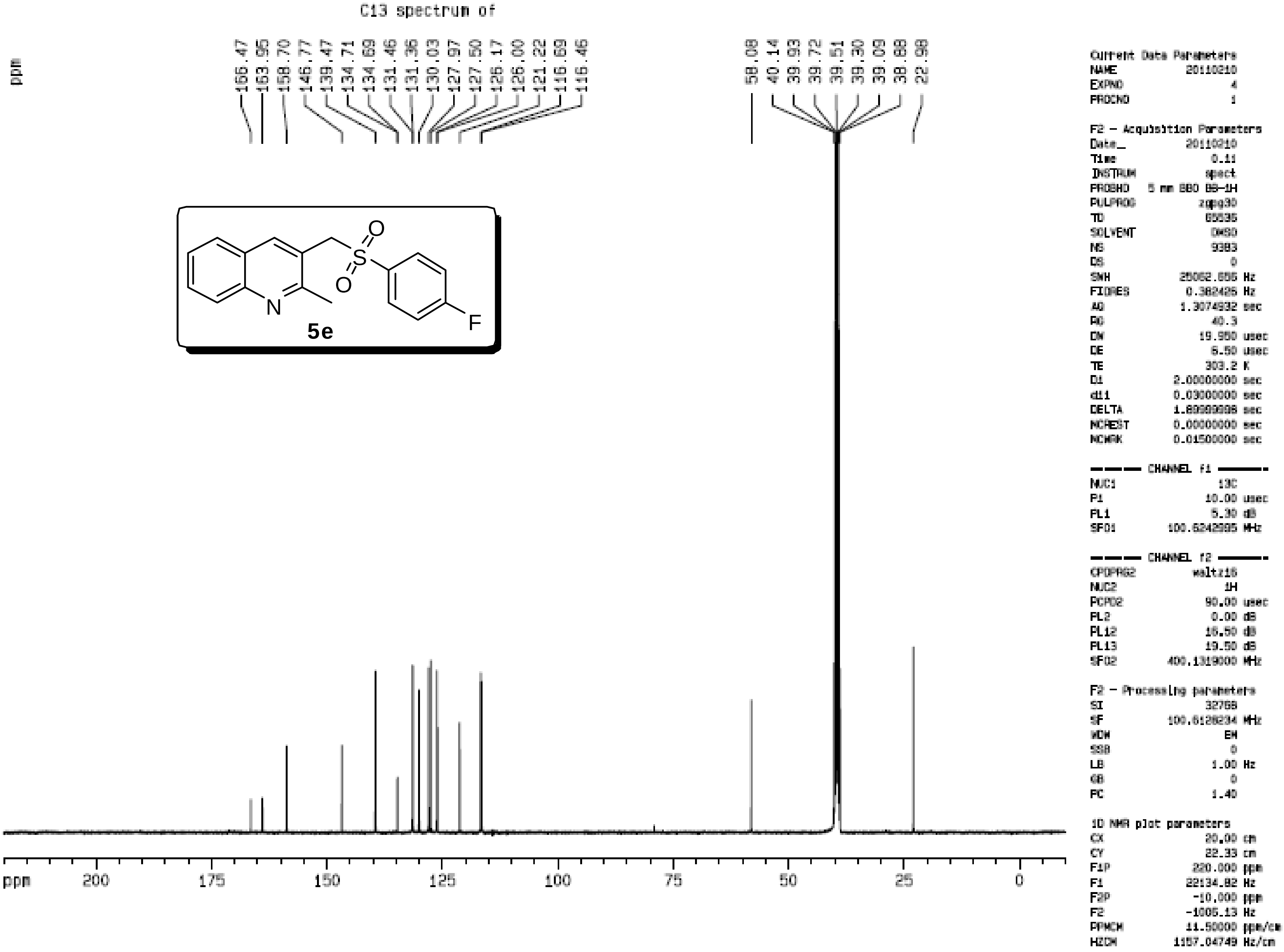
C13 spectrum of
166.47
163.95
158.70
146.77
139.47
134.71
134.69
131.46
131.36
130.03
127.97
127.50
126.17
126.00
121.22
116.69
116.46
58.08
40.14
39.93
39.72
39.51
39.30
39.09
38.88
22.98
5e
ppm
200
175
150
125
100
75
50
25
0

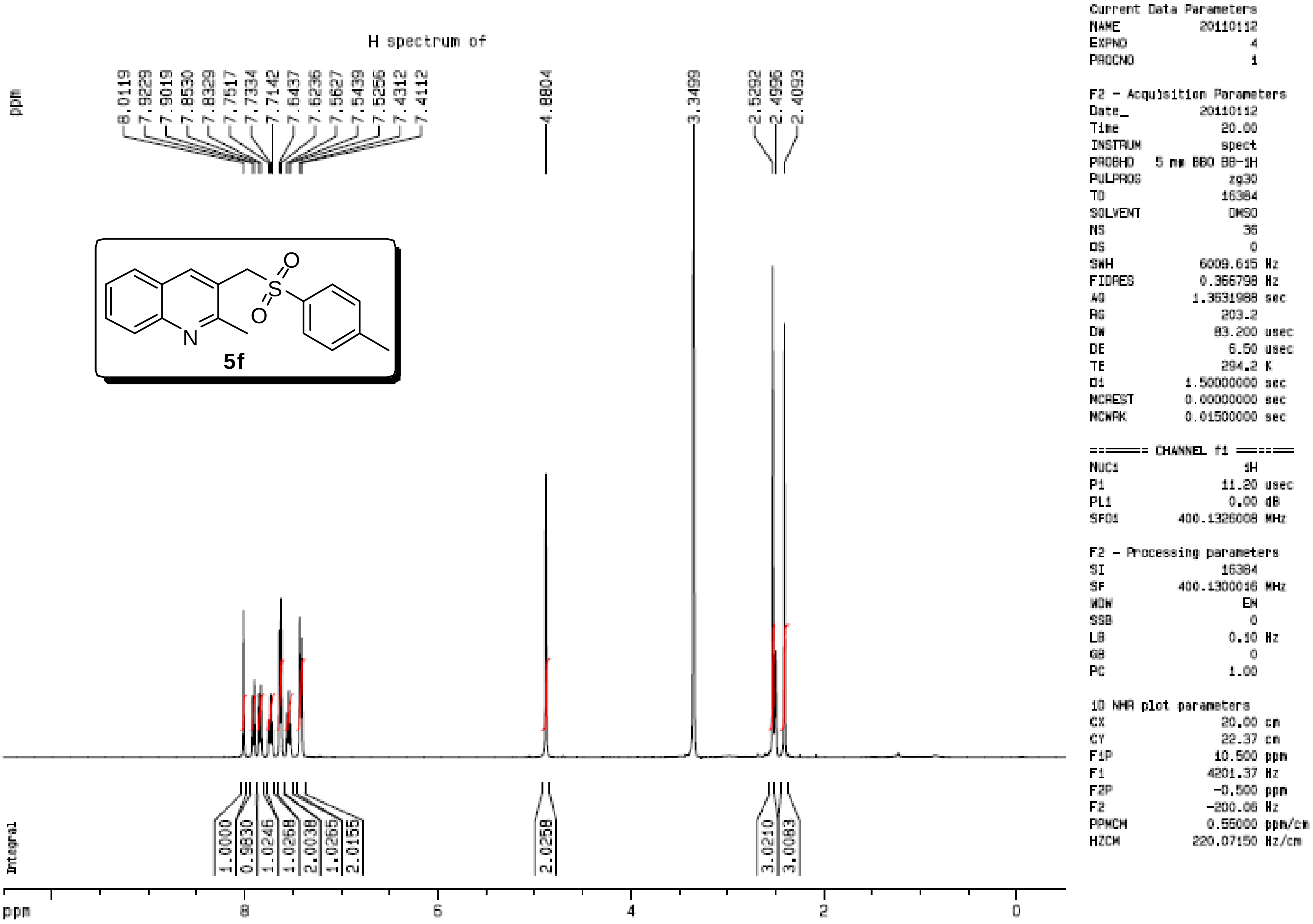
H spectrum of
ppm
8.0119
7.9229
7.9019
7.8530
7.8329
7.7517
7.7334
7.7142
7.6437
7.6236
7.5627
7.5439
7.5256
7.4312
7.4112
4.8804
3.3499
2.5292
2.4996
2.4093
5f
Integral
1.0000
0.9830
1.0246
1.0268
2.0038
1.0265
2.0155
2.0258
3.0210
3.0083
ppm 8 6 4 2 0
Current Data Parameters
NAME 20110112
EXPNO 4
PROCNO 1
F2 - Acquisition Parameters
Date_ 20110112
Time 20.00
INSTRUM spect
PROBHD 5 mm BBO BB-1H
PULPROG zg30
TD 16384
SOLVENT DMSO
NS 36
DS 0
SWH 6009.615 Hz
FIDRES 0.366798 Hz
AQ 1.3631988 sec
RG 203.2
DW 83.200 usec
DE 6.50 usec
TE 294.2 K
D1 1.50000000 sec
MCREST 0.00000000 sec
MCWRK 0.01500000 sec
======= CHANNEL f1 =======
NUC1 1H
P1 11.20 usec
PL1 0.00 dB
SFO1 400.1326008 MHz
F2 - Processing parameters
SI 16384
SF 400.1300016 MHz
WDW EM
SSB 0
LB 0.10 Hz
GB 0
PC 1.00
1D NMR plot parameters
CX 20.00 cm
CY 22.37 cm
F1P 10.500 ppm
F1 4201.37 Hz
F2P -0.500 ppm
F2 -200.06 Hz
PPMCM 0.55000 ppm/cm
HZCM 220.07150 Hz/cm

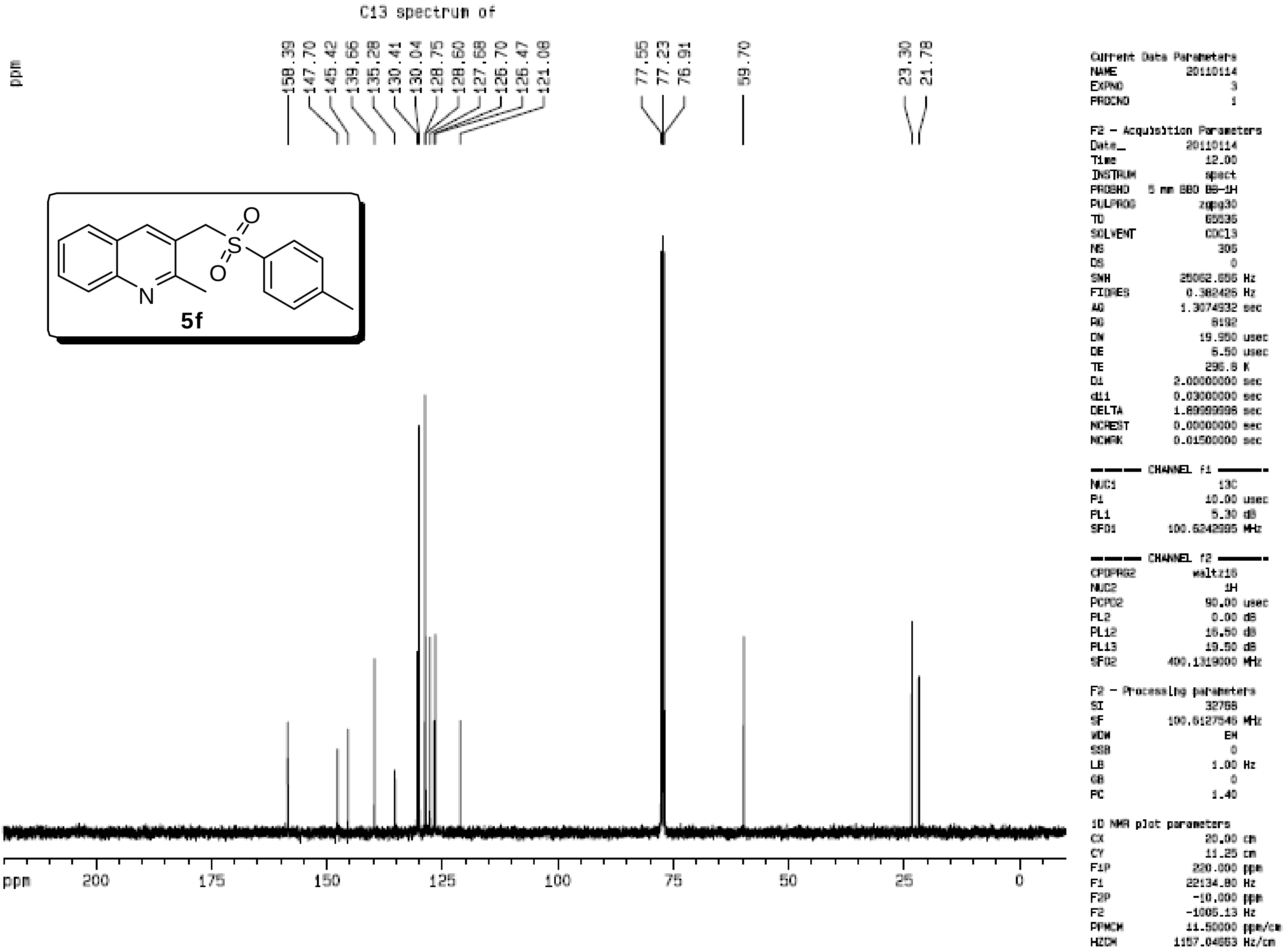
C13 spectrum of
ppm
158.39
147.70
145.42
139.66
135.28
130.41
130.04
128.75
128.60
127.68
126.70
126.47
121.08
77.55
77.23
76.91
59.70
23.30
21.78
N
O
S
O
5f
ppm 200 175 150 125 100 75 50 25 0
Current Data Parameters
NAME 20110114
EXPNO 3
PROCNO 1
F2 - Acquisition Parameters
Date_ 20110114
Time 12.00
INSTRUM spect
PROBHD 5 mm BBO BB-1H
PULPROG zgpg30
TD 65536
SOLVENT CDCl3
NS 305
DS 0
SWH 25062.656 Hz
FIDRES 0.382426 Hz
AQ 1.3074932 sec
RG 8192
DW 19.950 usec
DE 6.50 usec
TE 296.8 K
D1 2.00000000 sec
d11 0.03000000 sec
DELTA 1.89999998 sec
MCREST 0.00000000 sec
MCWRK 0.01500000 sec
CHANNEL f1
NUC1 13C
P1 10.00 usec
PL1 5.30 dB
SFO1 100.6242695 MHz
CHANNEL f2
CPDPRG2 waltz16
NUC2 1H
PCPD2 90.00 usec
PL2 0.00 dB
PL12 16.50 dB
PL13 19.50 dB
SFO2 400.1319000 MHz
F2 - Processing parameters
SI 32768
SF 100.6127546 MHz
WDW EM
SSB 0
LB 1.00 Hz
GB 0
PC 1.40
1D NMR plot parameters
CX 20.00 cm
CY 11.25 cm
F1P 220.000 ppm
F1 22134.80 Hz
F2P -10.000 ppm
F2 -1006.13 Hz
PPMCM 11.50000 ppm/cm
HZCM 1157.04663 Hz/cm

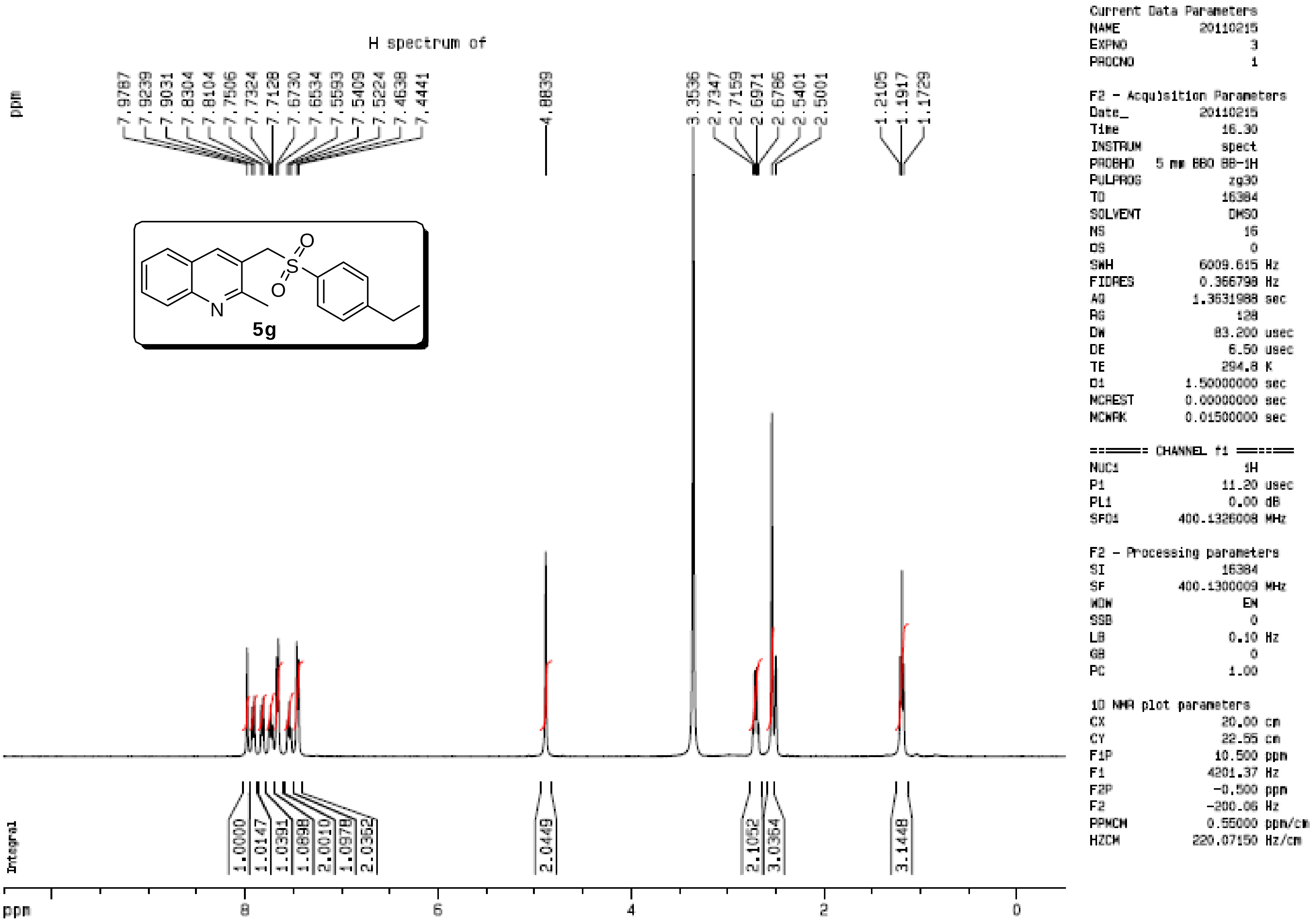
H spectrum of
ppm
7.9787 7.9239 7.9031 7.8304 7.8104 7.7506 7.7324 7.7128 7.6730 7.6534 7.5593 7.5409 7.5224 7.4638 7.4441
4.8839
3.3536 2.7347 2.7159 2.6971 2.6786 2.5401 2.5001
1.2105 1.1917 1.1729
N
O
S
O
5g
Integral
1.0000 1.0147 1.0391 1.0898 2.0010 1.0979 2.0362
2.0449
2.1052 3.0364
3.1448
ppm
8
6
4
2
0
Current Data Parameters
NAME 20110215
EXPNO 3
PROCNO 1
F2 - Acquisition Parameters
Date_ 20110215
Time 16.30
INSTRUM spect
PROBHD 5 mm BBO BB-1H
PULPROG zg30
TD 16384
SOLVENT DMSO
NS 16
DS 0
SWH 6009.615 Hz
FIDRES 0.366798 Hz
AQ 1.3631988 sec
RG 128
DW 83.200 usec
DE 6.50 usec
TE 294.8 K
D1 1.50000000 sec
MCREST 0.00000000 sec
MCWRK 0.01500000 sec
======= CHANNEL f1 =======
NUC1 1H
P1 11.20 usec
PL1 0.00 dB
SFO1 400.1326008 MHz
F2 - Processing parameters
SI 16384
SF 400.1300009 MHz
WDW EM
SSB 0
LB 0.10 Hz
GB 0
PC 1.00
1D NMR plot parameters
CX 20.00 cm
CY 22.55 cm
F1P 10.500 ppm
F1 4201.37 Hz
F2P -0.500 ppm
F2 -200.06 Hz
PPMCM 0.55000 ppm/cm
HZCM 220.07150 Hz/cm

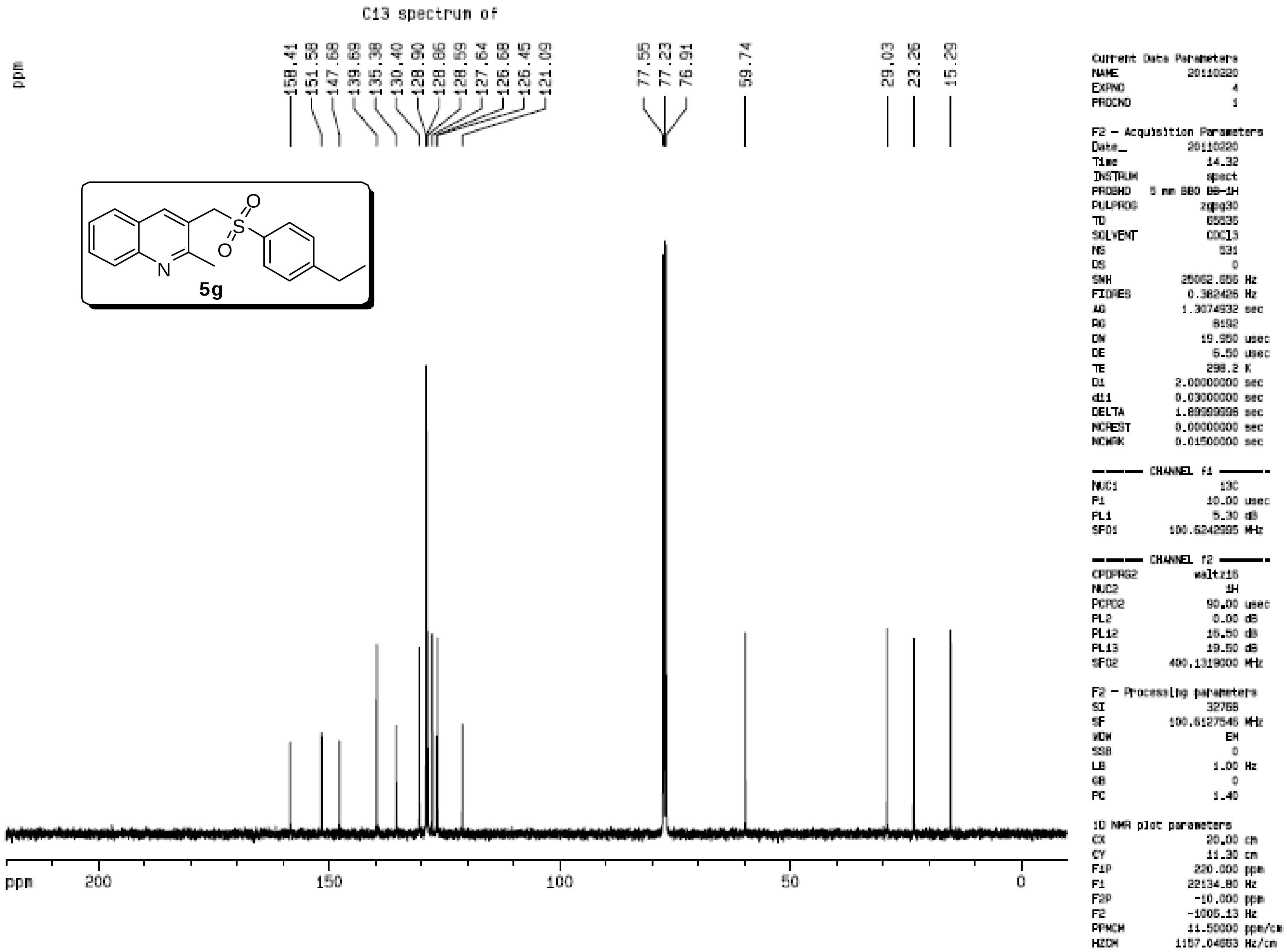
C13 spectrum of
158.41
151.58
147.68
139.69
135.38
130.40
128.90
128.86
128.59
127.64
126.68
126.45
121.09
77.55
77.23
76.91
59.74
29.03
23.26
15.29
O
S
O
N
5g
ppm
200
150
100
50
0
Current Data Parameters
NAME 20110220
EXPNO 4
PROCNO 1
F2 - Acquisition Parameters
Date_ 20110220
Time 14.32
INSTRUM spect
PROBHD 5 mm BBO BB-1H
PULPROG zgpg30
TD 65536
SOLVENT CDCl3
NS 531
DS 0
SWH 25062.656 Hz
FIDRES 0.382426 Hz
AQ 1.3074932 sec
RG 8192
DW 19.950 usec
DE 6.50 usec
TE 298.2 K
D1 2.00000000 sec
d11 0.03000000 sec
DELTA 1.89999998 sec
MCREST 0.00000000 sec
MCWRK 0.01500000 sec
CHANNEL f1
NUC1 13C
P1 10.00 usec
PL1 5.30 dB
SFO1 100.6242995 MHz
CHANNEL f2
CPDPRG2 waltz16
NUC2 1H
PCPD2 90.00 usec
PL2 0.00 dB
PL12 16.50 dB
PL13 19.50 dB
SFO2 400.1319000 MHz
F2 - Processing parameters
SI 32768
SF 100.6127546 MHz
WDW EM
SSB 0
LB 1.00 Hz
GB 0
PC 1.40
1D NMR plot parameters
CX 20.00 cm
CY 11.30 cm
F1P 220.000 ppm
F1 22134.80 Hz
F2P -10.000 ppm
F2 -1006.13 Hz
PPMCM 11.50000 ppm/cm
HZCM 1157.04663 Hz/cm

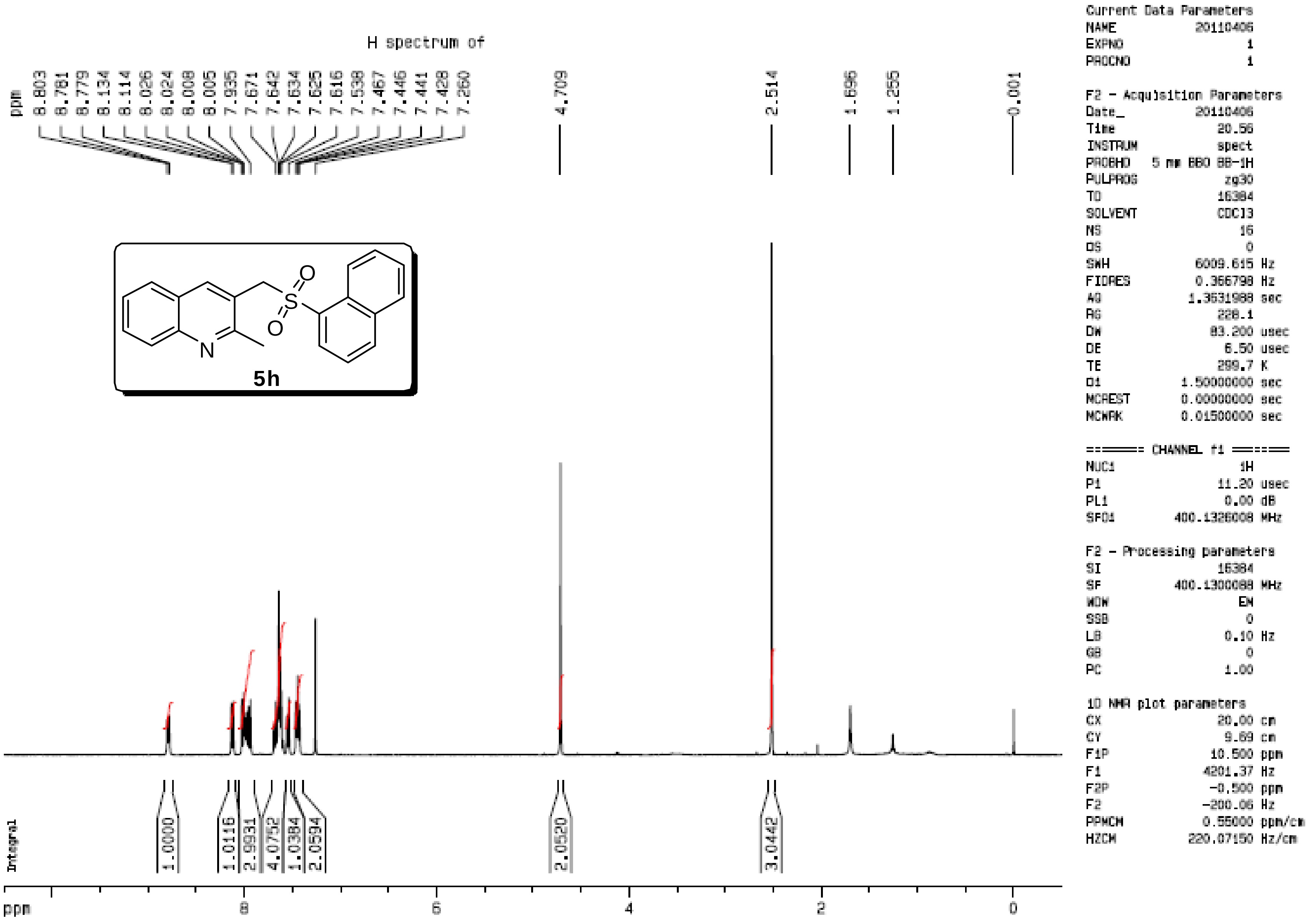
H spectrum of
ppm
8.803
8.781
8.779
8.134
8.114
8.026
8.024
8.008
8.005
7.935
7.671
7.642
7.634
7.625
7.616
7.538
7.467
7.446
7.441
7.428
7.260
4.709
2.514
1.696
1.255
-0.001
5h
Integral
1.0000
1.0116
2.9931
4.0752
1.0384
2.0594
2.0520
3.0442
ppm
8
6
4
2
0
Current Data Parameters
NAME 20110406
EXPNO 1
PROCNO 1
F2 - Acquisition Parameters
Date_ 20110406
Time 20.56
INSTRUM spect
PROBHD 5 mm BBO BB-1H
PULPROG zg30
TD 16384
SOLVENT CDCl3
NS 16
DS 0
SWH 6009.615 Hz
FIDRES 0.366798 Hz
AQ 1.3631988 sec
RG 228.1
DW 83.200 usec
DE 6.50 usec
TE 299.7 K
D1 1.50000000 sec
MCREST 0.00000000 sec
MCWRK 0.01500000 sec
======== CHANNEL f1 ========
NUC1 1H
P1 11.20 usec
PL1 0.00 dB
SFO1 400.1326008 MHz
F2 - Processing parameters
SI 16384
SF 400.1300088 MHz
WDW EM
SSB 0
LB 0.10 Hz
GB 0
PC 1.00
1D NMR plot parameters
CX 20.00 cm
CY 9.69 cm
F1P 10.500 ppm
F1 4201.37 Hz
F2P -0.500 ppm
F2 -200.06 Hz
PPMCM 0.55000 ppm/cm
HZCM 220.07150 Hz/cm

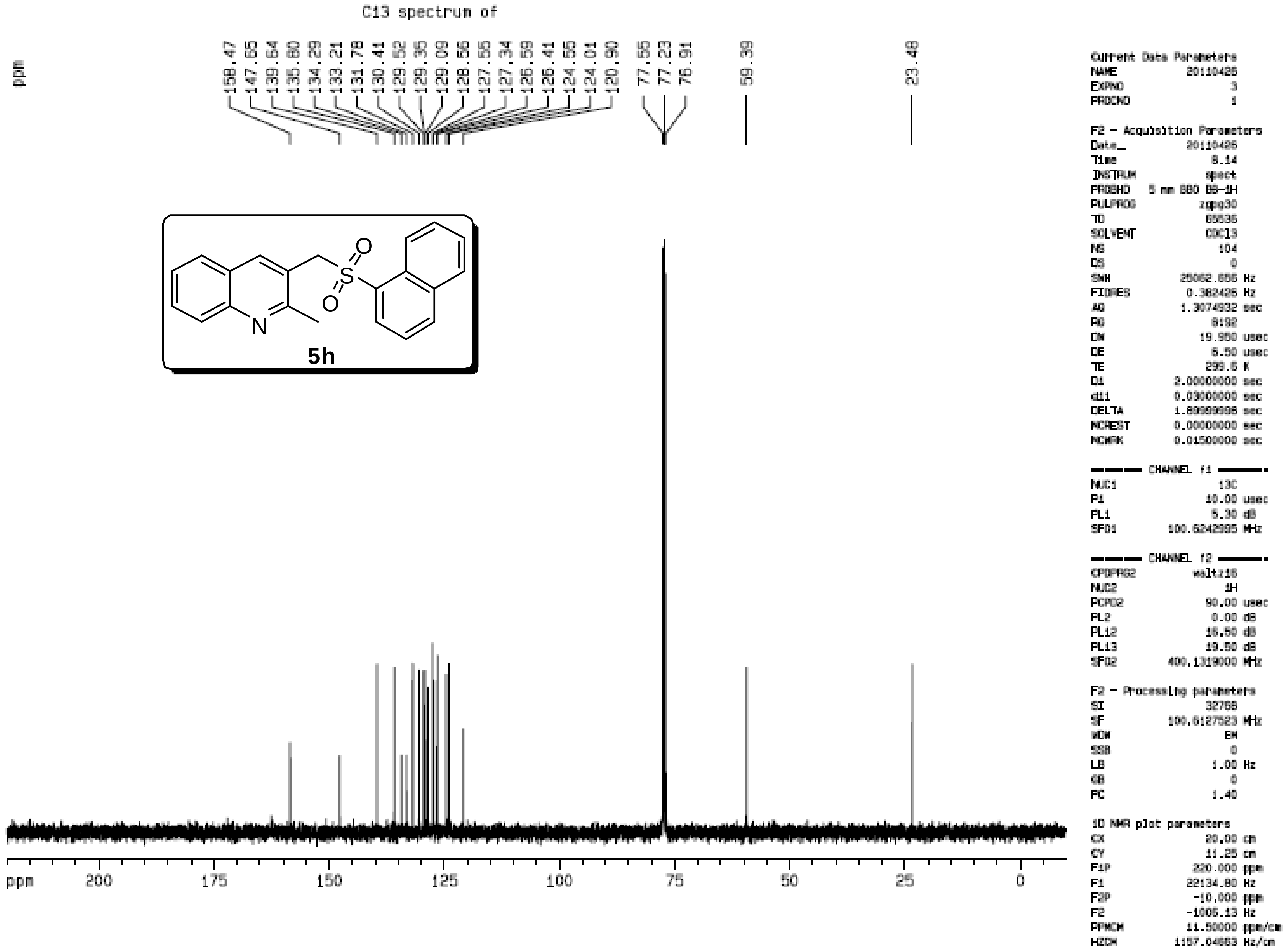
C13 spectrum of
ppm
158.47
147.65
139.64
135.80
134.29
133.21
131.78
130.41
129.52
129.35
129.09
128.56
127.55
127.34
126.59
126.41
124.55
124.01
120.90
77.55
77.23
76.91
59.39
23.48
O
S
O
N
5h
ppm 200 175 150 125 100 75 50 25 0
Current Data Parameters
NAME 20110426
EXPNO 3
PROCNO 1
F2 - Acquisition Parameters
Date_ 20110426
Time 8.14
INSTRUM spect
PROBHD 5 mm BBO BB-1H
PULPROG zgpg30
TD 65536
SOLVENT CDCl3
NS 104
DS 0
SWH 25062.656 Hz
FIDRES 0.382426 Hz
AQ 1.3074932 sec
RG 8192
DW 19.950 usec
DE 6.50 usec
TE 299.5 K
D1 2.00000000 sec
d11 0.03000000 sec
DELTA 1.89999998 sec
MCREST 0.00000000 sec
MCWRK 0.01500000 sec
CHANNEL f1
NUC1 13C
P1 10.00 usec
PL1 5.30 dB
SFO1 100.6242895 MHz
CHANNEL f2
CPDPRG2 waltz16
NUC2 1H
PCPD2 90.00 usec
PL2 0.00 dB
PL12 16.50 dB
PL13 19.50 dB
SFO2 400.1319000 MHz
F2 - Processing parameters
SI 32768
SF 100.6127523 MHz
WDW EM
SSB 0
LB 1.00 Hz
GB 0
PC 1.40
1D NMR plot parameters
CX 20.00 cm
CY 11.25 cm
F1P 220.000 ppm
F1 22134.80 Hz
F2P -10.000 ppm
F2 -1006.13 Hz
PPMCM 11.50000 ppm/cm
HZCM 1157.04663 Hz/cm

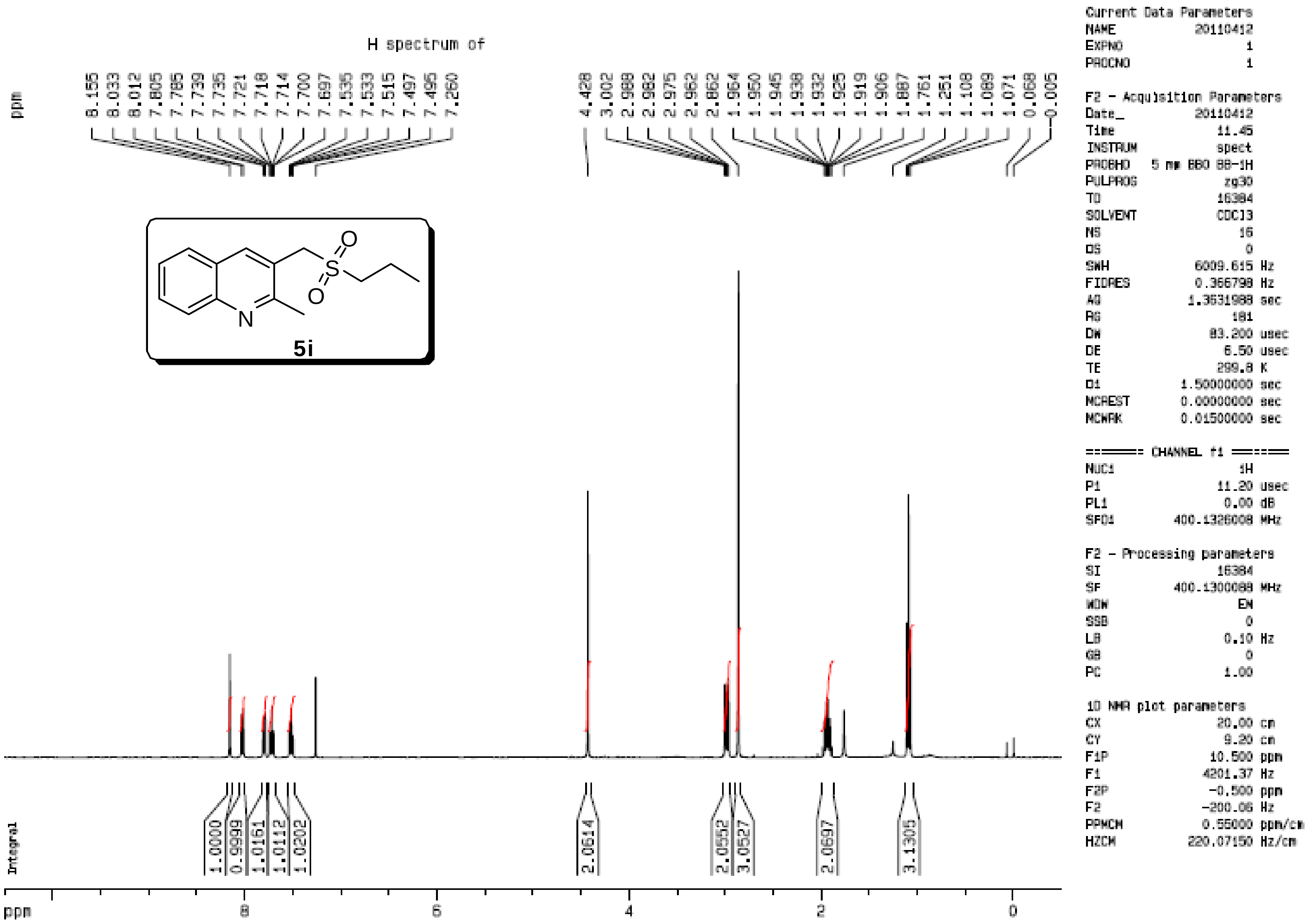
H spectrum of
ppm
8.155 8.033 8.012 7.805 7.785 7.739 7.735 7.721 7.718 7.714 7.700 7.697 7.535 7.533 7.515 7.497 7.495 7.260
4.428 3.002 2.988 2.982 2.975 2.962 2.862 1.964 1.950 1.945 1.938 1.932 1.925 1.919 1.906 1.887 1.761 1.251 1.108 1.089 1.071 0.068 -0.005
N
O
S
O
5i
Integral
1.0000 0.9999 1.0151 1.0112 1.0202 2.0614 2.0552 3.0527 2.0697 3.1305
ppm
8 6 4 2 0
Current Data Parameters
NAME 20110412
EXPNO 1
PROCNO 1
F2 - Acquisition Parameters
Date_ 20110412
Time 11.45
INSTRUM spect
PROBHD 5 mm BBO BB-1H
PULPROG zg30
TD 16384
SOLVENT CDCl3
NS 16
DS 0
SWH 6009.615 Hz
FIDRES 0.366798 Hz
AQ 1.3631988 sec
RG 181
DW 83.200 usec
DE 6.50 usec
TE 299.8 K
D1 1.50000000 sec
MCREST 0.00000000 sec
MCWRK 0.01500000 sec
======== CHANNEL f1 ========
NUC1 1H
P1 11.20 usec
PL1 0.00 dB
SFO1 400.1326008 MHz
F2 - Processing parameters
SI 16384
SF 400.1300088 MHz
WDW EM
SSB 0
LB 0.10 Hz
GB 0
PC 1.00
1D NMR plot parameters
CX 20.00 cm
CY 9.20 cm
F1P 10.500 ppm
F1 4201.37 Hz
F2P -0.500 ppm
F2 -200.06 Hz
PPMCM 0.55000 ppm/cm
HZCM 220.07150 Hz/cm

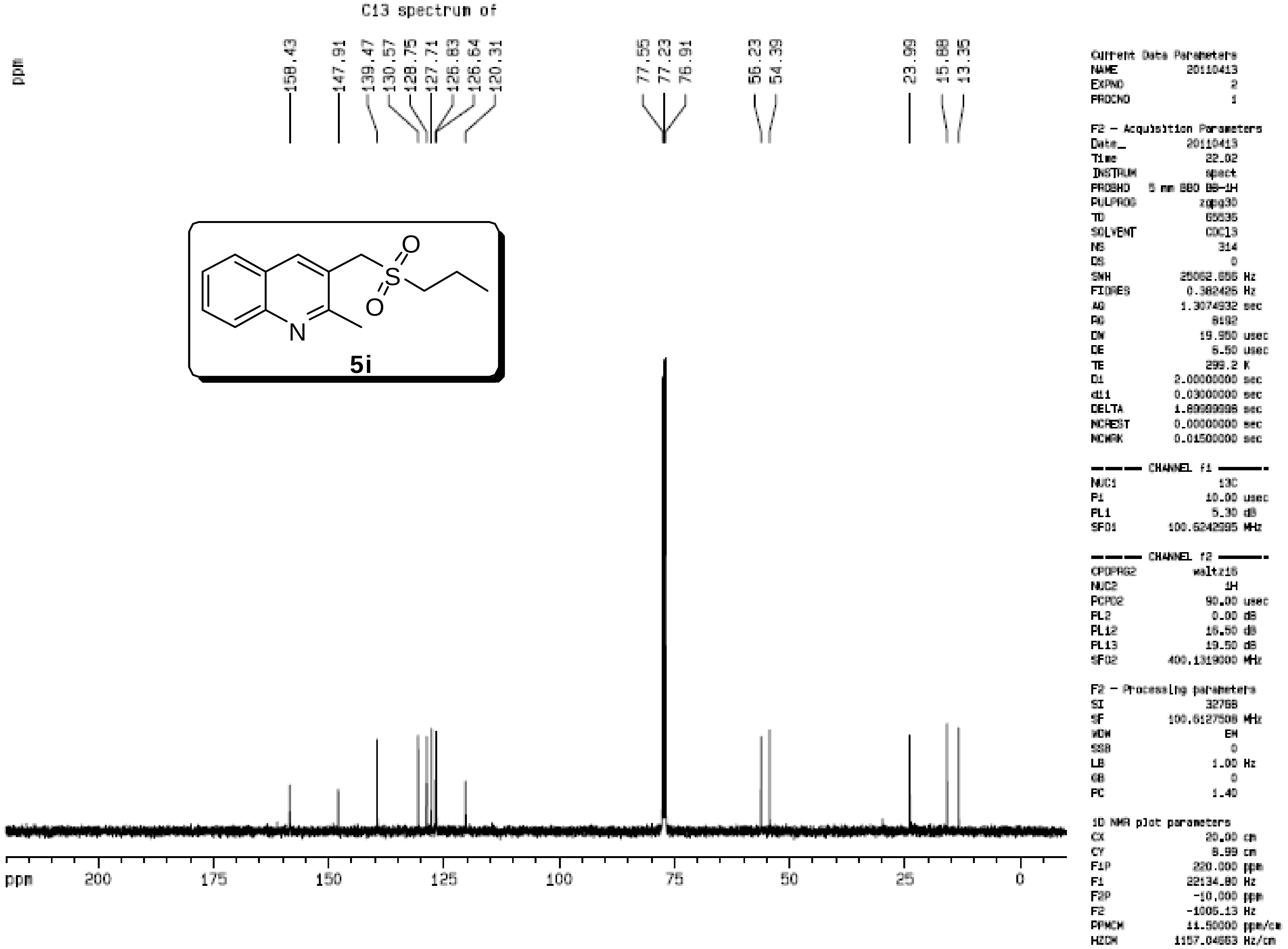
C13 spectrum of
ppm
158.43
147.91
139.47
130.57
128.75
127.71
126.83
126.64
120.31
77.55
77.23
76.91
56.23
54.39
23.99
15.88
13.35
O
S
O
N
5i
ppm 200 175 150 125 100 75 50 25 0
Current Data Parameters
NAME 20110413
EXPNO 2
PROCNO 1
F2 - Acquisition Parameters
Date_ 20110413
Time 22.02
INSTRUM spect
PROBHD 5 mm BBO BB-1H
PULPROG zgpg30
TD 65536
SOLVENT CDCl3
NS 314
DS 0
SWH 25062.656 Hz
FIDRES 0.382425 Hz
AQ 1.3074932 sec
RG 8192
DW 19.950 usec
DE 6.50 usec
TE 298.2 K
D1 2.00000000 sec
d11 0.03000000 sec
DELTA 1.89999998 sec
MCREST 0.00000000 sec
MCWRK 0.01500000 sec
CHANNEL f1
NUC1 13C
P1 10.00 usec
PL1 5.30 dB
SFO1 100.6242695 MHz
CHANNEL f2
CPDPRG2 waltz16
NUC2 1H
PCPD2 90.00 usec
PL2 0.00 dB
PL12 16.50 dB
PL13 19.50 dB
SFO2 400.1319000 MHz
F2 - Processing parameters
SI 32768
SF 100.6127508 MHz
WDW EM
SSB 0
LB 1.00 Hz
GB 0
PC 1.40
1D NMR plot parameters
CX 20.00 cm
CY 8.99 cm
F1P 220.000 ppm
F1 22134.80 Hz
F2P -10.000 ppm
F2 -1006.13 Hz
PPMCM 11.50000 ppm/cm
HZCM 1157.04663 Hz/cm

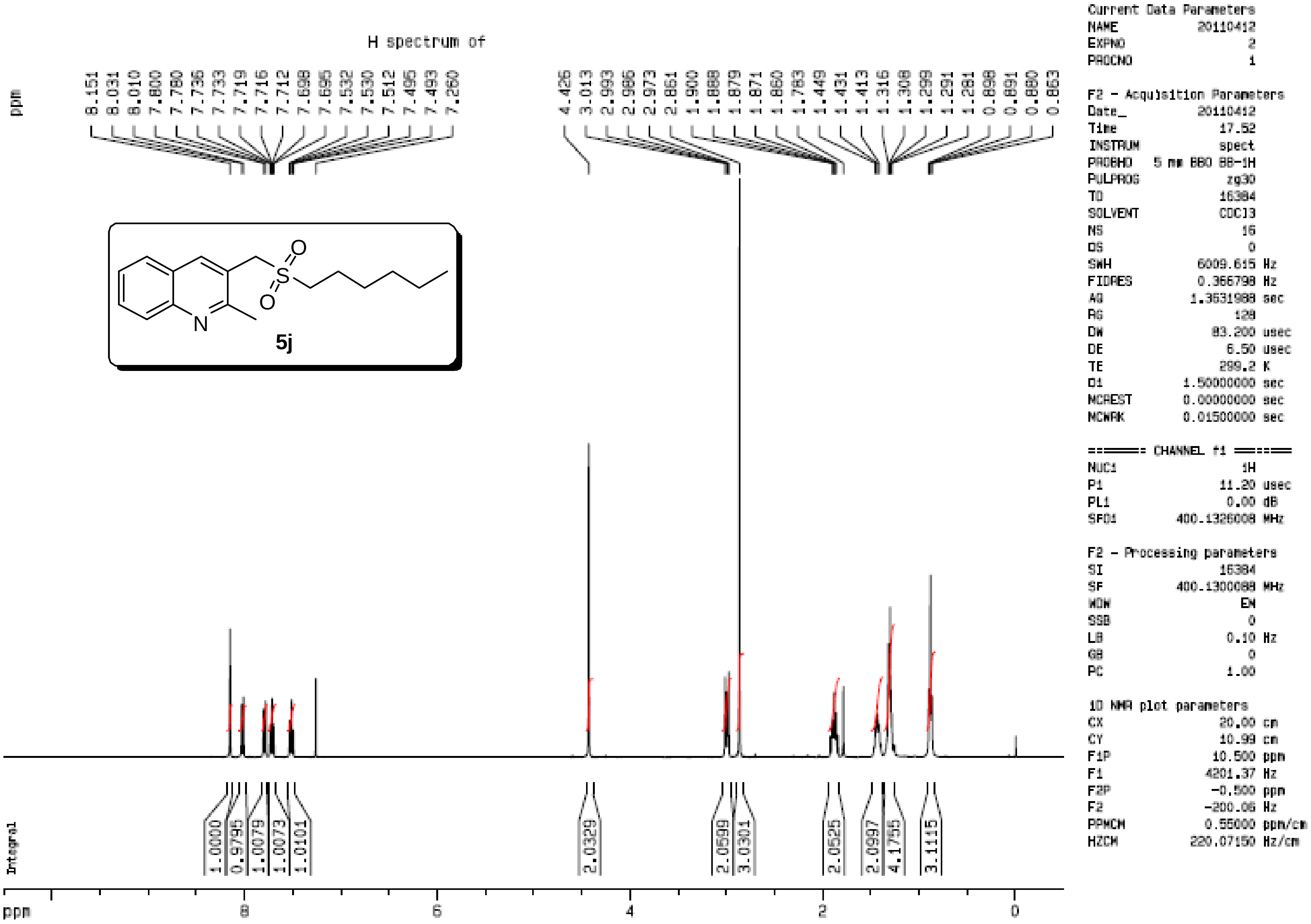
H spectrum of
ppm
8.151
8.031
8.010
7.800
7.780
7.736
7.733
7.719
7.716
7.712
7.698
7.695
7.532
7.530
7.512
7.495
7.493
7.260
4.425
3.013
2.993
2.986
2.973
2.861
1.900
1.888
1.879
1.871
1.860
1.783
1.449
1.431
1.413
1.316
1.308
1.299
1.291
1.281
0.898
0.891
0.880
0.863
O
S
O
N
5j
Integral
1.0000
0.9795
1.0079
1.0073
1.0101
2.0329
2.0599
3.0301
2.0525
2.0997
4.1756
3.1115
ppm
8
6
4
2
0
Current Data Parameters
NAME 20110412
EXPNO 2
PROCNO 1
F2 - Acquisition Parameters
Date_ 20110412
Time 17.52
INSTRUM spect
PROBHD 5 mm BBO BB-1H
PULPROG zg30
TD 16384
SOLVENT CDCl3
NS 16
DS 0
SWH 6009.615 Hz
FIDRES 0.366798 Hz
AQ 1.3631988 sec
RG 128
DW 83.200 usec
DE 6.50 usec
TE 299.2 K
D1 1.50000000 sec
MCREST 0.00000000 sec
MCWRK 0.01500000 sec
======== CHANNEL f1 ========
NUC1 1H
P1 11.20 usec
PL1 0.00 dB
SFO1 400.1326008 MHz
F2 - Processing parameters
SI 16384
SF 400.1300088 MHz
WDW EM
SSB 0
LB 0.10 Hz
GB 0
PC 1.00
1D NMR plot parameters
CX 20.00 cm
CY 10.99 cm
F1P 10.500 ppm
F1 4201.37 Hz
F2P -0.500 ppm
F2 -200.06 Hz
PPMCM 0.55000 ppm/cm
HZCM 220.07150 Hz/cm

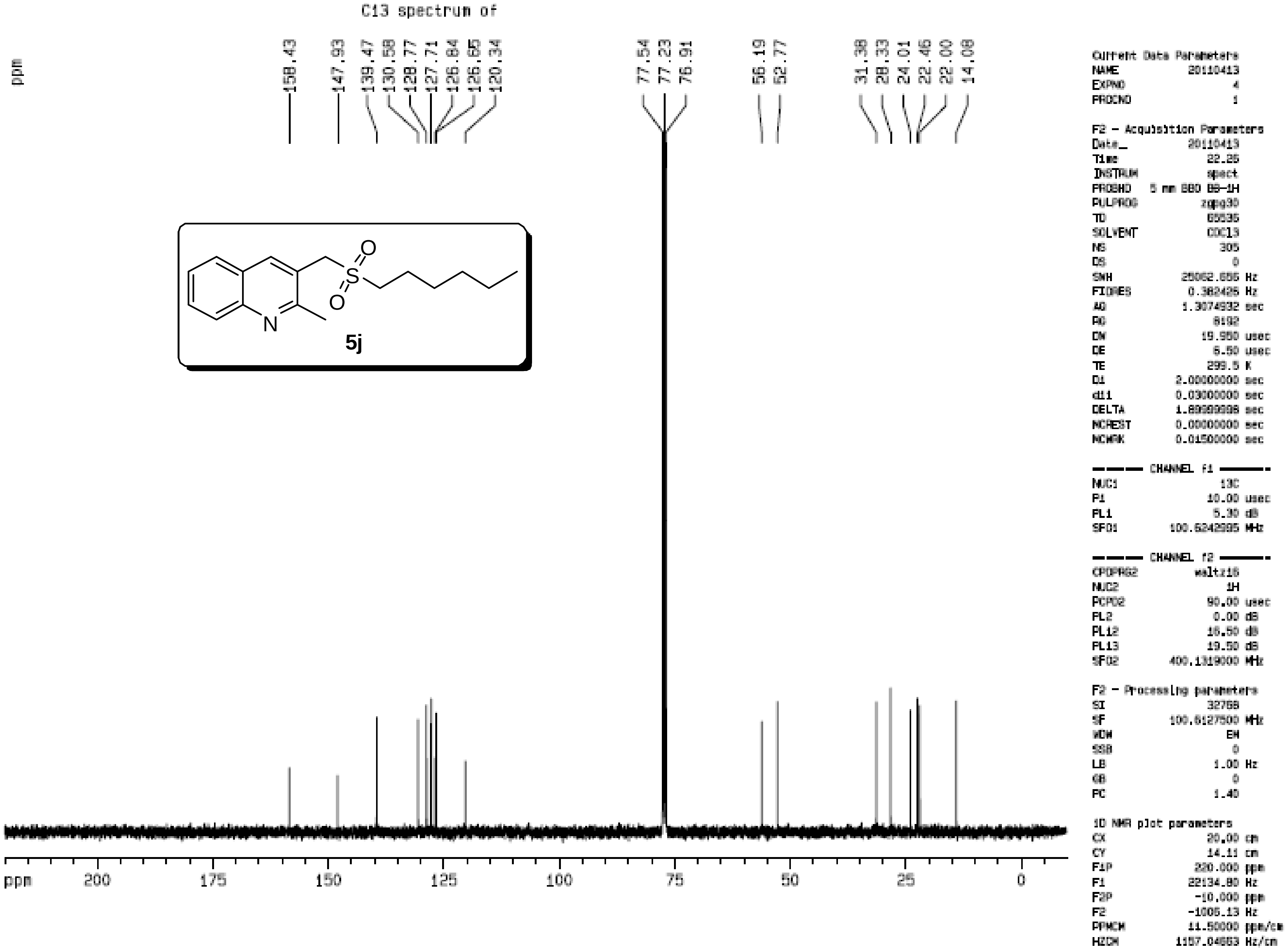
C13 spectrum of
158.43
147.93
139.47
130.58
128.77
127.71
126.84
126.65
120.34
77.54
77.23
76.91
56.19
52.77
31.38
28.33
24.01
22.46
22.00
14.08
5j
ppm
200
175
150
125
100
75
50
25
0
Current Data Parameters
NAME 20110413
EXPNO 4
PROCNO 1
F2 - Acquisition Parameters
Date_ 20110413
Time 22.26
INSTRUM spect
PROBHD 5 mm BBO BB-1H
PULPROG zgpg30
TD 65536
SOLVENT CDCl3
NS 305
DS 0
SWH 25062.656 Hz
FIDRES 0.382426 Hz
AQ 1.3074932 sec
RG 8192
DW 19.950 usec
DE 6.50 usec
TE 299.5 K
D1 2.00000000 sec
d11 0.03000000 sec
DELTA 1.89999998 sec
MCREST 0.00000000 sec
MCWRK 0.01500000 sec
CHANNEL f1
NUC1 13C
P1 10.00 usec
PL1 5.30 dB
SFO1 100.6242995 MHz
CHANNEL f2
CPDPRG2 waltz16
NUC2 1H
PCPD2 90.00 usec
PL2 0.00 dB
PL12 16.50 dB
PL13 19.50 dB
SFO2 400.1319000 MHz
F2 - Processing parameters
SI 32768
SF 100.6127500 MHz
WDW EM
SSB 0
LB 1.00 Hz
GB 0
PC 1.40
1D NMR plot parameters
CX 20.00 cm
CY 14.11 cm
F1P 220.000 ppm
F1 22134.80 Hz
F2P -10.000 ppm
F2 -1006.13 Hz
PPMCM 11.50000 ppm/cm
HZCM 1157.04663 Hz/cm

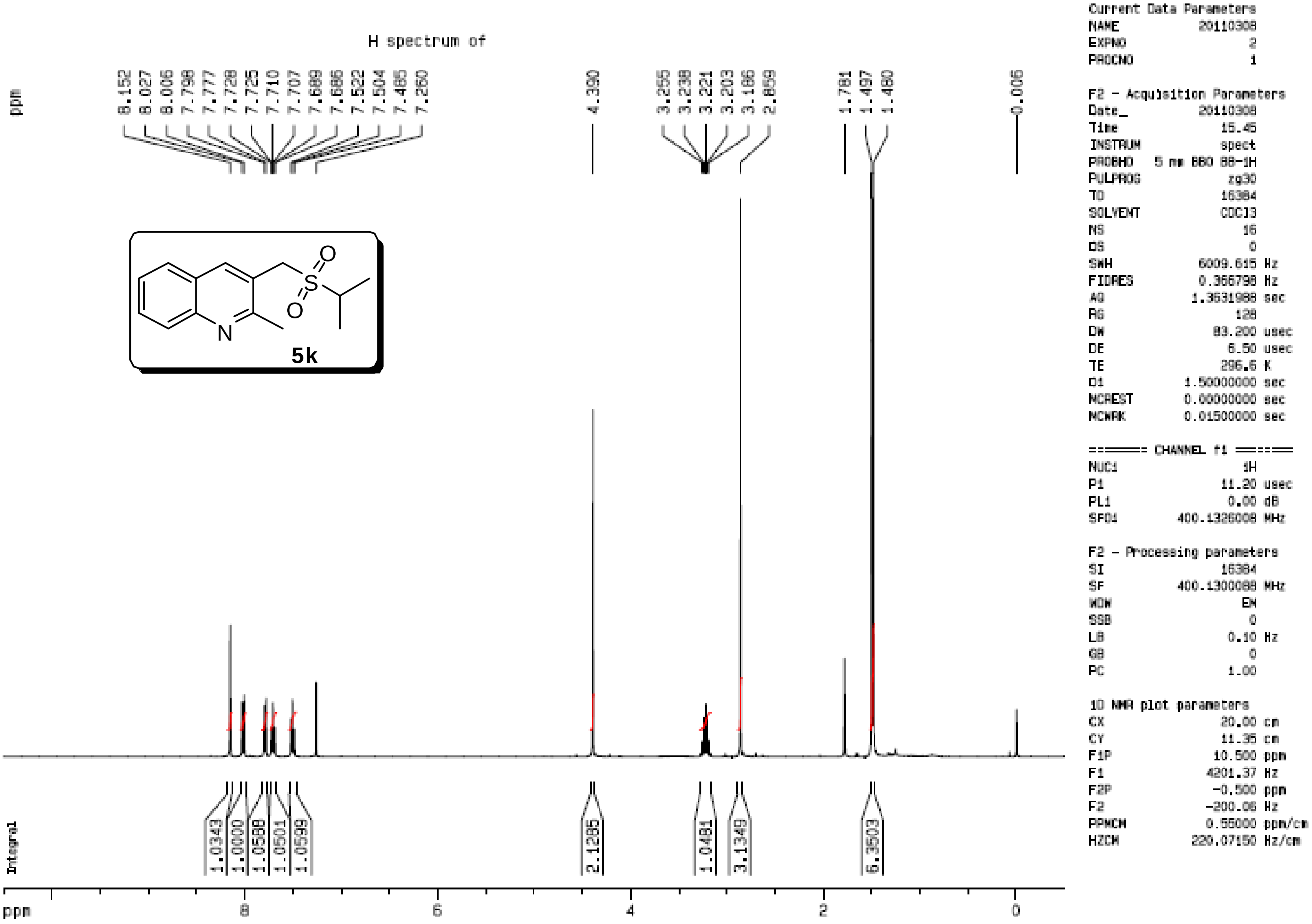
H spectrum of
5k
ppm
8.152 8.027 8.006 7.798 7.777 7.728 7.725 7.710 7.707 7.689 7.686 7.522 7.504 7.485 7.260
4.390
3.255 3.238 3.221 3.203 3.186 2.859
1.761 1.497 1.480
0.006
Integral
1.0343 1.0000 1.0588 1.0501 1.0599 2.1285 1.0481 3.1349 6.3503
ppm
8 6 4 2 0
Current Data Parameters
NAME 20110308
EXPNO 2
PROCNO 1
F2 - Acquisition Parameters
Date_ 20110308
Time 15.45
INSTRUM spect
PROBHD 5 mm BBO BB-1H
PULPROG zg30
TD 16384
SOLVENT CDCl3
NS 16
DS 0
SWH 6009.615 Hz
FIDRES 0.366798 Hz
AQ 1.3631988 sec
RG 128
DW 83.200 usec
DE 6.50 usec
TE 296.6 K
D1 1.50000000 sec
MCREST 0.00000000 sec
MCWRK 0.01500000 sec
======== CHANNEL f1 ========
NUC1 1H
P1 11.20 usec
PL1 0.00 dB
SFO1 400.1326008 MHz
F2 - Processing parameters
SI 16384
SF 400.1300088 MHz
WDW EM
SSB 0
LB 0.10 Hz
GB 0
PC 1.00
1D NMR plot parameters
CX 20.00 cm
CY 11.35 cm
F1P 10.500 ppm
F1 4201.37 Hz
F2P -0.500 ppm
F2 -200.06 Hz
PPMCM 0.55000 ppm/cm
HZCM 220.07150 Hz/cm

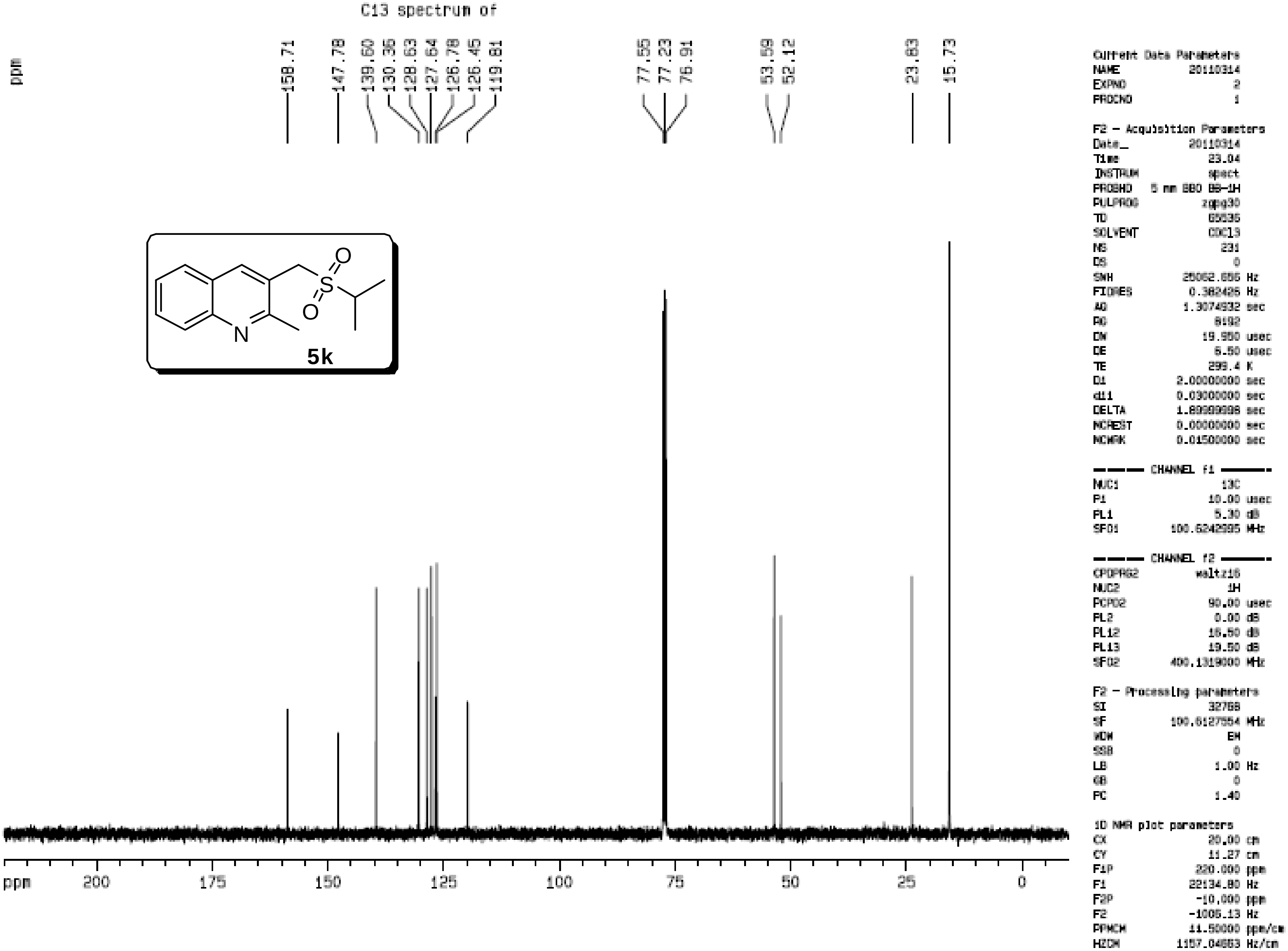
C13 spectrum of
ppm
158.71
147.78
139.60
130.36
128.63
127.64
126.78
126.45
119.81
77.55
77.23
76.91
53.59
52.12
23.83
15.73
N
S
O
O
5k
ppm 200 175 150 125 100 75 50 25 0
Current Data Parameters
NAME 20110314
EXPNO 2
PROCNO 1
F2 - Acquisition Parameters
Date_ 20110314
Time 23.04
INSTRUM spect
PROBHD 5 mm BBO BB-1H
PULPROG zgpg30
TD 65536
SOLVENT CDCl3
NS 231
DS 0
SWH 25062.656 Hz
FIDRES 0.382426 Hz
AQ 1.3074932 sec
RG 8192
DW 19.950 usec
DE 6.50 usec
TE 299.4 K
D1 2.00000000 sec
d11 0.03000000 sec
DELTA 1.89999998 sec
MCREST 0.00000000 sec
MCWRK 0.01500000 sec
CHANNEL f1
NUC1 13C
P1 10.00 usec
PL1 5.30 dB
SFO1 100.6242995 MHz
CHANNEL f2
CPDPRG2 waltz16
NUC2 1H
PCPD2 90.00 usec
PL2 0.00 dB
PL12 16.50 dB
PL13 19.50 dB
SFO2 400.1319000 MHz
F2 - Processing parameters
SI 32768
SF 100.6127554 MHz
WDW EM
SSB 0
LB 1.00 Hz
GB 0
PC 1.40
1D NMR plot parameters
CX 20.00 cm
CY 11.27 cm
F1P 220.000 ppm
F1 22134.80 Hz
F2P -10.000 ppm
F2 -1006.13 Hz
PPMCM 11.50000 ppm/cm
HZCM 1157.04663 Hz/cm

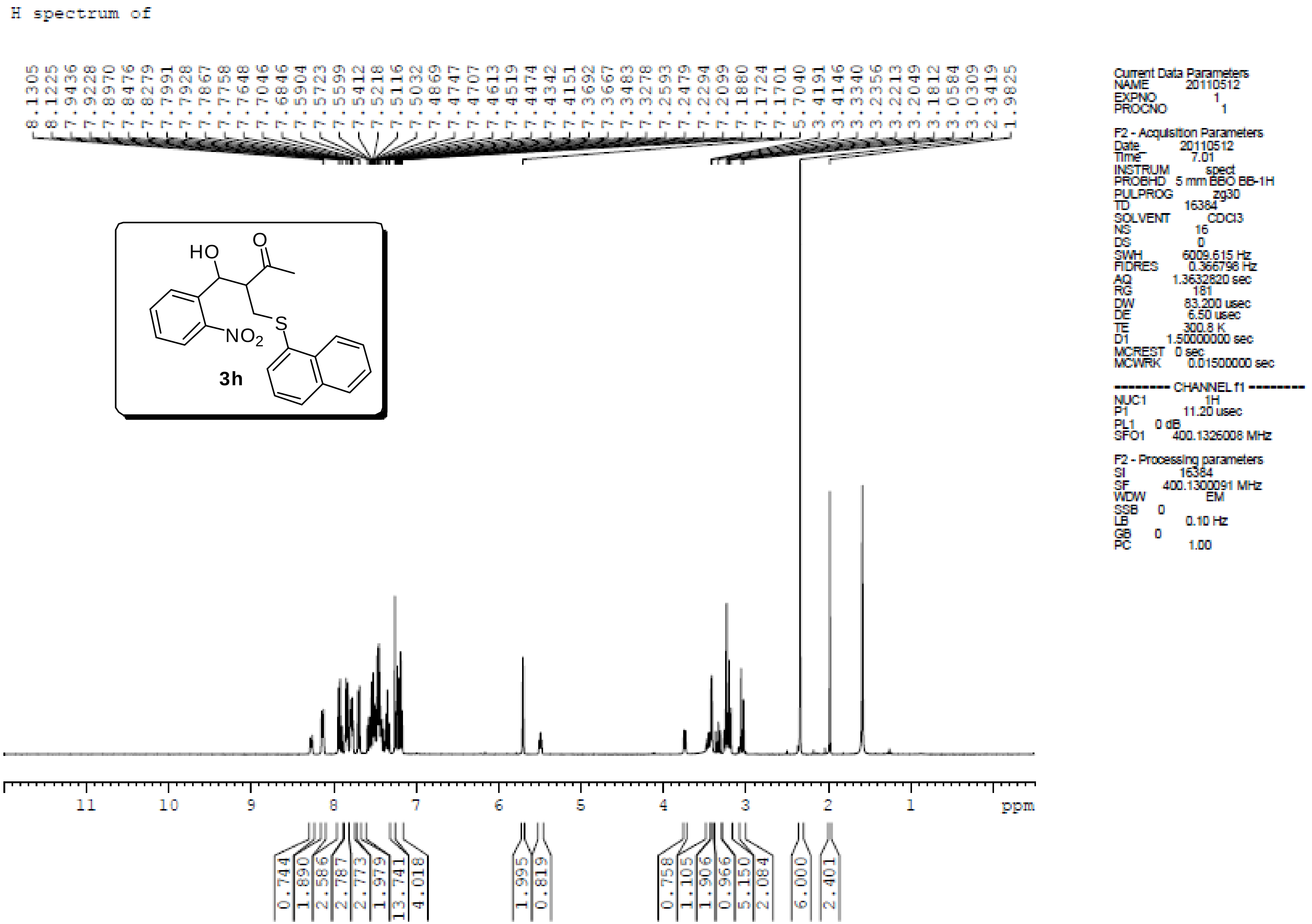
H spectrum of
HO
O
NO_2
S
3h
Current Data Parameters
NAME 20110512
EXPNO 1
PROCNO 1
F2 - Acquisition Parameters
Date 20110512
Time 7.01
INSTRUM spect
PROBHD 5 mm BBO BB-1H
PULPROG zg30
TD 16384
SOLVENT CDCl3
NS 16
DS 0
SWH 6009.615 Hz
FIDRES 0.366798 Hz
AQ 1.3632820 sec
RG 181
DW 83.200 usec
DE 6.50 usec
TE 300.8 K
D1 1.50000000 sec
MCREST 0 sec
MCWRK 0.01500000 sec
======== CHANNEL f1 ========
NUC1 1H
P1 11.20 usec
PL1 0 dB
SFO1 400.1326008 MHz
F2 - Processing parameters
SI 16384
SF 400.1300091 MHz
WDW EM
SSB 0
LB 0.10 Hz
GB 0
PC 1.00
ppm

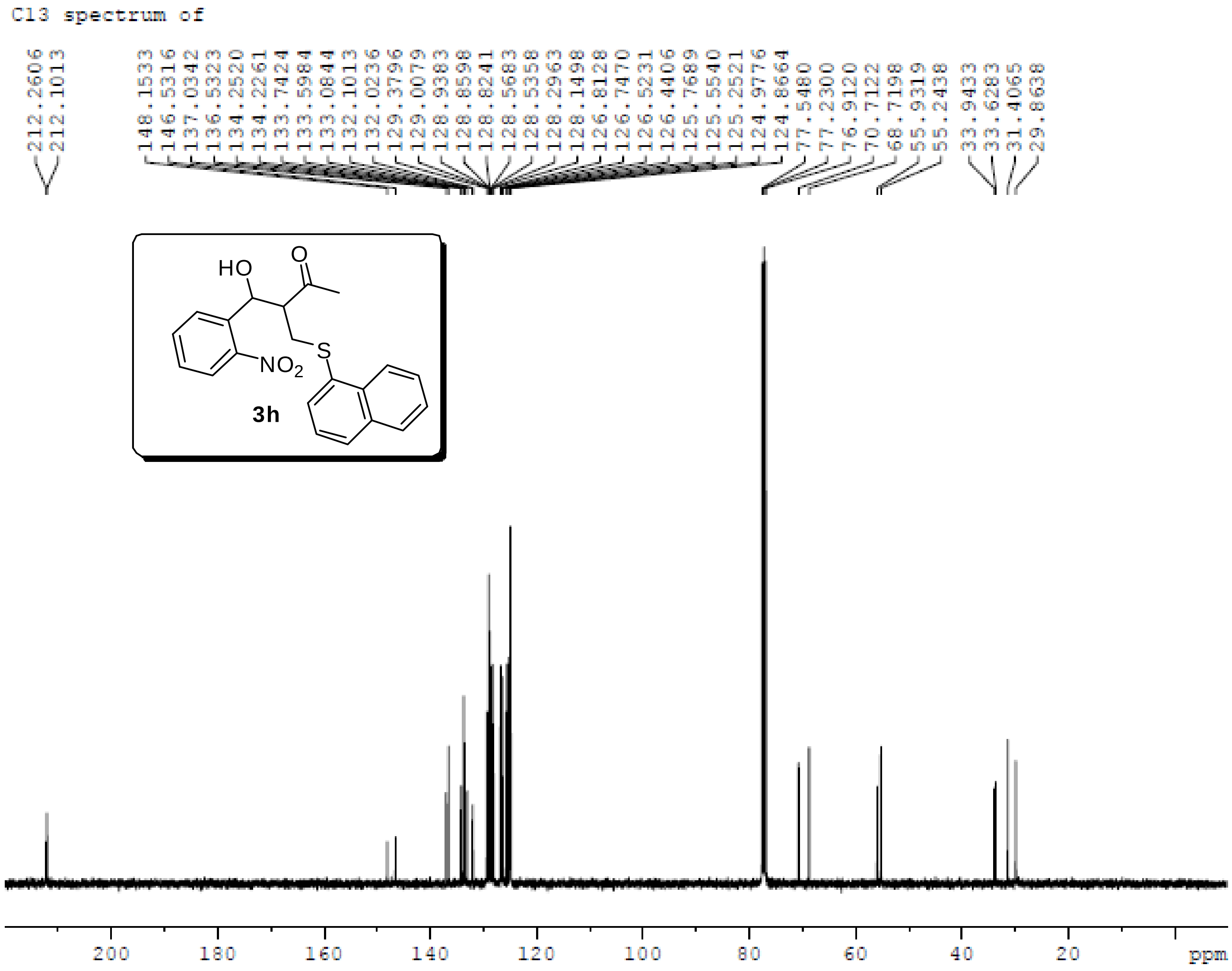

Current Data Parameters
NAME 20110512
EXPNO 4
PROCNO 1

F2 - Acquisition Parameters
Date_ 20110512
Time 7.09
INSTRUM spect
PROBHD 5 mm BBO BB-1H
PULPROG zgpg30
TD 65536
SOLVENT CDCl3
NS 541
DS 0
SWH 25062.656 Hz
FIDRES 0.382426 Hz
AQ 1.3075131 sec
RG 8192
DW 19.950 usec
DE 6.50 usec
TE 302.0 K
D1 2.00000000 sec
d11 0.03000000 sec
DELTA 1.89999998 sec
MCREST 0 sec
MCWRK 0.01500000 sec

======== CHANNEL f1 ========
NUC1 13C
P1 10.00 usec
PL1 5.30 dB
SFO1 100.6242995 MHz

======== CHANNEL f2 ========
CPDPRG2 waltz16
NUC2 1H
PCPD2 90.00 usec
PL2 0 dB
PL12 18.50 dB
PL13 19.50 dB
SFO2 400.1319000 MHz

F2 - Processing parameters
SI 32768
SF 100.6127512 MHz
WDW EM
SSB 0
LB 1.00 Hz
GB 0
PC 1.40

Crystallographic description of 3h:

Identification code	a12327	
Empirical formula	$C_{21} H_{19} N O_4 S$	
Formula weight	381.43	
Temperature	200(2) K	
Wavelength	0.71073 Å	
Crystal system	Monoclinic	
Space group	P 21/c	
Unit cell dimensions	a = 32.290(4) Å	α = 90°.
	b = 7.4466(9) Å	β = 103.742(6)°
	c = 15.7324(16) Å	γ = 90°.
Volume	3674.6(7) $Å^3$	
Z	8	
Density (calculated)	1.379 Mg/m^3	
Absorption coefficient	0.204 mm^{-1}	
F(000)	1600	
Crystal size	0.44 × 0.32 × 0.08 mm^3	
Theta range for data collection	1.30 to 25.02°.	
Index ranges	$-29 \leq h \leq 38, -7 \leq k \leq 8, -16 \leq l \leq 18$	
Reflections collected	13232	
Independent reflections	6139 [R(int) = 0.0734]	
Completeness to theta = 25.02°	94.4%	
Absorption correction	multi-scan	
Max. and min. transmission	0.9839 and 0.9158	
Refinement method	Full-matrix least-squares on F^2	
Data / restraints / parameters	6139 / 0 / 277	
Goodness-of-fit on F^2	1.183	
Final R indices [I>2sigma(I)]	R1 = 0.2457, wR2 = 0.4913	
R indices (all data)	R1 = 0.2811, wR2 = 0.5062	
Largest diff. peak and hole	1.222 and −0.713 e.$Å^{-3}$	
